# Unveiling active sites and the cooperative role of non-thermal plasma and copper–zinc catalysts in the hydrogenation of CO_2_ to methanol

**DOI:** 10.1038/s41929-025-01477-5

**Published:** 2026-02-13

**Authors:** Shanshan Xu, Matthew E. Potter, Raquel Simancas, Lucy Costley-Wood, Boya Qiu, Xuzhao Liu, Cristina Stere, M. Asunción Molina, Danial Farooq, Floriana Tuna, Dingyue Zhang, Shuanglin Zhang, Huanhao Chen, Shengzhe Ding, Xinrui Wang, Sarayute Chansai, Matthew Lindley, Sarah J. Haigh, Armando Ibraliu, Lan Lan, Piu Chawdhury, Mariyam Bi, Otis Leahair, Yilai Jiao, Min Hu, Qiang Liu, Toru Wakihara, Xiaolei Fan, Andrew M. Beale, Christopher Hardacre

**Affiliations:** 1 Department of Chemical Engineering, The University of Manchester, Manchester, UK; 2 Department of Materials, The University of Manchester, Manchester, UK; 3 Institute of Sustainability and Climate Change, Department of Chemistry, University of Bath, Bath, UK; 4 Research Complex at Harwell, Harwell, Didcot, UK; 5 Institute of Engineering Innovation, The University of Tokyo, Bunkyo-ku, Tokyo, Japan; 6 Department of Chemistry, University College London, London, UK; 7 Departmentof Chemistry and Photon Science Institute, The University of Manchester, Manchester, UK; 8 State Key Laboratory of Materials-Oriented Chemical Engineering, College of Chemical Engineering, Nanjing Tech University, Nanjing, China; 9 Wenzhou Key Laboratory of Novel Optoelectronic and Nano Materials, Institute of Wenzhou, Zhejiang University, Wenzhou, China; 10 Shenyang National Laboratory for Materials Science, Institute of Metal Research, Chinese Academy of Sciences, Shenyang, China; 11 Department of Electrical and Electronic Engineering, The University of Manchester, Manchester, UK

**Keywords:** Heterogeneous catalysis, Catalytic mechanisms, Process chemistry

## Abstract

Methanol synthesis via non-thermal plasma (NTP) catalytic CO_2_ hydrogenation provides a sustainable approach to chemical and fuel production with potential in carbon emissions reduction. However, the underlying mechanisms remain unclear. Here we evaluate the mechanism of NTP-catalytic CO_2_ hydrogenation over Cu–Zn/ZSM-5 through operando X-ray absorption spectroscopy, diffuse reflectance infrared Fourier transform spectroscopy and in situ X-ray pair distribution function. We found that Zn enhances Cu dispersion and reducibility, as well as forming active Cu/ZnO interfacial sites. Beyond the conventional formate pathway on metallic Cu, these interfaces enable an additional CO hydrogenation route, enhancing methanol yield. NTP also promotes gas-phase CO_2_ dissociation to CO, bypassing the reverse water–gas shift step required in thermal catalysis. No Cu/Zn alloy formation was observed, underscoring the importance of metallic Cu and Cu/ZnO interfaces under NTP conditions. Furthermore, NTP stabilizes reduced Cu species, preventing re-oxidation and ensuring sustained catalytic activity. These findings advance the mechanistic understanding of NTP-assisted catalysis.

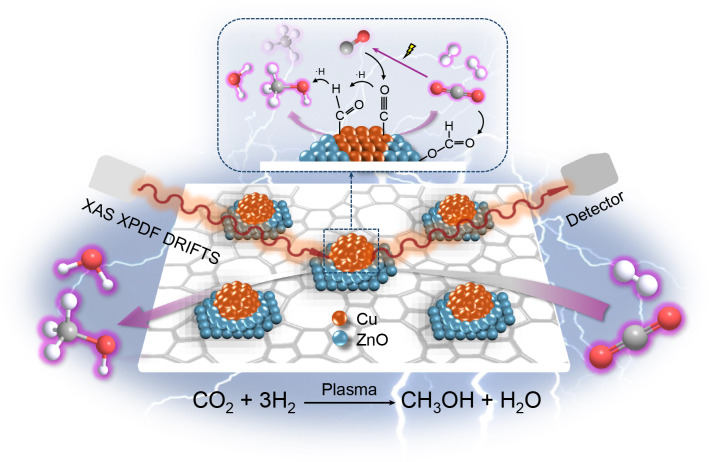

## Abstract (web summary)

Plasmas can unlock unconventional reactivity for established catalytic systems, but understanding the resulting mechanistic changes is a complex endeavour. Here in situ characterization techniques allow us to rationalize the promotional role of non-thermal plasma on the catalytic hydrogenation of CO_2_ to methanol on Cu–Zn systems.

## Main

Methanol synthesis via catalytic CO_2_ hydrogenation is a key option for sustainable chemical and fuel production via carbon cycling, possibly containing the social and environmental impacts from CO_2_ emissions. Given the inherent intermittency of renewable energy sources, distributed technologies powered by renewable electricity are needed to ensure flexible operation and to reduce the cost associated with CO_2_ capture, transportation and storage^1–3^. Compared with conventional thermal-catalytic CO_2_ hydrogenation to methanol (normally operated at 220–300 °C and 50–150 bar), electrified non-thermal plasma (NTP) catalysis enables methanol synthesis under milder conditions (for example, atmospheric pressure) with a short induction time due to rapid activation within the gas discharge^4,5^, and is amenable to green methanol production using sustainable electricity and green hydrogen. For example, one study reported a methanol space–time yield (STY) of 1.0 mmol g^−1^ h^−1^ over a Cu/γ-Al_2_O_3_ catalyst using a dielectric barrier discharge (DBD) reactor with the water-cooled electrode at room temperature operating at 10 W (ref. ^6^). Another research group demonstrated a 0.14 mmol g^−1^ h^−1^ methanol STY using a MnO*x*/ZrO_2_ catalyst under NTP conditions operating at 5 W (ref. ^7^). Despite these promising proof-of-concept reports, little is known about the underlying mechanisms for methanol formation under NTP conditions, for example, the interplay between NTP and the active sites or the evolution of catalyst structure (for example, the chemical state and alloy formation) due to the NTP discharge. Recently, using the density functional theory method, the CO_2_ hydrogenation over a Cu/γ-Al_2_O_3_ catalyst for methanol synthesis under plasma conditions was studied examining the individual energy barriers for different pathways and proposed interfacial active sites between Cu and γ-Al_2_O_3_ responsible for activation of CO_2_ and stabilization of intermediates^8^. Similarly, density functional theory calculations on the Ni–Co/Al_2_O_3_ catalyst in one study revealed that the metallic Ni–Co interface was the active site for CO_2_ adsorption and hydrogenation^9^.

NTP catalysis is a complex combination of chemical and physical phenomena including, not exhaustively, ionization or activation of molecules, species transport in different phases, adsorption and desorption, species formation and dissociation via surface and gas-phase reactions. Therefore, in addition to modelling approaches, the applications of in situ and operando characterization techniques are indispensable to obtain the mechanistic information of various phenomena in NTP-catalytic systems, which allows rational design and optimization of bespoke catalytic materials and NTP-catalytic systems. This was exemplified by recent relevant studies of surface reactions and reaction pathways during the NTP-catalytic methanation of CO_2_ using in situ and operando diffuse reflectance infrared (IR) Fourier transform spectroscopy (DRIFTS)^10,11^, and also by the evidence of plasma-induced heating of Pd nanoparticles (NPs) in NTP-catalytic CH_4_ oxidation by in situ X-ray absorption fine structure (XAFS) measurements^12^.

In this work, targeting methanol synthesis under mild conditions, we have developed a series of copper–zinc catalysts supported on ZSM-5 zeolite for NTP-catalytic CO_2_ hydrogenation. The activity data show that the combination of Cu and Zn under the NTP conditions delivers a catalytic performance that is greater than that of either element alone, achieving a methanol selectivity of ~37.4% and methanol STY of ~5.1 mmol g^−1^ h^−1^. Extensive characterization including operando X-ray absorption spectroscopy (XAS), in situ X-ray pair distribution function (XPDF) and operando DRIFTS confirm the role of Zn addition in modifying the Cu phase and both metallic Cu and the Cu/ZnO_*x*_ interfacial sites contributed to the increased activity, promoting multiple reaction pathways to enhance methanol synthesis in the NTP catalysis. Operando XAS characterization of the NTP catalysis revealed the role of NTP in sustaining and regenerating the active state of Cu species for CO_2_ hydrogenation and the cooperative role of zinc species in hindering the re-oxidation of Cu. This work provides a deep understanding of NTP-catalytic CO_2_ hydrogenation regarding the identification of active sites, the interplays between plasma, catalyst and surface reactions, and the reaction mechanism, serving as a guideline for developing mature NTP catalysis technology for potential practical adoptions.

## Results

### Cu/Zn promoted methanol synthesis in NTP-catalytic CO_2_ hydrogenation

**Fig. 1:**
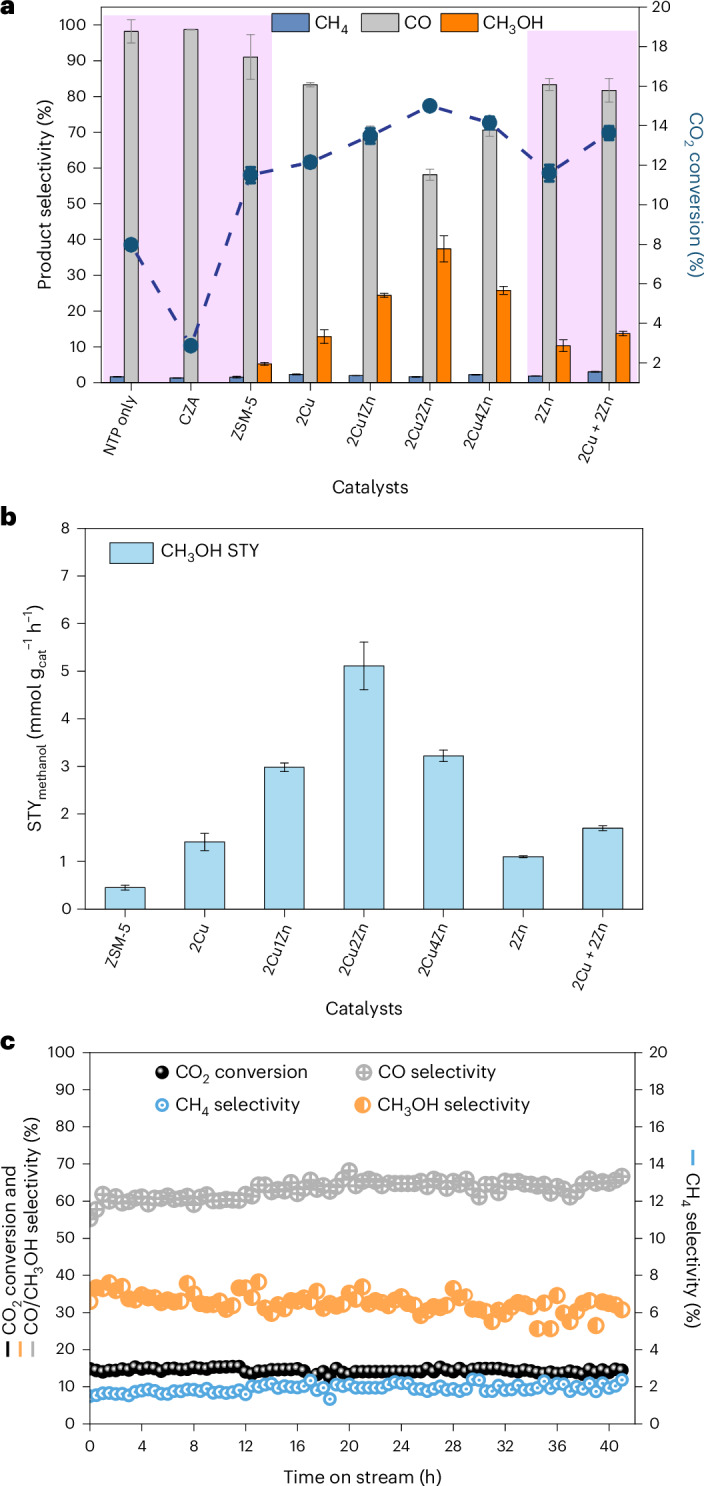
Catalytic performance of the 2Cu*x*Zn catalysts in NTP-catalytic CO_2_ hydrogenation. **a**, CO_2_ conversion and product selectivity for control systems (shaded in pink) and the 2Cu*x*Zn catalysts under NTP conditions. **b**, Methanol STY of the selected catalysts from **a** (average values were calculated from measurements performed in triplicate; the error bars represent the standard deviation). **c**, Longevity test of 2Cu2Zn in the NTP-catalytic CO_2_ hydrogenation. Experimental conditions: NTP at 14 W; gas feed 25%CO_2_/75%H_2_, total flow rate 40 ml min^−1^. Source data

Multicomponent copper/zinc oxide/alumina (CZA; Cu/ZnO/Al_2_O_3_) catalysts are widely used in industry in thermal-catalytic methanol synthesis (using CO + H_2_ + CO_2_ as the reaction feed), where the Cu and Zn cooperation enhances the methanol formation^13,14^. However, the commercial CZA catalyst (by Alfa Aesar) achieved a notably low CO_2_ conversion of ~2.9% under the NTP conditions, possibly due to its low surface micro-discharge^15^, necessitating a redesign of the catalysts specifically for NTP-catalytic conditions. Herein, a series of bimetallic Cu and Zn on ZSM-5 catalysts were prepared by wet impregnation. The Cu was kept constant at 2 wt% loading in all the synthesized catalysts and the Zn loading was varied. The bimetallic catalysts are denoted as 2Cu*x*Zn (where *x* is the theoretical Zn wt% of 1, 2 or 4). ZSM-5 was used as the catalyst support due to its ability to promote the chemistry in plasma discharge (Fig. 1a). The 2 wt% monometallic catalysts (2Cu and 2Zn) were also synthesized as control specimens using the same method, and the relevant characterizations of these catalysts (including the bare ZSM-5 support) are presented in Table 1, Supplementary Figs. 1 and 2 and Supplementary Tables 1 and 2. These data show that metal incorporation did not substantially affect the physical properties of the ZSM-5 system (for example, MFI-topology, surface area and pore volume). NTP-catalytic CO_2_ hydrogenation was performed in a bespoke DBD reactor with oil coolant to maintain a constant system temperature of 15–20 °C (Supplementary Fig. 3). All the supported metal catalysts underwent a reductive pretreatment at 400 °C before the catalytic testing, with the optimal reduction temperature based on the preliminary activity data (Supplementary Fig. 4). Figure 1 shows that gas discharge itself (that is, NTP only, without a catalyst) encouraged CO_2_ dissociation, resulting in a CO_2_ conversion of ~8.0% and forming CO together with a small amount of CH_4_ (<2%). When the bare ZSM-5 support was packed in the discharge zone, a selectivity to methanol of ~5.3% was achieved, possibly due to the reaction between the surface carbonates (via CO_2_ adsorption) and NTP activated hydrogen species in the gas discharge^16^. The 2Zn catalyst was found to have a higher methanol selectivity of ~10.3% than bare ZSM-5 support, suggesting that the oxygen vacancy from ZnO might promote methanol formation by promoting CO_2_ activations to form intermediates (for example, formate)^17–19^, whereas the 2Cu catalyst improved the CO_2_ conversion to ~12.2% and methanol selectivity to ~12.9%.

**Table 1:** Catalyst performance and the nature of Cu active sites in the 2Cu*x*Zn catalysts

Catalyst	Methanol STY (mmol g^−1^ h^−1^)	Cu/ZnO particle size (nm)	Cu dispersion (%)^a^	Cu^+^–CO (%, Cu/ZnO interface)	Peak (%) (300–310 °C, H_2_-TPR)	Percentage Cu(0) after reduction (XANES^b^)
2Cu	1.41	4.7 ± 1.2	10.8	0	14.4	51 ± 2
2Cu1Zn	2.98	3.3 ± 0.8	25.5	35.5	31.4	59 ± 2
2Cu2Zn	5.11	3.2 ± 0.7	26.4	62.7	50.4	64 ± 2
2Cu4Zn	3.22	3.0 ± 0.8	27.5	71.6	38.8	51 ± 2

^a^Dispersion of Cu calculated by N_2_O-chemsorption (Supplementary Fig. 16).

^b^Calculated by linear combination fitting of the Cu K-edge XANES after in situ H_2_ reduction (400 °C, 30 min), remaining fraction mixture of Cu(I) and Cu(II).

In comparison, combining Cu and Zn resulted in a decrease in CO selectivity and a concomitant increase in the methanol selectivity (Fig. 1a,b and Supplementary Figs. 5–7) for Zn loadings up to 2 wt% (2Cu2Zn). This shows a strong positive correlation between the Zn loading and methanol selectivity (Supplementary Fig. 5). Similar benefits were found for the CO_2_ conversion and methanol STY with the 2Cu2Zn catalyst having the best activity (methanol STY of ~5.1 mmol g^−1^ h^−1^) and methanol selectivity (~37.4%) in the NTP catalysis operated at 14 W (Fig. 1a,b). The synergy observed between Cu and Zn was further evidenced by comparing with the control system of a physical mixture of the 2Cu and 2Zn catalysts (that is, 2Cu + 2Zn), which produced notably less methanol (STY ~1.7 mmol g^−1^ h^−1^) than the 2Cu2Zn system with identical metal content (Fig. 1a,b). Further increasing Zn loading to 4 wt% (that is, 2Cu4Zn) led to a lower CO_2_ conversion (~14.1%), methanol selectivity (~25.5%) and methanol STY (~3.2 mmol g^−1^ h^−1^) compared with the 2Cu2Zn catalyst. It is worth noting that the catalytic activity and selectivity trends observed between the catalysts were independent of the discharge power (11.5–16 W, Supplementary Fig. 6), suggesting the necessity of designing the catalyst with intrinsic high activity under the NTP conditions. The best performing catalyst, 2Cu2Zn, also showed excellent stability under NTP conditions, maintaining CO_2_ conversions of ~14.5% and methanol selectivity slightly decreasing from ~35.7% to ~32.1% over 41 hours on stream (Fig. 1c), which is associated with the mild NTP condition reducing the production of coke and/or sintering of the catalyst (as indicated by temperature-programmed oxidation and thermogravimetric analysis of the spent catalyst in Supplementary Fig. 8)^20^.

For comparison, the 2Cu, 2Cu*x*Zn and 2Zn catalysts were assessed for CO_2_ hydrogenation under thermal conditions, as shown in Supplementary Fig. 9 and Supplementary Table 4. Low temperatures at 50 °C and 100 °C could not activate CO_2_ hydrogenation thermally, thus no CO_2_ conversion and methanol formation (Supplementary Table 4). At 300 °C, 2Cu and 2Cu*x*Zn showed similar CO_2_ conversions of 3.2–4.0%, whereas 2Zn showed a notably low CO_2_ conversion of ~0.3% (Supplementary Fig. 9a). For 2Cu4Zn, its methanol selectivity dropped to ~2.5%, showing that Zn addition did not promote methanol formation under thermal conditions. In addition, stability test was performed using 2Cu2Zn at 300 °C, and catalyst deactivation with CO_2_ conversion decreased from 3.4% to 1.2% in the initial 5 hours was found (Supplementary Fig. 9b), which is associated with metal sintering as evidenced by the postreaction high-angle annular dark-field–scanning transmission electron microscopy (HAADF–STEM) (Supplementary Fig. 10). Hence, the comparison demonstrates that NTP catalysis is beneficial to promote CO_2_ conversion and methanol formation, as well as alleviating catalyst deactivation under mild conditions compared with the thermal-catalytic counterparts (Supplementary Table 5).

Further, temperature dependence of the NTP-catalytic system was assessed as well using the 2Cu and 2Cu2Zn catalyst, as shown in Supplementary Fig. 11. For both catalysts, the CO_2_ conversion increased continuously by increasing the temperature of the plasma reactor, showing the beneficial effect of increasing temperature on improving CO_2_ activation under NTP conditions. However, the methanol selectivity decreased with an increase in temperature, especially >80 °C, as shown in Supplementary Fig. 11. The measured decrease in methanol selectivity albeit high CO_2_ conversions might be attributed to the possible methanol thermal decomposition and thermodynamic limitation^21,22^.

### Role of Zn in modifying the reducibility of bimetallic Cu/Zn catalysts

**Fig. 2:**
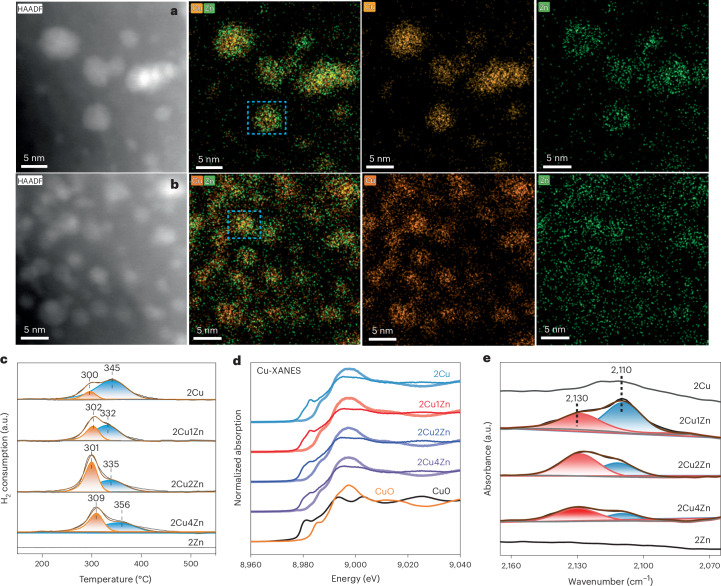
Structural and spectroscopic characterization of 2Cu*x*Zn catalysts. **a**, HAADF–STEM micrographs and corresponding EDX elemental maps of the 2Cu1Zn catalyst after H_2_ reduction at 400 °C. **b**, HAADF–STEM micrographs and corresponding EDX maps of the 2Cu2Zn catalyst after H_2_reduction at 400 °C (blue dashed rectangles highlight Cu/ZnO interactions). **c**, H_2_-TPR of the 2Cu, 2Cu*x*Zn and 2Zn catalysts. **d**, XANES spectra of 2Cu, 2Cu1Zn, 2Cu2Zn and 2Cu4Zn before (lighter colours: green, red, blue and purple, respectively) and after (the corresponding darker colours) H_2_ treatment, compared with metallic Cu foil (black) and Cu–O (orange). **e**, In situ CO-DRIFTS spectra of the H_2_ treated 2Cu, 2Cu*x*Zn and 2Zn catalysts at room temperature. Source data

CO_2_ hydrogenation to methanol is a structure-sensitive reaction using bimetallic Cu/Zn catalysts under conventional thermal conditions. Therein, the nature of the active site is still under debate with two main active structures proposed: Cu/Zn alloying^13,14^ and the interface sites between metallic Cu and ZnO^23^. Relevant mechanistic origins of active sites for methanol synthesis are limited for NTP-catalytic systems, especially for bimetallic catalysts. To investigate this, the as-prepared catalysts (after H_2_ treatment at 400 °C) were characterized to understand the role of Zn in the catalysts under investigation. HAADF–STEM and energy-dispersive X-ray (EDX) spectroscopy (Fig. 2a,b and Supplementary Figs. 12–15) show that Zn addition resulted in the formation of smaller supported NPs, for example, 3–4 nm for 2Cu*x*Zn versus 5–6 nm for 2Cu, which is also consistent with the increase in the calculated Cu dispersion (by N_2_O chemisorption) shown in Table 1 and Supplementary Fig. 16. For the monometallic 2Zn, the electron microscopy (Supplementary Fig. 17) shows the uniform distribution of small ZnO clusters on ZSM-5 (rather than large aggregates). After co-impregnation of the Cu and Zn, the EDX maps in Fig. 2a–b and Supplementary Figs. 13–15 show that species overlap (as indicated by the blue dashed rectangles in Fig. 2a,b), which suggest that Zn species closely interact with the Cu and that NPs observed are bimetallic. It should be noted that the supported Zn species cannot be reduced at 400 °C, as evidenced by the H_2_ temperature-programmed reduction (H_2_-TPR) data in Fig. 2c, suggesting that the NPs observed contain interfacial Cu/ZnO sites in the 2Cu*x*Zn catalysts. Furthermore, after 42 hours on stream, these NPs, in 2Cu2Zn, did not substantially change in size with all found to be ~3.7 nm (Supplementary Fig. 18), demonstrating that NTP activation can reduce metal sintering compared with thermal processes^24,25^. The H_2_-TPR shows that Zn addition affected the reducibility of Cu species. For example, the monometallic 2Cu (Fig. 2c) presents two main peaks at ~300 °C and ~345 °C, corresponding to the reduction of small and large CuO NPs, respectively^26^. For bimetallic 2Cu*x*Zn catalysts, the addition of Zn (up to 2 wt% Zn) promotes the reducibility of catalysts, as evidenced by the increase in the peak intensity at ~300 °C relative to that of the peak at the higher temperature (Supplementary Table 6) and the latter reduction peak shifting from ~345 °C to 332–335 °C. This can be ascribed to highly dispersed Cu and/or Cu/ZnO interactions (where Zn acted as the electronic promoter)^26^. These findings indicate that the addition of Zn to the catalyst promotes Cu dispersion, enhances the Cu reducibility and forms Cu/ZnO interfacial sites, which enhances the methanol formation (Table 1). However, as noted with the activity data, there is an optimum loading of Zn. The H_2_-TPR shows that at 4 wt% Zn, the Cu may result in more extensive interactions between Cu and ZnO species together with the formation of aggregated low-activity ZnO NPs (Supplementary Fig. 15), leading to a higher reduction temperature (shifted to 309 °C and 356 °C, Fig. 2c) and diminished methanol yield (Fig. 1).

### The active sites responsible for methanol synthesis in NTP-catalytic CO_2_ hydrogenation

The variation in the Cu and/or Zn environments in the 2Cu and 2Cu*x*Zn catalysts following thermal reduction by H_2_ was probed by XAS. Cu in the as-synthesized 2Cu and 2Cu*x*Zn resembled CuO (Fig. 2d). Fitting the Cu *K*-edge extended X-ray absorption fine structure (EXAFS) data showed a first shell Cu–O component at 1.93 Å and a Cu–Cu component at 2.96 Å (Supplementary Fig. 19 and Supplementary Table 7), in good agreement with the structure of CuO^27–29^. After reduction, the X-ray absorption near edge structure (XANES) spectra of all Cu-based catalysts shifted to lower energies, signifying a partial reduction of the Cu (Fig. 2d). In line with the H_2_-TPR data, the addition of Zn increased the Cu reducibility up to 2 wt% Zn (that is, 2Cu < 2Cu1Zn < 2Cu2Zn) with the decreasing effect for 2Cu4Zn (Table 1). From the linear combination fitting (Supplementary Fig. 19), the largest fraction of Cu^0^ fraction was found in the 2Cu2Zn catalyst (Table 1), which also showed the highest coordination number in the first shell when fitting the EXAFS to metallic Cu; for example, the Cu–Cu coordination number was 3.59 for 2Cu versus 5.08 for 2Cu2Zn (Supplementary Table 7). Moreover, as well as a Cu–Cu path at 2.54 Å due to metallic copper, all the H_2_ treated samples require a Cu–O path to achieve a good fit, confirming some oxidic character remains. The distance of the Cu–O path (1.85 Å) is closer to that found in Cu_2_O than in CuO, although the residual oxidized Cu components are likely to be a mixture of Cu^+^ and Cu^2+^. Please see Supplementary Note 1 for further discussion on the difficulties of differentiating the two in partially reduced Cu samples.

In contrast with the Cu *K*-edge data, the Zn *K*-edge XANES for the 2Cu*x*Zn and 2Zn catalysts showed little change on reduction with all systems resembling Zn^2+^ (Supplementary Fig. 20). The observed Zn *K*-edge data did not map precisely to either the ZnO or Zn(II) hydrate reference materials, although shared some, albeit very muted, features with the ZnO. The lack of features is presumed to result from the absence of long-range ordering in the zinc oxide species, suggesting very small clusters of oxygen-coordinated zinc cations rather than a crystalline zinc oxide species. On increasing the Zn loading from 2% to 4%, Zn was found to more closely resemble the structure of ZnO from the Zn–Zn and Zn–O coordination numbers, indicating some level of ZnO aggregation, in line with the HAADF–STEM. Indeed, only in the 4 wt% Zn catalyst were reflections of ZnO observed, highlighting the high dispersion at lower concentrations with agglomeration occurring from 4 wt% (Supplementary Figs. 1 and 2 and Supplementary Table 1). Fitting the EXAFS data (Supplementary Table 8) and the wavelet transformation EXAFS oscillations (Supplementary Figs. 21 and 22) confirmed the Zn has only oxygen in the first shell, with no features consistent with a close Cu–Zn or Zn–Zn interaction. Therefore, Cu/Zn alloy formation is thought to be unlikely after H_2_ treatment for any of the bimetallic samples. This was further confirmed by in situ XPDF during thermal reduction (Supplementary Note 2). The contributions from Zn containing species were fit using a hexagonal (wurtzite) ZnO phase for the 2Cu2Zn, 2Cu4Zn and 2Zn catalysts before and after H_2_ reduction (Supplementary Table 9 and Supplementary Figs. 23–27). This phase was identified by powder X-ray diffraction in 2Cu4Zn and is, therefore, an appropriate model. For lower-loaded samples, where XAFS indicates Zn is more cationic in nature, coordinated to oxygen but without long-range ordering, this model can still be used to confirm the presence of Zn–O atomic pairs.

In situ DRIFTS of CO adsorption on the 2Cu, 2Cu*x*Zn and 2Zn catalysts following the 400 °C H_2_ treatment was conducted at 25 °C to examine the surface sites present. As shown in Fig. 2e, 2Zn did not adsorb CO, while 2Cu exhibited a broad band at ~2,110 cm^−1^, corresponding to CO adsorption on Cu^0^ (refs. ^30,31^). Comparatively, the 2Cu*x*Zn catalysts showed stronger IR bands following CO adsorption, which were deconvoluted into two bands at 2,110 cm^−1^ and 2,130 cm^−1^ and assigned to linearly bound CO on Cu^0^ (Cu^0^–CO) and Cu^+^ (Cu^+^–CO), respectively^30^. The higher wavenumber band at ~2,130 cm^−1^ reflects the reduced back donation of electron density from metallic Cu to the antibonding 2π* orbital of CO, thus could be related to the Cu/ZnO interface, which is coordinatively or electronically unsaturated due to the Cu–O–Zn interaction^30,32^. Supplementary Table 10 presents the percentage of Cu^+^–CO/(Cu^0^–CO + Cu^+^–CO), which is representative of the extent of the number of interfacial sites between Cu and ZnO, having the order: 2Cu4Zn (71.6%) > 2Cu2Zn (62.7%) > 2Cu1Zn (35.5%) > 2Cu (0%). However, this order does not correlate with the activity data in Fig. 1 and methanol STY (Supplementary Fig. 28), indicating that both reduced and oxidized Cu (from the Cu/ZnO interface) contribute to methanol formation and an appropriate balance between the two types of active site is needed to maximize the overall methanol formation. This balance is changed by the Zn loading and is optimal at 2 wt% Zn.

### Operando XAS study of the role of NTP and Cu/Zn synergy

**Fig. 3:**
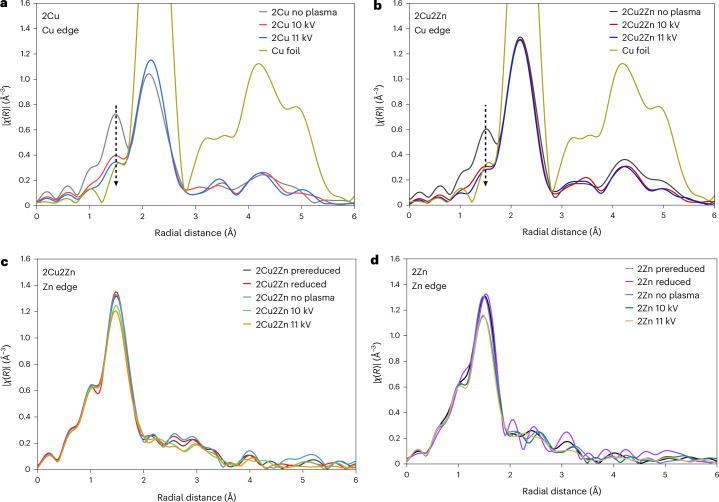
Influence of applied voltage on local Cu and Zn environments during CO_2_ hydrogenation. Fourier-transformed (FT)-EXAFS (R-space) spectra at the Cu and Zn *K*-edges recorded under steady-state NTP conditions. **a**, Cu *K*-edge FT-EXAFS of 2Cu. **b**, Cu *K*-edge FT-EXAFS of 2Cu2Zn. **c**, Zn *K*-edge FT-EXAFS of 2Cu2Zn. **d**, Zn *K*-edge FT-EXAFS of 2Zn recorded under steady-state NTP conditions. Experimental conditions: NTP at 10 kV and 11 kV (corresponding to 3.0 W and 4.4 W); gas feed 20%CO_2_/60%H_2_/Ar; total flow rate 20 ml min^−1^. |χ(R)| is the magnitude of the Fourier transform. Source data

Under steady-state conditions (at 10 kV and 11 kV, corresponding to 3.0 W and 4.5 W, with the reaction feed of H_2_/CO_2_ = 3 and Ar balance), a comparative operando XAS study of two selected catalysts, 2Cu and 2Cu2Zn, was conducted (Supplementary Note 3). The mass spectrometry profiles (Supplementary Figs. 29 and 30) showed that the 2Cu2Zn was more active than 2Cu for methanol formation under the NTP-XAS conditions, which was consistent with the activity data (Fig. 1). On ignition of the NTP, the Cu *K*-edge XAS spectra for both the 2Cu and 2Cu2Zn catalysts show that the Cu is more reduced compared with when the NTP was extinguished. This state closely resembled metallic Cu, especially at the higher applied voltages (Fig. 3 and Supplementary Figs. 31 and 32) with a decrease in the contribution from the Cu–O path in the radial distribution function (Supplementary Tables 11 and 12). This is further evidenced by the EXAFS (Supplementary Figs. 31a, 32a and 34) and XANES (Supplementary Figs. 31b and 32b) data. Comparing the 2Cu and 2Cu2Zn catalysts under NTP conditions, the EXAFS for the 2Cu2Zn at 11 kV could be fitted without a Cu–O path (Fig. 3b and Supplementary Table 12) while some Cu oxide was always found in 2Cu, further highlighting the improved reducibility of 2Cu2Zn over 2Cu. As found with the thermal reduction of the catalysts, the Zn environment showed little change irrespective of the reaction conditions remaining as an oxygen-coordinated Zn^2+^ species (Fig. 3c,d, Supplementary Figs. 33–36 and Supplementary Tables 13 and 14). The above findings showed that (1) during the NTP catalysis the plasma reduces the Cu species further and sustains the predominantly reduced Cu state regardless of the presence of Zn, and (2) the NTP does not affect the Zn environment and it remains in the Zn^2+^ form under all conditions.

During thermal catalysis, it has been observed that CO_2_ dissociation and formed H_2_O can oxidize or sinter the active sites causing deactivation^24,33,34^; however, little deactivation was observed during the NTP catalysis (Fig. 1c). To examine the role of CO_2_ dissociation under NTP conditions, transient XAS experiments, in which the gas feed was cycled between a CO_2_/Ar feed (labelled as the CO_2_ cycle) and a CO_2_/H_2_/Ar feed (H_2_:CO_2_ = 3; labelled as the H_2_ cycle), were performed. At 10 kV, on switching from the H_2_ cycle to the CO_2_ cycle, the Cu species in both 2Cu and 2Cu2Zn systems become oxidized (Supplementary Figs. 39a and 40a), due to the production of oxidizing species (for example O* species and O_2_) from NTP-induced CO_2_ dissociation^11,35^ (Supplementary Figs. 37 and 38). Switching back to the H_2_ cycle reversed this change (Supplementary Figs. 39 and 40). Moreover, continuously alternating between the H_2_ and CO_2_ cycle resulted in repeatable XAS data over seven cycles, demonstrating the resilience of both catalyst systems (Supplementary Figs. 39 and 40). For 2Cu, there was a subtle change after the first cycle, which is likely to be due to the initial system undergoing further reduction as a result of the NTP (Supplementary Fig. 39). On changing to an oxidizing environment (the CO_2_ cycle, cycle 3), the pre-edge of 2Cu shifts from 8,978 eV to a higher energy level of 8,980 eV (Supplementary Fig. 39a), indicating a small degree of oxidation. This is associated with a sharpening of the pre-edge feature at 8,981 eV, as seen in Cu_2_O, but to a lesser degree than found for the bulk material. In the reducing environment (the H_2_ cycle, cycles 1–7), two features contributed to the white line at 8,993 eV and 9,003 eV, which are present to a lesser degree than found for metallic Cu foil. However, in the CO_2_ cycle (cycles 1–7), these features are replaced by a single feature at 8,996 eV, again similar to the XANES of Cu_2_O. The same findings are also observed in the 2Cu2Zn system (Supplementary Fig. 40). Therefore, NTP plays a role in sustaining and regenerating the reduced state of Cu active sites for CO_2_ hydrogenation.

**Fig. 4:**
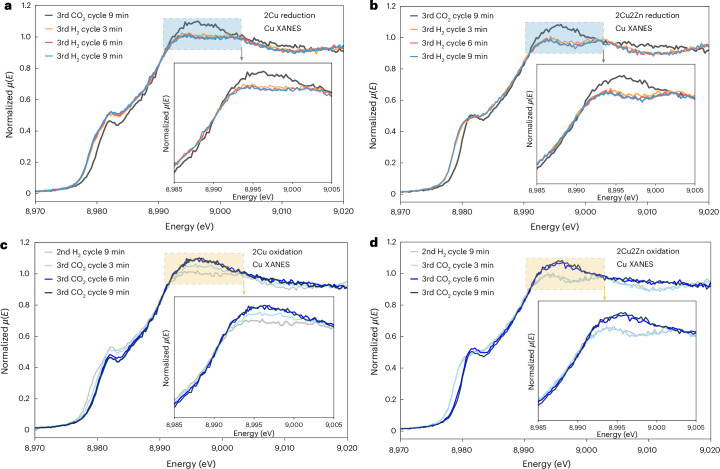
Time-resolved XANES spectra of 2Cu and 2Cu2Zn catalysts under NTP conditions. **a**,**b**, 2Cu (**a**) and 2Cu2Zn (**b**) during the transition from a CO_2_ atmosphere (CO_2_/Ar, CO_2_ cycle) to the reaction condition (CO_2_/H_2_/Ar, H_2_ cycle) at 10 kV NTP. **c**,**d**, 2Cu (**c**) and 2Cu2Zn (**d**) during the reverse transition from the reaction conditions (CO_2_/H_2_/Ar, H_2_ cycle) back to a CO_2_ atmosphere (CO_2_/Ar, CO_2_ cycle) at 10 kV NTP. Insets show the magnified region of the dashed rectangles. Experimental conditions: NTP at 10 kV, 3.0 W; gas feed: 20%CO_2_/Ar or 20%CO_2_/60%H_2_/Ar; total flow rate 20 ml min^−1^. *μ*(*E*) is the linear X-ray absorption coefficient. Source data

A notable difference between the 2Cu and 2Cu2Zn systems was the relative rates of oxidation and reduction when exposed to the different gaseous environments under NTP conditions (Fig. 4). The XAS spectra were collected every 3 minutes and usually the three spectra were averaged to create a single spectrum; however, examining each individual spectrum introduces a temporal resolution to the analysis. Switching from the CO_2_ cycle to the H_2_ cycle, both the 2Cu and 2Cu2Zn catalysts transform to a predominantly reduced Cu state within the first 3 minutes, with little further variation over the subsequent 6 minutes (Fig. 4a,b). In contrast, a difference in the time dependence of the XAS evolution was found on switching from H_2_ cycle to the CO_2_ cycle, as shown in Fig. 4c,d. Following the switch, the 2Cu catalyst started to be oxidized in the first 3 minutes and the spectra continued to change up to 6 minutes (Fig. 4c) when the oxidation was completed. Conversely, for 2Cu2Zn, the initial predominantly reduced spectra (H_2_ cycle 9 minutes; Fig. 4d) is similar to the first spectrum collected after 3 minutes under oxidizing CO_2_ conditions (CO_2_ cycle 3 minutes; Fig. 4d), showing that 2Cu2Zn was oxidized more slowly than 2Cu. It is only during the 6-minute scan (CO_2_ cycle 6 minutes; Fig. 4d) that the 2Cu2Zn spectrum changes with no further change observed between the 6-minute and 9-minute scans. The findings here show that the 2Cu2Zn catalyst is more resistant to oxidation than the 2Cu catalyst. This may be correlated with the higher stability found for the bimetallic system. Mass spectrometry data collected during the cycling showed stable methanol formation over the 2Cu2Zn catalyst whereas a decrease was observed for the 2Cu catalyst (Supplementary Figs. 37 and 38). This resistance to re-oxidation of the 2Cu*x*Zn catalysts may be due to the oxygen vacancy on ZnO phase that probably adsorb relevant oxidative species (such as O_2_ and O*) preferentially from gas discharge^36,37^. The presence of oxygen vacancy (on ZnO) is evidenced by electron paramagnetic resonance (EPR) characterization (Supplementary Fig. 41), showing a prominent signal at *g* = 2.004 (attributed to the unpaired electrons deeply trapped in oxygen vacancies). The operando XAS study under NTP catalysis provide direct evidence of the synergy between NTP and Cu or Zn species, explaining the good performance of 2Cu2Zn in methanol synthesis via NTP-catalytic CO_2_ hydrogenation.

### The mechanism for methanol synthesis under NTP conditions

Previously reported studies have proposed that methanol synthesis from CO_2_ hydrogenation (under thermal activation) proceeds either via CO produced from the reverse water–gas shift reaction (RWGS) followed by CO hydrogenation to methanol (the RWGS + CO-hydro pathway)^38^, or through direct CO_2_ hydrogenation to formate or hydrocarboxyl intermediates, followed by further hydrogenation to methanol (the formate and hydrocarboxyl pathway, respectively)^39,40^. Herein, operando DRIFTS-MS experiments were conducted over 2Cu and 2Cu2Zn catalysts during NTP catalysis to elucidate the specific pathway(s) and the influence of the Cu/ZnO interface for methanol formation (Supplementary Note 4). The mass spectrometry profile (Supplementary Figs. 42 and 43) shows that 2Cu and 2Cu2Zn were inactive for CO_2_ hydrogenation without NTP. However, on plasma ignition, there was an immediate appearance of CO, methanol and CH_4_ signals in the mass spectrometry profiles, together with a decrease in CO_2_ signal. The corresponding DRIFTS spectra (band assignment for relevant surface species is listed in Supplementary Table 15) show that on exposure to the reaction gas mixture (2%CO_2_/6%H_2_/Ar), both 2Cu and 2CuZn show the formation of bidentate carbonate due to CO_2_ adsorption on the catalyst surface before plasma ignition (Supplementary Fig. 44) and were similar with the ZSM-5 support^41^ (Supplementary Figs. 44 and 45). On plasma ignition over the 2Cu catalyst (Supplementary Fig. 46), weakly adsorbed CO species on Cu^0^ sites were observed (small peaks at 2,117 cm^−1^ and 1,860 cm^−1^)^30^. In addition, the band intensity of the formate species (HCOO*) at 1,587 cm^−1^ and 1,405–1,440 cm^−1^ (refs. ^42,43^) gradually increased with reaction time (Supplementary Fig. 46b). Further, the evolution of surface species as a function of time showed that the formation rate of formate species is similar with the rate of methanol formation (Supplementary Fig. 47), confirming that formate species is the active intermediates. Once the plasma was extinguished, the formate species disappeared within 5 minutes (Supplementary Fig. 46d). Hence, for the monometallic 2Cu catalyst, the formate pathway (on metallic Cu^0^ sites) is probably dominant for methanol synthesis under NTP conditions.

**Fig. 5:**
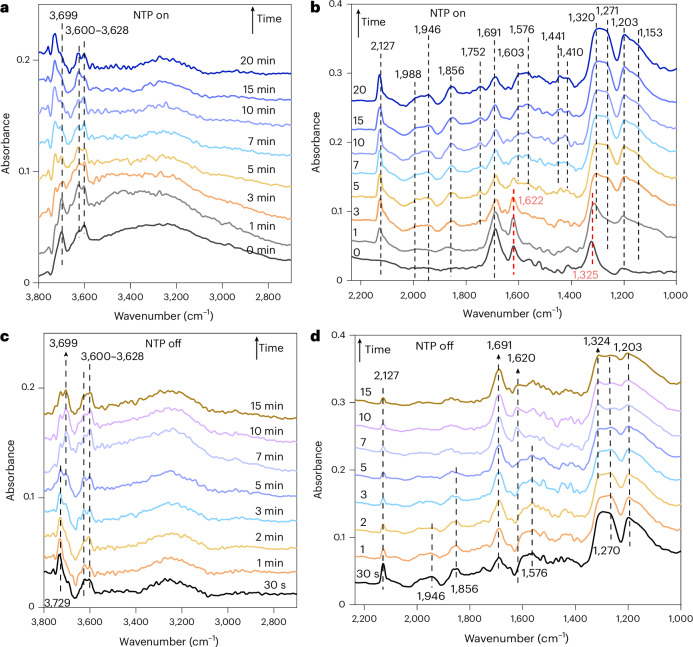
Temporal dynamics of operando DRIFTS analysis for NTP-catalytic CO_2_ hydrogenation over the 2Cu2Zn catalyst. **a**,**b**, Temporal evolution of DRIFTS spectra between 2,700 and 3,800 cm^−1^ (**a**) and between 1,000 and 2,250 cm^−1^ (**b**) under NTP-on conditions. **c**,**d**, Temporal evolution of DRIFTS spectra between 2,700 and 3,800 cm^−1^ (**c**) and between 1,000 and 2,250 cm^−1^ (**d**) after NTP is switched off (experimental conditions: 2% CO_2_ + 6% H_2_ in Ar balance; plasma applied at: 5.0 kV, 27.0 kHz). Source data

**Fig. 6:**
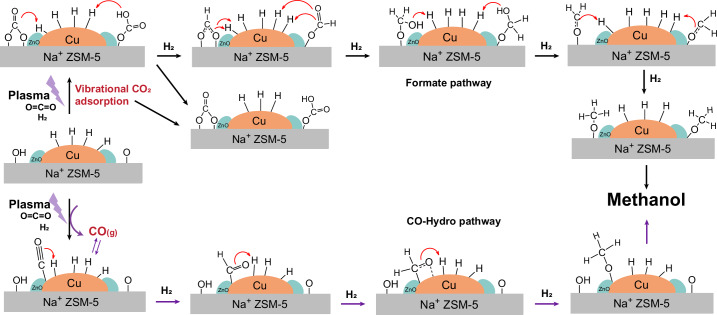
Proposed reaction mechanism for methanol synthesis over the 2Cu2Zn catalyst under NTP conditions. Schematic illustration of the dual-pathway mechanism for CO_2_ hydrogenation to methanol over the 2Cu2Zn catalyst under NTP activation. The mechanism involves two cooperative routes: the formate pathway on metallic Cu sites (black arrows) and the CO hydrogenation pathway on interfacial Cu/ZnO sites (purple arrows).

Conversely, on plasma ignition (Fig. 5a,b), clear bands associated with CO adsorption on the 2Cu2Zn catalyst at 2,127 cm^−1^, 1,946–1,988 cm^−1^ and 1,856 cm^−1^ were observed (Fig. 5b), which may suggest that the interfacial Cu/ZnO sites can stabilize CO* intermediates^38^. As also found over the 2Cu catalyst, formate species at 1,410−1,440 cm^−1^ and 1,576–1,603 cm^−1^ were observed over 2Cu2Zn (Fig. 5b). Along with CO species, the bands at ~1,691 cm^−1^ on 2Cu2Zn decreased rapidly within 1 min of the plasma being ignited due to the reaction of bidentate carbonates with active H species. Bands in this region then increased gradually with time, which is consistent with the formation of formyl species (HCO*) formed via the hydrogenation of the strongly adsorbed CO species on the Cu/ZnO sites. The time evolution of the surface species as a function of time over 2Cu2Zn showed that the increased rates of formyl and formate species were similar to that of the methanol formation rate over the first 15 minutes. Thereafter, both species continued to increase, probably due to the accumulation on the support surface, for example, with methanol formation remaining constant, confirming that both species contribute to methanol formation (Supplementary Fig. 48). When the NTP was extinguished (Fig. 5c,d), the band intensity of the CO species diminished quickly and the formate species decreased within 5 minutes of the change. In contrast, the band at 1,691 cm^−1^ grew due to CO_2_ re-adsorption. Similarly, the transient cycling experiments in which the feed was switched between H_2_/CO_2_/Ar and H_2_/Ar (Supplementary Fig. 49) showed the same dynamic of formyl species and CO species, confirming the CO-hydro pathway (likely to be associated with the Cu/ZnO sites) for the NTP catalysis over 2Cu2Zn (Fig. 6). In this case, the formate species showed a slower increase and/or decrease rate than the formyl species. Although the rates of change are different, both species still changed with the cycling, which is consistent with the proposed two routes to form methanol via the formyl and formate species^44,45^. Accordingly, the operando data above show that Zn incorporation encouraged additional pathways for methanol formation under the NTP conditions compared with the cases using 2Cu, explaining the improved selectivity and/or yield to methanol (Fig. 1a,b). The Cu/ZnO interface stabilized the adsorbed CO and its subsequent hydrogenation to methanol (via formyl intermediates, CO-hydro pathway) in addition to the formate pathway observed on the metallic Cu sites over the 2Cu catalyst (Fig. 6).

According to the literature, strategies to improve thermal-catalytic performance typically involves enhancing water tolerance and CO_2_ adsorption by introducing basic sites and/or oxygen vacancies^46,47^, promoting H_2_ activation and spillover through incorporation of noble metal promoters^48^, and increasing Cu dispersion or forming bimetallic interfaces or alloys that act as active sites^49,50^. For NTP catalysis, some design principles overlap (for example, strong CO_2_ adsorption and high dispersion of active metals remain advantageous^51^), but the key difference is that reactive species are not generated solely on the heated catalyst surface. Instead, the plasma produces short-lived intermediates in the gas phase (such as vibrationally excited CO_2_, CO, H and O) either through direct dissociation or secondary reactions of excited species. These plasma-generated intermediates can participate directly in surface reactions via different pathways with lower energy barriers (for example, Eley–Rideal pathways^8^). In particular, plasma-produced CO and H atoms can adsorb on Cu-based catalysts, enabling alternative CO-hydro pathways for methanol synthesis, as demonstrated in this work.

NTP catalysis also benefits from the interplay between plasma discharges and porous materials. Monte Carlo simulations show that micro-discharges near porous catalyst surfaces locally intensify the electric field, thereby increasing the density of plasma-induced species^52^. This synergy contributes to higher activity and selectivity by strengthening local electric fields within the packed ZSM-5-supported Cu catalyst to generate reactive species; by incorporating plasma-produced gas-phase intermediates (CO, H, vibrationally excited CO_2_) in methanol formation, which enables new reaction pathways beyond those in thermal catalysis, and by operating at milder conditions, which mitigates deactivation pathways such as Cu sintering or oxidation. In addition, electric field effects and surface charging can modulate CO_2_ adsorption by shifting antibonding orbitals towards the valence band, thereby stabilizing intermediates and lowering activation barriers^53^. These features help explain why catalysts that are ineffective under thermal conditions can exhibit superior performance in plasma (Supplementary Fig. 9). Nonetheless, plasma-induced species are intrinsically short-lived (nanoseconds for electronically excited molecules and microseconds for radicals^15^) making their use highly dependent on rapid transport to the catalyst surface before quenching or gas-phase recombination^15,54^. This highlights the importance of catalyst architecture: while zeolite-encapsulated Cu/ZnO_*x*_ catalysts exhibit high methanol productivity under thermal conditions^55^, they perform poorly in plasma due to restricted access of reactive plasma-induced species^56,57^. Moreover, plasma discharges cannot form in pores smaller than the Debye length (>2 μm)^52^, underscoring the need for hierarchical mesopores or macropores to facilitate plasma–catalyst synergy. Hence, whereas effective thermal catalysts are optimized for stable active sites, strong CO_2_ adsorption and efficient H_2_ activation, effective NTP catalysts must also possess hierarchical porosity to facilitate plasma–catalyst synergy and species transport, present highly accessible and dispersed surface-active sites (for example, single atoms or nanoclusters) to maximize use of short-lived species, and exploit plasma-induced electronic effects that promote CO_2_ adsorption and activation.

## Discussion

This study presents the rational design of an NTP catalyst for methanol synthesis based on the classic bimetallic Cu/Zn system and ZSM-5 zeolite. Compared with conventional CZA catalysts (~70 wt% Cu and 20 wt% Zn), the low metal loadings used here result in a high metal surface area and uniform dispersion of active sites, enabling direct elucidation of the synergy between plasma and metal active sites via in situ and operando techniques. The importance of Cu/ZnO interactions is evidenced both by catalytic performance and detailed characterization. The introduction of Zn improves Cu dispersion and reducibility, forms interfacial Cu/ZnO active sites, and limits Cu re-oxidation, collectively enhancing stability and increasing surface CO coverage, which is a key factor in methanol formation. Beyond the conventional surface formate pathway on metallic Cu, Cu/ZnO interfaces enable an additional CO-hydro pathway, accounting for the observed high methanol selectivity and yield in the bimetallic system. NTP provides alternative pathways for initial CO formation compared with thermal activation. Under NTP, CO can be generated through gas-phase CO_2_ dissociation, bypassing the high-temperature requirement for CO formation via RWGS on the catalyst surface. Previous density functional theory studies have proposed that plasma-induced CO species can promote methanol formation either through the reaction of surface-adsorbed CO with plasma-generated H radicals to form formyl species (HCO*) via Langmuir–Hinshelwood and/or Eley–Rideal mechanisms, or through the interaction of vibrationally activated CO_2_ with gas-phase H radicals to form HCOO* without energy barriers^8,58^. Our operando DRIFTS observations confirm these mechanisms, as vibrationally activated CO_2_, surface CO, formate and formyl species were observed on 2Cu2Zn surface as active intermediates under NTP conditions.

NTP also plays a critical role in maintaining the reduced state of Cu for CO_2_ hydrogenation, as revealed by operando XAS. The predominantly reduced Cu phase is rapidly regenerated within ~3 minutes, independent of Zn presence, highlighting the advantage of NTP over conventional thermal activation. This underscores the use of advanced in situ and operando techniques in identifying active sites, reaction pathways, and the interplay between plasma and catalyst. A key mechanistic insight is that the active Cu state must be balanced between partially oxidized and reduced states, while the bimetallic catalyst retains oxidized Zn. No evidence of Cu/Zn alloy formation was observed when using ZSM-5 as the carrier under NTP activation. Crucially, NTP enables low-temperature, low-pressure methanol production, in contrast to the elevated conditions required for thermal catalysis. These mild conditions, coupled with plasma-induced activation, sustain catalyst activity over time by preventing Cu oxidation and facilitating desorption of reaction intermediates, thereby maintaining the catalytic cycle. Overall, our findings reveal the mechanistic origins of the performance differences between monometallic Cu and bimetallic Cu/Zn catalysts in the NTP-catalytic CO_2_ hydrogenation. The unique role of plasma in sustaining catalytic activity was revealed, providing a framework for rational design and optimization of NTP catalysts, which benefit the promising route towards electrified chemical processes for sustainable fuels and chemicals production.

## Methods

### Chemicals

Chemicals used here tetrapropylammonium hydroxide (TPAOH, 40 wt% in H_2_O, Merck), colloidal silica (LUDOX AS-40, 40 wt% suspension in H_2_O, aluminium hydroxide (Al(OH)_3_, 95 + %, Wako), sodium hydroxide (50 wt% in H_2_O, Wako), copper(II) acetate monohydrate (>99.0%, Wako), zinc acetate dihydrate (99.9%, Wako) and commercial CuO/ZnO/Al_2_O_3_ catalyst (Alfa Aesar, catalogue no. 45776, lot no. I06Z036).

### Synthesis of ZSM-5 zeolite

Zeolite ZSM-5 was prepared using a method reported elsewhere with a molar ratio of 25 Na_2_O:Al_2_O_3_:300 SiO_2_:20 TPAOH:2,300 H_2_O^59^. Typically, Al(OH)_3_ (0.055 g) was added to a mixture of 3.406 g of TPAOH (40 wt%) and 1.336 g of NaOH (50 wt% in water) followed by stirring for 5 min. Then 15.0 g of colloidal silica was slowly added to the mixture, and the final mixture was stirred for 2 h at room temperature. The mixture was then transferred to a Teflon-lined stainless-steel autoclaves and heated at 150 °C at its autogenous pressure under static conditions for 5 h. The solid was then recovered by centrifugation, washed extensively with distilled water and dried at 80 °C overnight. The as-made materials were then calcined at 550 °C for 5 h in air to obtain the calcined ZSM-5 zeolites.

### Supported Cu, Zn and Cu–Zn catalysts on ZSM-5 zeolites

The supported Cu, Zn and Cu/Zn catalysts (with the theoretical loading of 2.0 wt% (Cu) and 1.0–4.0 wt% (Zn)) on the ZSM-5 zeolites above were prepared by impregnation. Typically, 1.0 g of calcined ZSM-5 was suspended in an aqueous solution of copper acetate monohydrate (0.062 g) and/or zinc acetate dihydrate (the concentration of the precursor solution depended on the metal loading). The suspension was stirred at 300 rpm for 1 h at room temperature and dried at 80 °C overnight. The obtained solid was calcined in a muffle furnace at 550 °C for 5 h. The resulting samples are denoted as 2Cu, 2Zn and 2Cu*x*Zn, respectively, where *x* refers to the theoretical Zn loading.

### Characterization

The crystalline properties of the materials were characterized by powder X-ray diffraction using a PANalytical X’Pert Pro diffractometer with a nickel absorber (0.02 mm, *K*_*ß*_ = 1.3923 Å) and Cu *K*_*α*1_ radiation at 40 kV, 40 mA with a scanning rate of 2° min^−1^ and a step size of 0.03° s^−1^. The morphology of the materials was investigated using a TESCAN Mira 3 high-resolution field scanning electron microscope at an accelerating voltage of 20.0 kV. HAADF–STEM and elemental mapping (EDX) were carried out using an FEI Talos F200A equipped with a X-FEG electron source, operated at 200 kV. EDX was collected using the FEI Super-X Quad silicon drift detector with a total collection angle of ≈0.9 sr. The dwell time is 12.5 μs and the beam current is 230 pA. The beam convergence angle is 10.5 mrad and the inner collection angle range is 40 mrad. The sample was dispersed in the ethanol solution and dropped on the Au grid. The high visibility low background double-tilt holder with alumina spacer and molybdenum clip was used to collect data. The average particle size and standard deviation was calculated by counting 450–500 NPs from HAADF–STEM images.

N_2_ physisorption analysis was conducted at −196 °C using a Micromeritics 3Flex Surface Characterization Analyser. Before the measurements, the samples (~100 mg) were degassed at 250 °C under vacuum overnight. The Brunauer–Emmett–Teller method was used to determine the specific surface area, and the specific total pore volume and micropore volume were obtained from the N_2_ adsorption branches of isotherms using the Barret–Joyner–Halenda method^60^.

The actual metal loading amounts were determined using inductively coupled plasma optical emission spectrometry (PQ 9000 Elite system). Before analysis, the sample (~20 mg) was dissolved in concentrated sulfuric acid (5 ml) and concentrated nitric acid (5 ml) by microwave digestion (ETHOS UP microwave digester).

H_2_-TPR was carried out using Quantachrome ChemBet Pulsar equipped with a thermal conductivity detector. Typically, ~30 mg of the sample was loaded in a quartz tube reactor and then pretreated with Ar at 200 °C for 1 h to remove the adsorbed water. After cooling to room temperature, a gaseous mixture of 5% H_2_ in Ar was introduced into the reactor (at 50 ml min^−1^) and the system temperature was increased to 800 °C at 10 °C min^−1^.

The dispersion of Cu was determined by N_2_O pulse chemisorption using Hiden HPR-20 mass spectrometer. Typically, 70 mg of the catalyst underwent a reductive treatment at 400 °C for 1 h under 20% H_2_/Ar. The H_2_ treated catalyst was then cooled to 200 °C and purge with Ar for 30 min to remove the H_2_ from catalyst. The N_2_O pulse was performed with 0.05% N_2_O/Ar at 200 °C until completely oxidization of the surface Cu atoms. The metal dispersion estimation was based on the assumption of a hemispherical geometry, assuming a N_2_O/Cu surface stoichiometry of 2.0.

The EPR data were collected with a Bruker EMX Plus Spectrometer operating at 9.4 GHz. Field corrections were applied using Bruker Strong pitch (*g* = 2.0028) as a reference sample. Simulations used EasySpyn v.6.0 software within MATLAB.

### In situ CO-DRIFTS

CO-DRIFTS was conducted to study the chemical state of Cu species and the interactions between Cu and Zn. The catalysts were first undergoing a pretreatment in 20% H_2_/Ar at 400 °C for 1 h. The H_2_ treated catalyst was then cooled to room temperature in an Ar atmosphere and purged using Ar for 30 min to clean the catalyst surface. The feed gas of 5% CO/Ar (50 ml min^−1^) was subsequently introduced to the system for 20 min at room temperature to saturate the catalyst surface by CO adsorption, where then the feed gas was switched to Ar (20 ml min^−1^) for spectrum collection. The IR spectra were collected on a Bruker Vertex 70v Fourier transform IR spectrometer and the OPUS software every 30 s (resolution of 4 cm^−1^), and each spectrum was the average of 32 scans.

### NTP catalysis

NTP catalysis was conducted at atmospheric pressure and room temperature using a bespoke DBD (6 mm outer diameter × 4 mm inner diameter) flow reactor (Supplementary Fig. 3). The DBD reactor consisted of a pair of coaxial quartz cylinders (inner (6 mm outer diameter × 4 mm inner diameter)) and outer quartz tubes (17 mm outer diameter × 15 mm inner diameter) in which a tungsten rod (0.75 mm outer diameter) was placed in the centre of inner quartz tube as the high-voltage electrode, with aluminium foil wrapped around inner quartz tube serving as the ground electrode. The circulating transformer oil was pumped into the jacket between the inner and outer cylinder to remove the heat produced by plasma and maintain the bulk DBD reactor temperature at ~15–20 °C. The cooled reactor system provided a mechanism to remove the heat generated. The discharge length and gap of the DBD reactor were 50 mm and 1.6 mm, respectively. The electrical parameters of NTP were monitored using a digital oscilloscope (Tektronix, MDO3022) via a high-voltage probe (Tektronix, P6015). The discharge power was calculated by using a typical Q-U Lissajous method (Supplementary Note 5 and Supplementary Fig. 50). Typically, ~310 mg catalysts (pelletized with particle size of 355–500 μm) were treated at 400 °C for 1 h under 20% H_2_/Ar (50 ml min^−1^). After reduction, the catalysts were quickly loaded in the discharge zone between two quartz wool plugs to minimize the re-oxidation by air exposure. Before reaction, the catalyst underwent a reductive pretreated in situ by plasma at specific input energy of 13.8 J ml^−1^ for 20 min using 50% H_2_–Ar as the discharge gas (50 ml min^−1^) to minimize the re-oxidation. In CO_2_ hydrogenation, the gas mixture of H_2_ and CO_2_ (molar ratio of 3:1) was fed into the DBD plasma reactor with the total flow rate of 40 ml min^−1^. The power was varied from 11.5 W to 16 W, corresponding to specific input energy of 17.25 J ml^−1^ to 24.00 J ml^−1^ using a constant frequency of 22.5 kHz. The outlet gas composition and methanol was analysed by a two-channel online gas chromatography (PerkinElmer, Clarus 590 model) equipped with HayeSep DB column, a thermal conductivity detector and a flame ionization detector. A propylene–Ar gas with constant concentration (5,000 ppm) and flow rate of 20 ml min^−1^ was introduced into the gas chromatographer and used as the external standard for calculations. The reaction temperature in the plasma discharge area was obtained by measuring the temperature of circulating oil temperature in this area (that is ~15 °C (inlet) to 20 °C (outlet)). For each measurement, three samples of products were taken and analysed under steady-state conditions. Average values of conversion and product selectivity were calculated from such triplicate measurements and error bars represent the standard deviation. The methanol was calculated according to the peak area from online gas chromatography by using the methanol vapour–Ar (10,000 ppm) standard gas as calibration. Control experiments, involving the NTP-only system with no catalyst present (that is, gas-phase reactions only) and NTP-assisted CO_2_ hydrogenation over the bare zeolite supports, were performed under the same conditions. For the commercial CZA catalyst, 500 mg of catalyst was treated at 250 °C under 20% H_2_/Ar (50 ml min^−1^) for 1 h before being assessed under the NTP conditions.

The CO_2_ conversion, gas product selectivity (of CO and CH_4_) and methanol were calculated using the following equations.

1 $${X}_{{{\rm{CO}}}_{2}}=\frac{{{\rm{CO}}}_{2,{\rm{in}}}/{n}_{{\rm{standard}}}-{{\rm{CO}}}_{2,{\rm{out}}}/{n}_{{\rm{standard}}}}{{{\rm{CO}}}_{2,{\rm{in}}}/{n}_{{\rm{standard}}}}\times 100{\rm{ \% }}$$

2 $${S}_{{\rm{CO}}}=\frac{{{\rm{CO}}}_{{\rm{out}}}/{n}_{{\rm{standard}}}}{{{\rm{CO}}}_{2,{\rm{in}}}/{n}_{{\rm{standard}}}-{{\rm{CO}}}_{2,{\rm{out}}}/{n}_{{\rm{standard}}}}\times 100{\rm{ \% }}$$

3 $${S}_{{{\rm{CH}}}_{4}}=\frac{{{\rm{CH}}}_{4,{\rm{out}}}/{n}_{{\rm{standard}}}}{{\rm{CO}}_{2,{\rm{in}}}/{n}_{{\rm{standard}}}-{{\rm{CO}}}_{2,{\rm{out}}}/{n}_{{\rm{standard}}}}\times 100{\rm{ \% }}$$

4 $${S}_{{{\rm{CH}}}_{3}{\rm{OH}}}=\frac{{{\rm{CH}}}_{3}{{\rm{OH}}}_{{\rm{out}}}/{n}_{{\rm{standard}}}}{{{\rm{CO}}}_{2,{\rm{in}}}/{n}_{{\rm{standard}}}-{{\rm{CO}}}_{2,{\rm{out}}}/{n}_{{\rm{standard}}}}\times 100{\rm{ \% }}$$

where *X* is the CO_2_ conversion, *S* is the product selectivity and *n* is the molar of external standard. In addition, the liquid products were collected and analysed by gas chromatography–mass spectrometer (Agilent GC 7890B, DB-WAX column) to confirm that methanol is the major product with an insignificant presence of ethanol (Supplementary Fig. 7 and Supplementary Table 3), and temperature-programmed oxidation and thermogravimetric analysis on spent 2Cu and 2Cu2Zn catalysts were conducted to investigate the coke formation and carbon balance (Supplementary Fig. 8).

For comparison, all the catalysts were evaluated under the thermal-catalytic CO_2_ hydrogenation in a flow reactor by using similar feed conditions at 2.0 MPa. Roughly 300 mg catalysts were treated at 400 °C for 1 h under 20% H_2_/Ar (50 ml min^−1^). Subsequently, the reactor was pressurized to 2.0 MPa under mixture gases of H_2_–CO_2_. The catalytic performances were evaluated at 50 °C, 100 °C, 200 °C and 300 °C. The stability of the catalysts was assessed at 300 °C with 42 h.

For temperature-dependent NTP catalysis, the transformer oil was kept at various temperatures of 10 °C, 50 °C, 80 °C and 125 °C and combined with NTP discharge at 14 W with a constant frequency of 22.5 kHz. Other conditions, such as catalyst mass, reduction process and feed gas were the same with the above NTP catalysis. For each measurement, three samples of products were taken and analysed under steady-state conditions.

### XANES

Cu and Zn *K*-edge XANES measurements were collected on the B18 beamline at Diamond Light Source, through a standard access proposal (nos. SP29271-7 and SP36241-1). Data were collected in fluorescence mode using a quick-scanning EXAFS setup, with an energy resolution of 0.27 eV, with fast scanning Si(111) crystal monochromator and a Pt mirror. Each scan took approximately 3 min. Unless stated otherwise, each dataset shown is merged from three scans. A home-designed DBD quartz capillary reactor was used for the experiments, in which a tungsten wire is placed in the centre of a quartz tube (3 mm outer diameter) as the high-voltage electrode and an aluminium foil wrapped around the quartz tube served as the ground electrode. About 40 mg of catalyst (particle size of 125–355 μm) was packed inside the quartz reactor between two quartz wool plugs. The temperature of reactor was kept at ~15 °C. This configuration only allows for measurements in fluorescence mode. Mass flow controllers were used to ensure the accurate delivery of gases into the DBD reactor. The reactor was heated with an air blower for the catalyst reduction process with a *K*-type thermocouple placed with the reactor to measure the actual temperature. A mass spectrometer connected to the system outlet was used to record the gas components from the NTP catalysis.

To investigate the variation in Cu and Zn chemical properties of the catalysts before the after thermal reduction, XAS spectra of the selected catalysts were collected. The prereduction spectra were collected at 30 °C, under a flow of 50 ml min^−1^ Ar and 2 ml min^−1^ Kr to record their initial state. The system was then heated from 30 °C to 400 °C linearly over 40 min and held at 400 °C for 30 min under reduction conditions with 4 ml min^−1^ H_2_, 14 ml min^−1^ Ar and 2 ml min^−1^ Kr. After that, the systems were cooled down to 30 °C, and the gases were switched back to 50 ml min^−1^ Ar and 2 ml min^−1^ Kr to collect the postreduction spectra.

For operando NTP-catalytic CO_2_ hydrogenation experiments under steady-state conditions, the reaction mixture (of 4 ml min^−1^ CO_2_, 12 ml min^−1^ H_2_, 2 ml min^−1^ Ar and 2 ml min^−1^ Kr) was introduced into the system with the NTP off for 10 min. Subsequently, the NTP was ignited and held at 10 kV for 30–60 min, allowing the spectra at 10 kV to be collected. The NTP voltage was then increased to 11 kV and held for a further 30–60 min, allowing the spectra at 11 kV to be collected.

For operando NTP-catalytic CO_2_ hydrogenation experiments under transient cycling conditions, the feed gases were alternated between the reaction condition of H_2_ + CO_2_ (that is, 4 ml min^−1^ CO_2_, 12 ml min^−1^ H_2_, 2 ml min^−1^ Ar and 2 ml min^−1^ Kr) and the oxidizing CO_2_ condition (that is, 4 ml min^−1^ CO_2_, 14 ml min^−1^ Ar and 2 ml min^−1^ Kr). The gas was alternated every 10 min, allowing 3 scans to be collected and 7 cycles of switches under each condition were collected.

Data were analysed using the Strawberry Demeter packages Athena, Artemis and Atoms^28^. For Cu, the amplitude factor (*S*_0_^2^) was determined as 0.82 from fitting the Cu foil reference. Cu spectra were then fitted using a Hanning window function in a 3.0 to 11.0 *k*-range and a 1.0 to 3.0 *R*-range, fitting simultaneously to *k*^1^, *k*^2^ and *k*^3^ weightings. For Zn, the amplitude factor (*S*_0_^2^) was determined as 0.95 from fitting the Zn Foil reference. Zn spectra were then fit using a Hanning window function in a 3.0 to 11.0 *k*-range and a 1.0 to 3.0 *R*-range, fitting simultaneously to *k*^1^, *k*^2^ and *k*^3^ weightings.

### In situ XPDF during thermal reduction

The X-ray total scattering data were collected on the I15-1 beamline at Diamond Light Source, through a standard access proposal (CY33381) using a wavelength of *λ* = 0.161669 Å. Samples were loaded into a 3 mm quartz capillary reactor between two quartz wool plugs with 20% H_2_/He (at a total flow rate of 10 ml min^−1^) flowing through the system. A hot air blower was used for the variable temperature experiments. Data were collected from 50 °C to 400 °C in 50 °C increments with a program-controlled heating rate of 10 °C min^−1^, where the system was then kept at 400 °C for 1 h to continuously collect the data every 10 min. Each data collection was 10 min in length.

The atomic PDF was generated by a Fourier transform of the X-ray diffraction data using PDFGetX3 software^61^, which includes various corrective measures such as finite *Q* range and atomic scattering factors. The *Q* range extended from 0 Å^−1^ to 20.7 Å^−1^, with a Qmaxinst value of 21.8 Å^−1^ used to calculate PDFs up to an *r* value of 50 Å. Real-space refinements were performed using TOPAS v.6^62^, with user-written macros implementing PDF real-space refinement on TOPAS^63^. Initially, peak broadening and dampening from instrument factors were refined using the Si standard, which were then fixed for sample PDF refinements. Data were collected for the empty capillary, then scaled and subtracted from the sample data. The ZSM-5 phase was fit for the ZSM-5 only support sample, and then the parameters were fixed and applied to the other samples to reduce the number of parameters and prevent correlation between refined parameters. The weight percentages, lattice parameters and atomic displacement parameters (*B*_eq_) for the different Cu and Zn phases were then refined for each sample before and after reduction treatments.

### Operando DRIFTS-MS

The home-made IR cell for the DRIFTS under NTP conditions has been described in detail elsewhere^64^. Before the DRIFTS experiments, the catalyst of ~35 mg was treated at 400 °C for 1 h with 20% H_2_/Ar flowing. The catalyst was then loaded into the IR cell quickly to reduce exposure to air and was pretreated in a 20% H_2_/Ar flow under the plasma (at 5.0 kV and 27.0 kHz) for 20 min. For the steady-state experiments, the gas mixture (of 2 vol.% CO_2_ and 6 vol.% H_2_ with Ar balance) was introduced into the IR cell to initiate the reaction. An Ar balance in the DRIFTS experiments was used to avoid the signal saturation of IR spectra and mass spectrometry signal. During the experiments, a constant peak voltage of 5.0 kV and pulse frequency of 27.0 kHz were used to avoid arcing between the electrodes. IR spectra were recorded every 30 s. For the transient experiments, with NTP on (at 5.0 kV and 27.0 kHz), the feed gas was switched between 2% CO_2_/6% H_2_/Ar and 6% H_2_/Ar. The gas was alternated every 3 min and 3 cycles of switches under each condition were collected. IR spectra were recorded every 10 s. The corresponding reactants and products were recorded using an online mass spectrometer (Hiden HPR-20) connected to the DRIFTS cell outlet.

## Supplementary information

Supplementary Information Supplementary Figs. 1–50, Tables 1–15 and Notes 1–5.

## Source data

Source Data Fig. 1 Numerical data.

Source Data Fig. 2 Source data for spectroscopy figure.

Source Data Fig. 3 Source data for spectroscopy figure.

Source Data Fig. 4 Source data for spectroscopy figure.

Source Data Fig. 5 Source data for spectroscopy figure.

## Data Availability

The data supporting the results of this study are available within the article and Supplementary Information and via figshare at 10.48420/27161757.v2 (ref. ^65^). Data are available from the corresponding authors upon request. Source data are provided with this paper.
