# Supplementary material for: Unveiling active sites and the cooperative role of non-thermal plasma and copper–zinc catalysts in the hydrogenation of CO2 to methanol
Source: Nat Catal. 2026 Feb 13;9(2):134–47. doi: 10.1038/s41929-025-01477-5 (PMC12945693; doi:10.1038/s41929-025-01477-5)
Supplement: Supplementary file 1 — Supplementary Figs. 1–50, Tables 1–15 and Notes 1–5. [file 41929_2025_1477_MOESM1_ESM.pdf]

nature catalysis

Supplementary information https://doi.org/10.1038/s41929-025-01477-5

Unveiling active sites and the cooperative role of non-thermal plasma and copper–zinc catalysts in the hydrogenation of CO_2 to methanol

In the format provided by the authors and unedited

Supplementary Information

Contents

Supplementary Figure 1. 3
Supplementary Table 1 4
Supplementary Figure 2. 5
Supplementary Table 2 6
Supplementary Figure 3. 6
Supplementary Figure 4. 7
Supplementary Figure 5. 8
Supplementary Figure 6. 8
Supplementary Figure 7. 9
Supplementary Table 3 9
Supplementary Figure 8 10
Supplementary Table 4 10
Supplementary Figure 9 11
Supplementary Figure 10 12
Supplementary Table 5 13
Supplementary Figure 11 14
Supplementary Figure 12 15
Supplementary Figure 13 16
Supplementary Figure 14 17
Supplementary Figure 15 18
Supplementary Figure 16 19
Supplementary Figure 17 20
Supplementary Figure 18 21
Supplementary Table 6 22
Supplementary Figure 19 23
Supplementary Note 1 24
Supplementary Table 7 24
Supplementary Figure 20 26
Supplementary Table 8 27
Supplementary Figure 21 29
Supplementary Figure 22 31
Supplementary Note 2 32

Supplementary Table 9 32
Supplementary Figure 23 33
Supplementary Figure 24 34
Supplementary Figure 25 35
Supplementary Figure 26 36
Supplementary Figure 27 37
Supplementary Figure 28 38
Supplementary Table 10 38
Supplementary Note 3 39
Supplementary Figure 29 40
Supplementary Figure 30 41
Supplementary Figure 31 42
Supplementary Table 11 43
Supplementary Figure 32 44
Supplementary Figure 33 45
Supplementary Table 13 45
Supplementary Table 14 46
Supplementary Figure 35 47
Supplementary Figure 36 48
Supplementary Figure 37 49
Supplementary Figure 38 50
Supplementary Figure 39 51
Supplementary Figure 40 52
Supplementary Figure 41 53
Supplementary Note 4 54
Supplementary Figure 42 55
Supplementary Figure 43 56
Supplementary Figure 44 57
Supplementary Figure 45 58
Supplementary Figure 46 59
Supplementary Figure 47 60
Supplementary Figure 48 60
Supplementary Figure 49 61
Supplementary Table 15 62
Supplementary Note 5 63
Supplementary Figure 50 63

As shown in Supplementary Fig. 1, the powder diffraction patterns are dominated by the reflections of the ZSM-5 framework, being identical for all the samples. ZnO phase was hardly observed by PXRD. For the catalyst with the highest loading of Zn (i.e., 2Cu4Zn), minor reflections of hcp ZnO were identified, whilst the intensity of relevant diffraction peaks in 2Cu2Zn was lower. The lattice parameters and crystallite size are presented in Supplementary Table 1. Regarding Zn migration, Zn may either migrate into the pores/channels of the ZSM-5 framework or be incorporated into its zeolitic framework, as reported previously on similar CuZn/ZSM-5 catalysts.[1, 2] In this study, regarding the ZnO migration into the pores/channels of ZSM-5, it is likely due to the measured high Zn dispersion. However, it is not possible to distinguish Zn-Al and Zn-Si distances from Zn-O or Zn-Zn with any certainty using EXAFS to demonstrate the close interaction of the Zn and the zeolite. Regarding the possibility of forming the Zn-substituted framework in the ZSM-5 zeolite, we consider it being unlikely since the calcination temperature at 550 °C is not sufficiently high resulting in changes to the framework substitution. According to the literature, hydrothermal or ion exchange methods are necessary to achieve isomorphous substitution.[1-3] Furthermore, as shown in Supplementary Table 1, lattice parameters obtained from powder diffraction data did not show any correlations between Zn loading and unit cell volume of ZSM-5 zeolite, indicating that Zn was not incorporated into the ZSM-5 framework.

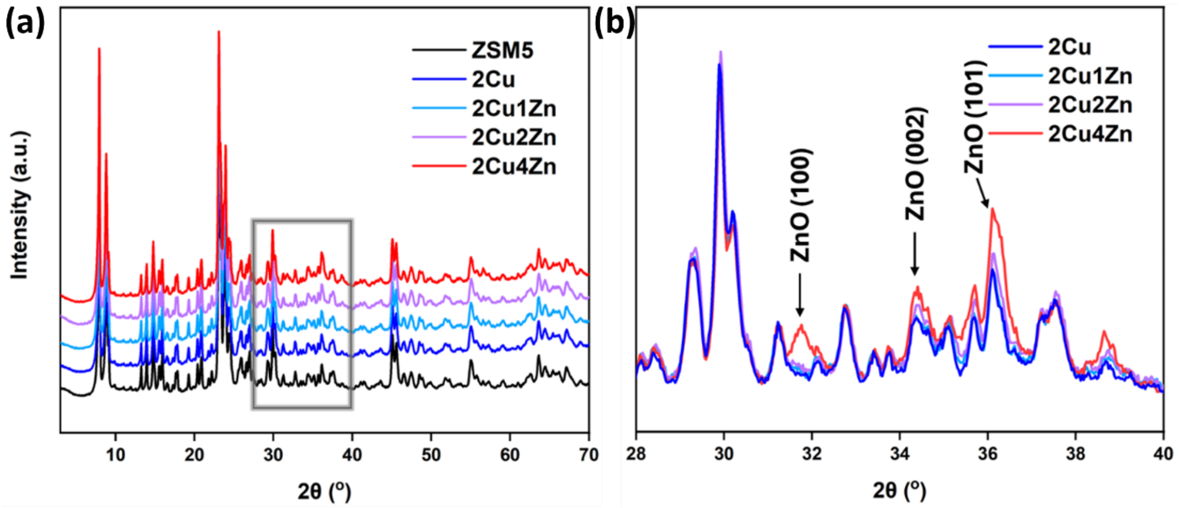

Supplementary Figure 1. PXRD patterns of the calcined catalysts measured at 1.5409Å of ZSM-5, with various Cu and Zn loadings. The grey box in (a) shows the region selected for magnification in (b), which indexes the ZnO reflections (space group P63 mc).

Supplementary Table 1. Calculated lattice parameters, volume, crystallite size and resulting weight of residuals (wR) from PXRD refinements.

Catalyst	Lattice parameter (Å)			α, β, γ (°)	Volume ($Å^3$)	Crystallite size (nm)	Weight of residual (%)
	‘a’	‘b’	‘c’				
ZSM-5	19.91(2)	20.12(2)	13.39(1)	90, 90.43(1)	5361(1)	6	10.4
2Cu/ZSM-5	19.92(2)	20.13(2)	13.40(1)	90, 90.50(1)	5372(1)	6	7.9
2Cu1Zn/ZSM-5	19.92(2)	20.13(2)	13.39(1)	90, 90.49(1)	5370(1)	6	7.9
2Cu2Zn/ZSM-5	19.91(2)	20.11(2)	13.38(1)	90, 90.47(1)	5358(1)	6	8.1
2Cu4Zn/ZSM-5:							
ZSM-5 phase	19.92(2)	20.13(2)	13.39(2)	90, 90.47(1)	5369(1)	6	9.0
ZnO phase	3.26(3)	/	5.21(5)	90, 90, 120	48(0)	3	
2Zn/ZSM-5	19.91(2)	20.10(1)	13.38(1)	90, 90.44(1)	5355(1)	6	8.4

The ZSM-5 phase is monoclinic (space group $P12_1/n\ 1$), and the ZnO phase is hexagonal (space group P63 mc). A ZnO phase was only fit to 2Cu4Zn/ZSM-5, being the only pattern with sufficient intensity from ZnO reflections to allow this. The background was refined using a chebyshev^{-1} function with 6 coefficients for 2Cu/ZSM-5 and then fixed to this for 2Cu1Zn, 2Cu2Zn and 2Cu4Zn. It was refined independently for the other samples.

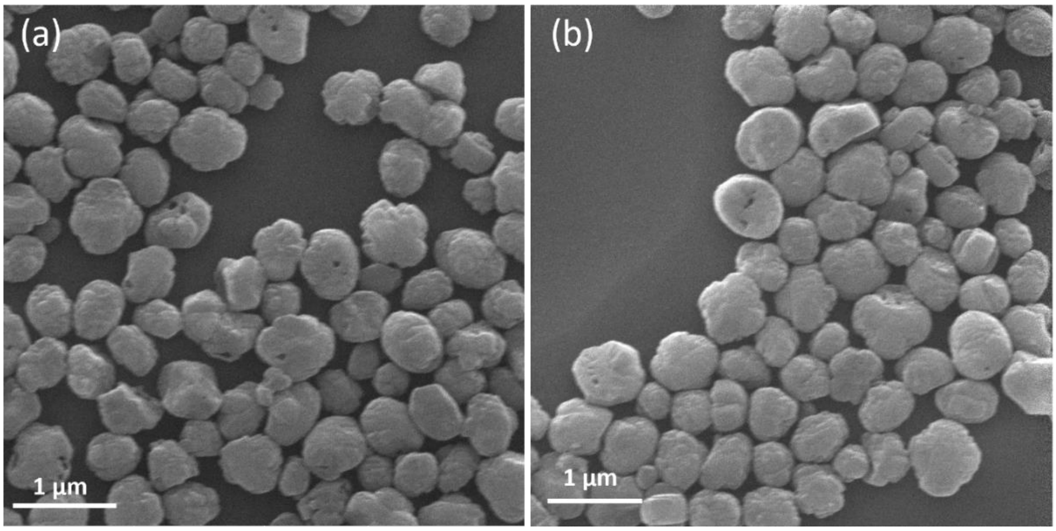

Supplementary Figure 2. SEM micrographs of (a) ZSM-5 and (b) 2Cu2Zn/ZSM-5.

Comparative SEM analysis of the bare ZSM-5 support and the 2Cu2Zn catalyst in Supplementary Fig. 2 shows the round-coffin morphology of ZSM-5 zeolite crystals with rough surfaces and the average particle sizes of 450–600 nm.

Supplementary Table 2. Specific surface area, pore volume and Cu and Zn content for the activated 2Cu and 2Cu*x*Zn/ZSM-5 catalysts.

Catalyst	Surface area (m^2 g^{-1})[a]	Pore volume (cm^3 g^{-1})		Cu loading (%)[d]	Zn loading (%)[d]
		V_{total}[b]	V_{micro}[c]		
Na-ZSM-5	374	0.256	0.159	-	-
2Cu	336	0.275	0.136	2.2	-
2Cu1Zn	336	0.258	0.133	2.3	0.9
2Cu2Zn	346	0.266	0.134	2.2	2.0
2Cu4Zn	322	0.250	0.130	2.1	3.4

[a] specific surface area calculated by the Brunauer–Emmett–Teller (BET) method.[4] [b] specific total pore volume estimated at $p/p^0 = 0.9929$. [c] micro-pore volume by the *t*-plot method. [d] Cu and Zn content determined by ICP-OES.

The actual Cu and Zn loading quantities were measured by ICP-OES, agreeing well with the nominal values (Supplementary Table 2). All the catalysts have similar specific surface areas (322−336 m^2 g^{-1}) and micro porosities (volume, 0.25–0.27 cm^3 g^{-1}), showing that metal incorporation did not significantly affect the porosity of the system.

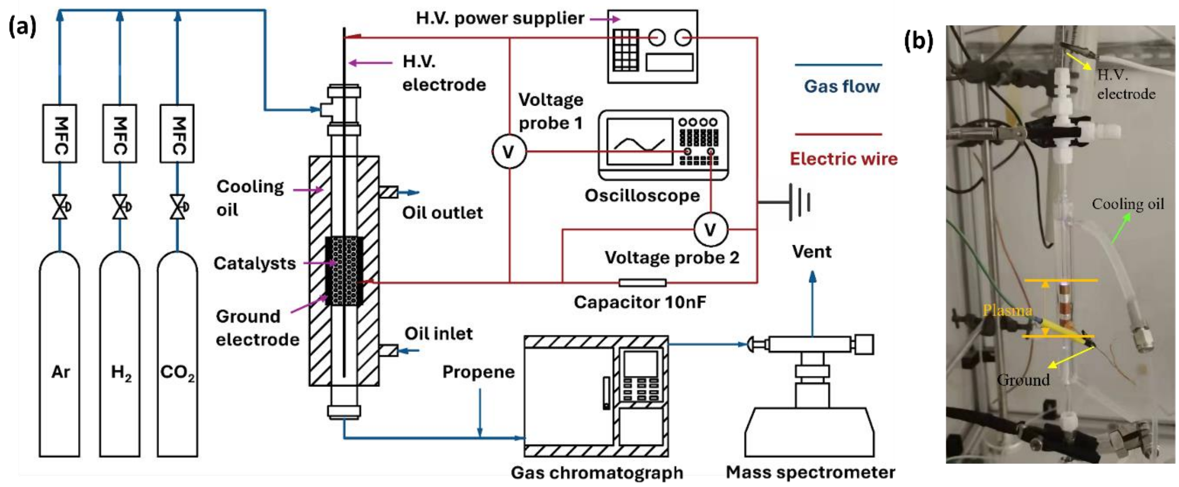

Supplementary Figure 3. (a) Schematic of the NTP system for CO_2 hydrogenation to methanol. (b) the image of jacketed NTP reactor with cooling oil circulating to regualte the bulk reactor temperature.

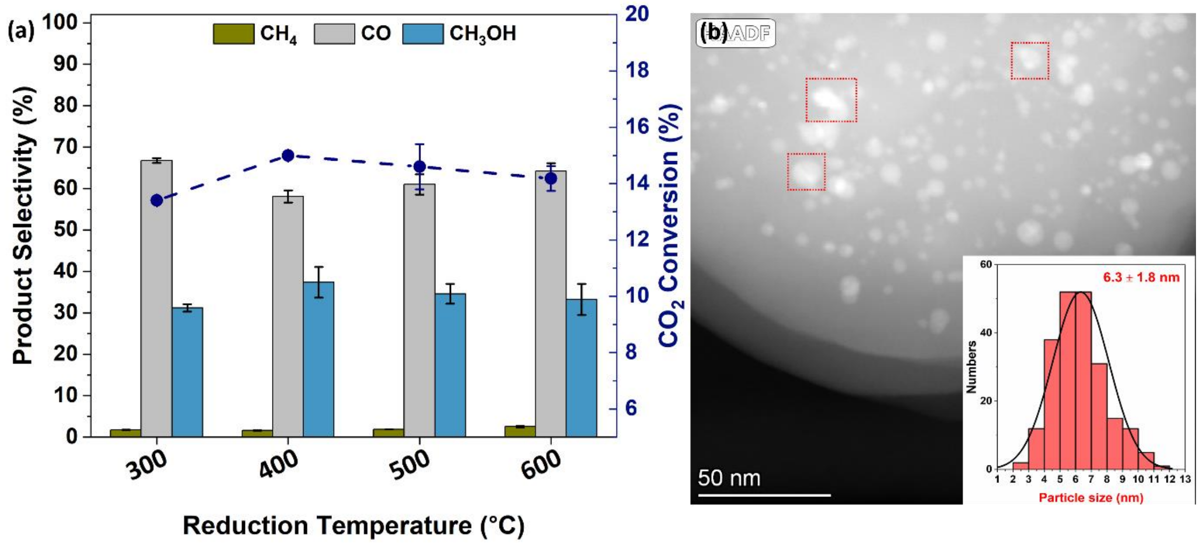

Supplementary Figure 4. (a) CO_2 conversion and product selectivity of the 2Cu2Zn/ZSM-5 catalyst reduced at 300, 400, 500 and 600 °C in NTP catalysis (Experimental conditions: at 14 W; gas feed = 25%CO_2/75%H_2, total flow rate = 40 ml min^{-1}; average values were calculated from measurements performed in technical triplicate; the error bars represent the standard deviation); (b) HAADF-STEM image and Cu particle size distribution of the 2Cu2Zn/ZSM-5 catalyst reduced at 600 °C (red dash rectangles to highlight the particle aggregation, inset: particle size distribution with the mean values and standard deviation obtained from the measurement of 250 particles).

The 2Cu2Zn catalyst was selected for preliminary study of the effect of the reduction temperature on the NTP catalysis. Thermal reduction may affect the state of metallic copper species, which will affect methanol formation in CO_2 hydrogenation.[5-7] As shown in Supplementary Fig. 4a, the 2Cu2Zn catalyst reduced at 400 °C demonstrated the highest CO_2 conversion and methanol selectivity, respectively. Comparison of the mean particle size measured from the sample reduced at 600 °C (6.3 ± 1.8 nm, Supplementary Fig. 4b) to that reduced at 400 °C (3.2 ± 0.7 nm, Supplementary Fig. 14f) reveals higher reduction temperatures leads to the formation of larger NPs, resulting in more CO being produced.

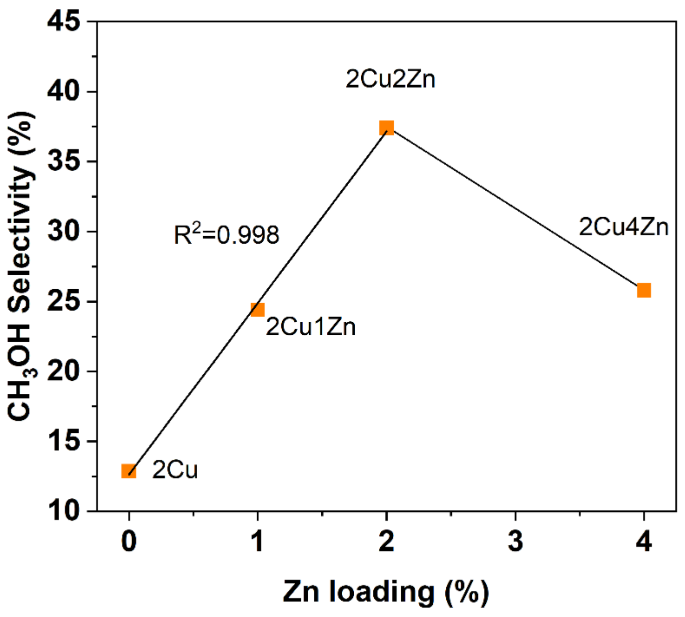

Supplementary Figure 5. The correlation between Zn loading and methanol selectivity of the NTP catalytic system.

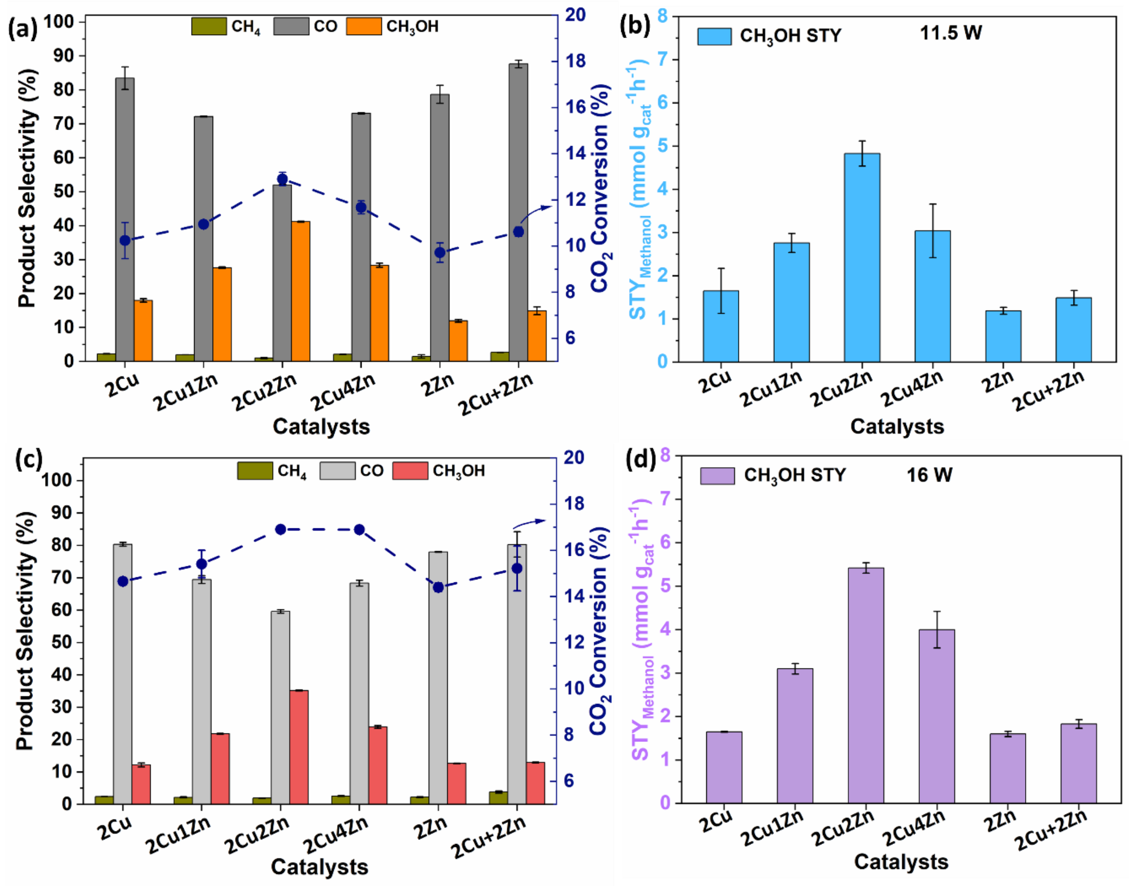

Supplementary Figure 6. CO_2 conversion, product selectivity and methanol space-time yield (STY) of the 2Cu*x*Zn catalysts (reduced at 400 °C) at different discharge powers: (a-b) 11.5 W and (c-d) 16 W. (Experimental conditions: gas feed = 25%CO_2/75%H_2, total flow rate = 40 ml min^{-1}; average values

were calculated from measurements performed in technical triplicate; the error bars represent the standard deviation).

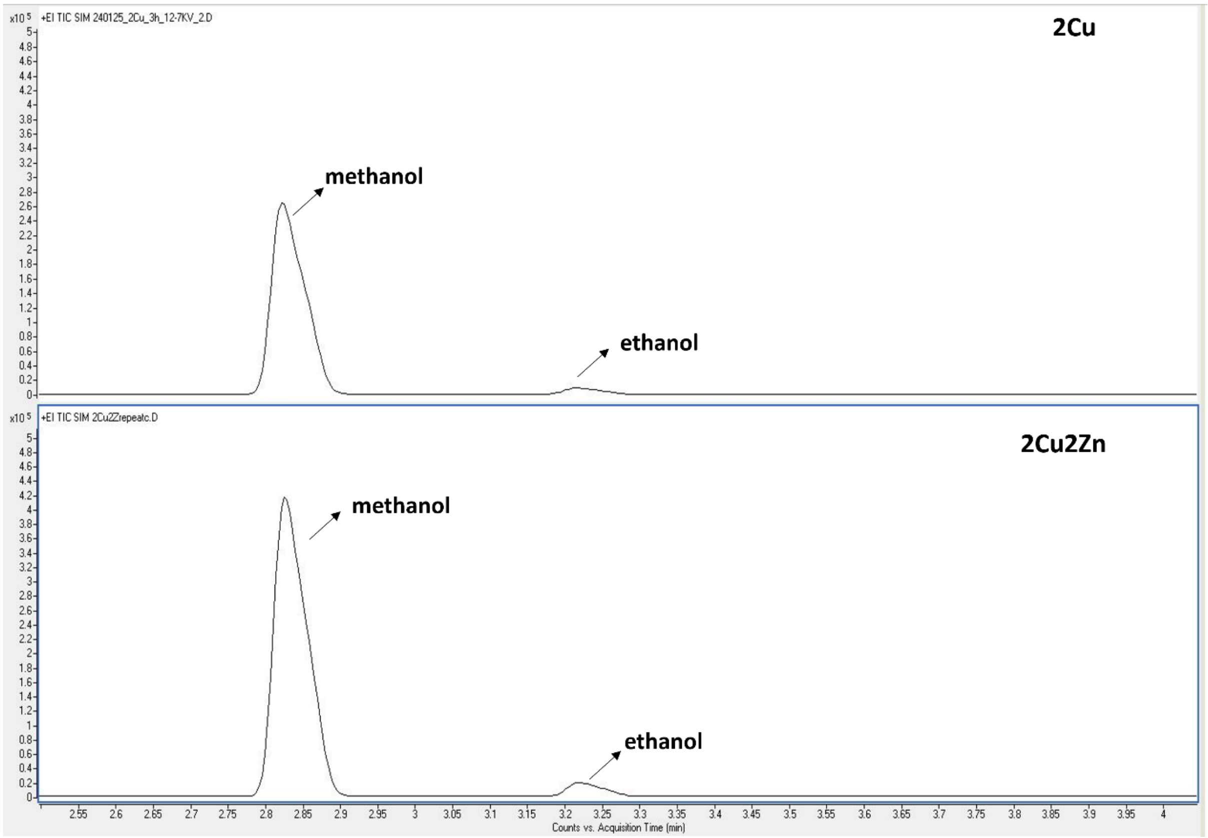

Supplementary Figure 7. Indentification of collected liquid products using gas chromatography-mass spectrum (GC-MS) after the plasma catalytic reaction. (Experimental conditions: NTP system: at 14 W; gas feed = 25%CO_2/75%H_2, total flow rate = 40 ml min^{-1})

Supplementary Table 3: The component of the liquid product from the plasma catalytic reaction (using the 2Cu and 2Cu2Zn catalyst) by GC-MS.

Catalyst	Methanol Concentration (vol%)	Ethanol Concentration (vol%)	Methanol/Ethanol
2Cu	0.543	0.017	31.9:1
2Cu2Zn	0.857	0.03	28.6:1

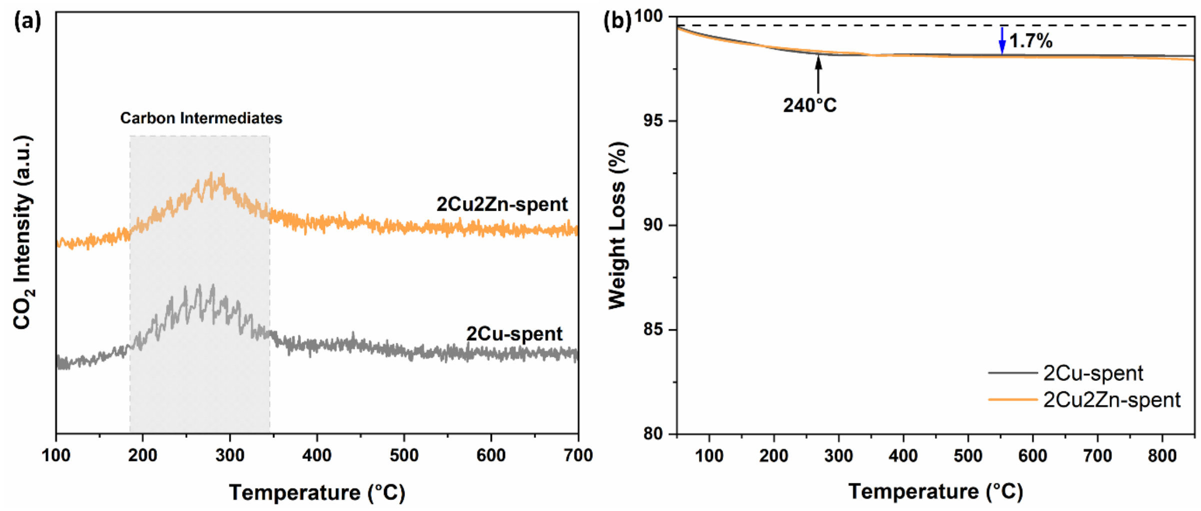

Supplementary Figure 8. (a) Temperature-programed oxidation (TPO) and (b) TGA of the used 2Cu and 2Cu2Zn catalyst after the plasma catalytic reactions.

As shown in Supplementary Fig. 7 and in Supplementary Table 3, methanol is the main liquid product with insignificant ethanol measured. The carbon balance was calculated to be ≥ 96% for all the NTP catalytic systems. The adsorbed carbon intermediates were evidenced by TPO, showing a peak at 250−280°C for the used 2Cu and 2Cu2Zn catalysts (Supplementary Fig. 8a). Also, TGA showed a weight loss of about 1.7% at 260°C (Supplementary Fig. 8b), which is consistent with the TPO results.

Supplementary Table 4: The catalytic performance of the 2Cu, 2Cu*x*Zn and 2Zn catalyst for methanol synthesis in the thermal catalytic system at different temperatures and 2 MPa.

Catalyst	50°C (CO_2 Con. & CH_3OH Sel. %)	100°C (CO_2 Con. & CH_3OH Sel. %)	300°C (CO_2 Con. %)	300°C (CH_3OH Sel. %)
2Cu	0	0	3.2	7.7
2Cu1Zn	0	0	3.9	3.4
2Cu2Zn	0	0	3.6	3.4
2Cu4Zn	0	0	4.0	2.5
2Zn	0	0	0.3	3.2

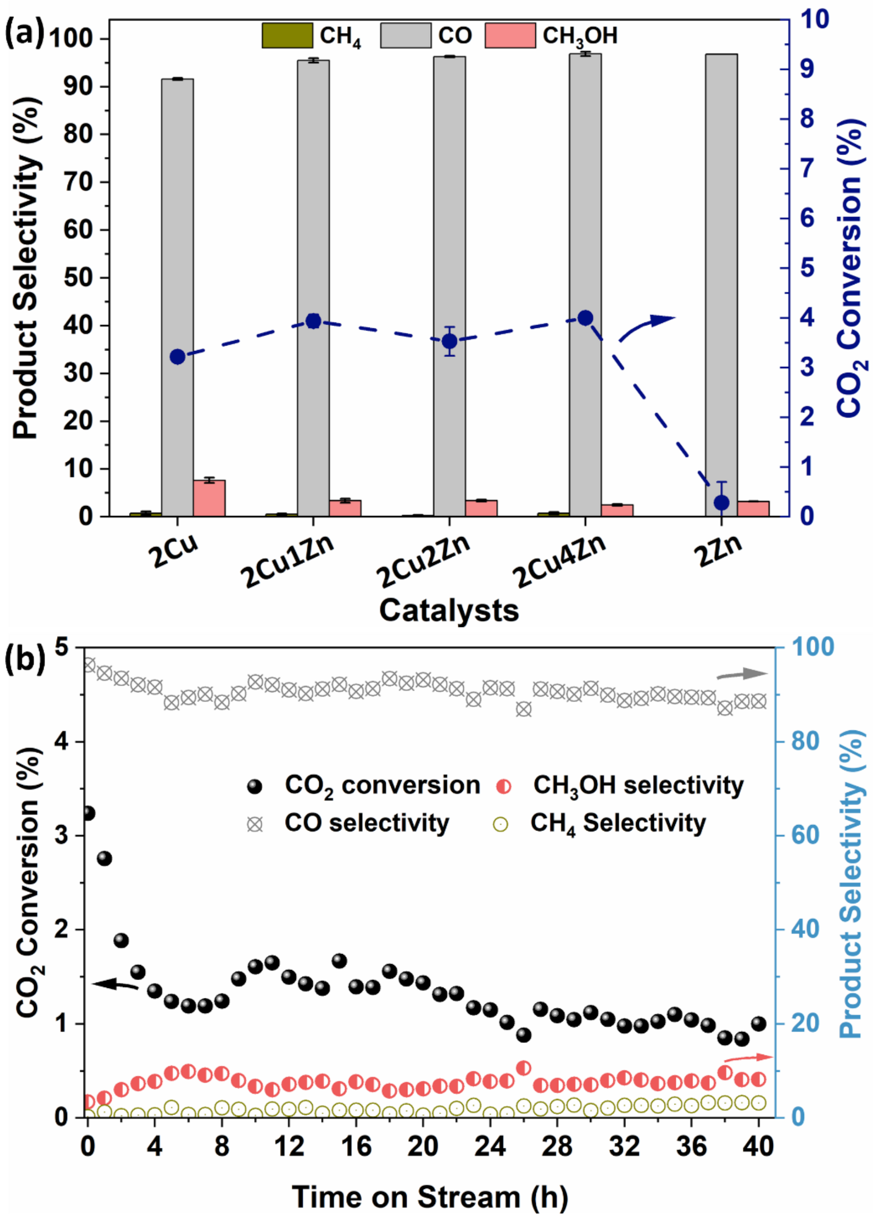

Supplementary Figure 9. (a) CO_2 conversion and product selectivity over the 2Cu*x*Zn catalysts under thermal conditions at 300 °C and 2.0 MPa (average values were calculated from measurements performed in technical triplicate; the error bars represent the standard deviation), and (b) Longevity test of 2Cu2Zn in the thermal catalytic CO_2 hydrogenation at 300 °C and 2.0 MPa.

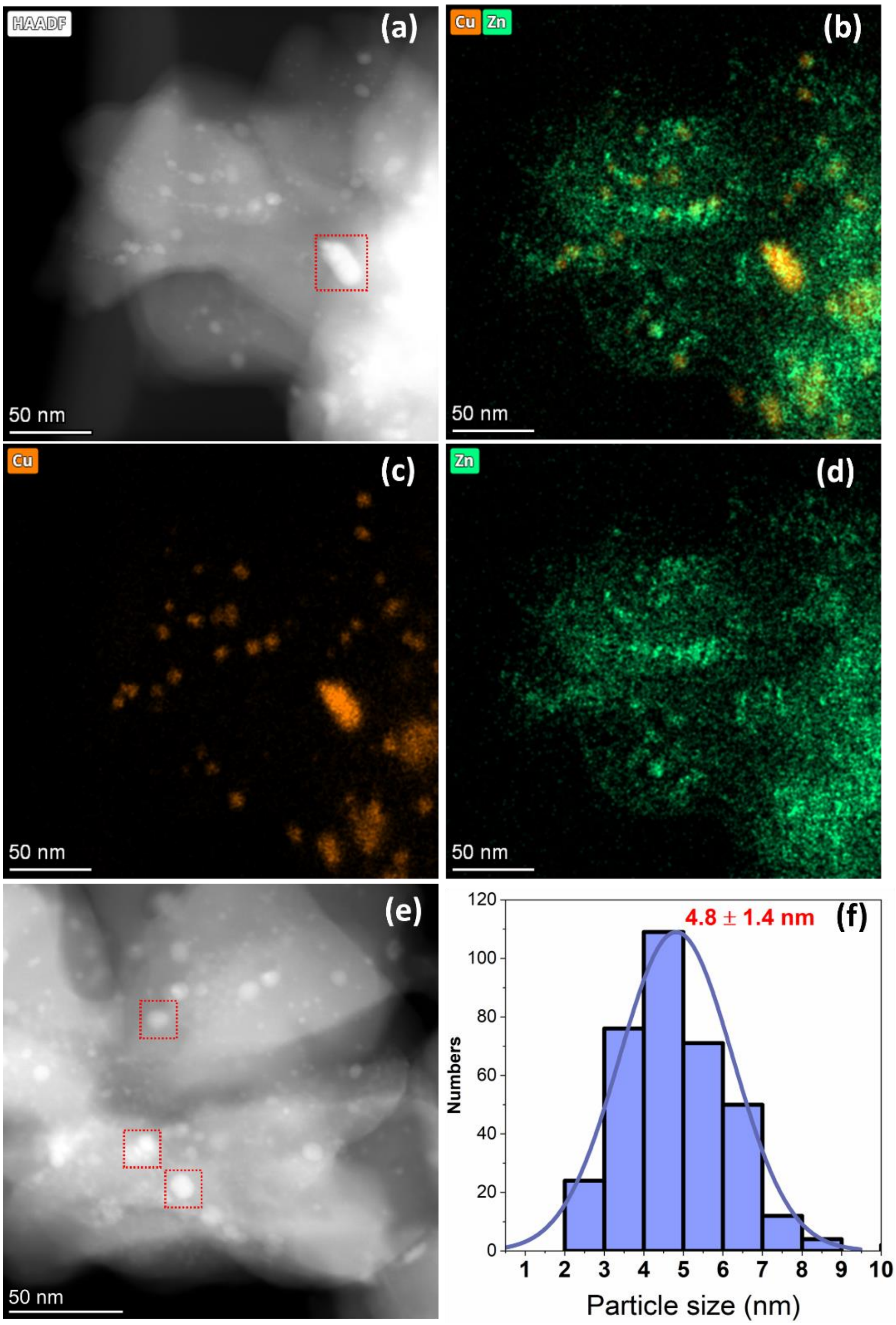

Supplementary Figure 10. (a, e) HAADF-STEM images, (b–d) corresponding EDS elemental maps and (f) Cu-ZnO particle size distribution of the used 2Cu2Zn catalyst after the 42-h stability experiment at 300 °C and 2 MPa (red dash rectangles to highlight the particle sintering, mean values and standard deviation are given on the histogram, the particle size distribution was calculated by the measurement of 350 nanoparticles).

Supplementary Table 5: Comparison of the catalytic performance between the NTP catalytic system (this work) and relevant conventional thermal catalytic systems.

Catalysts	Zn/(Cu +Zn)[a]	CO_2 con. (%)	MeOH sel. (%)	MeOH STY (mmol g_{cat}^{-1} h^{-1})	T, °C	P, MPa	GHSV mL g_{cat}^{-1} h^{-1}	Ref.
**Cu/Zn-O-Ce**	0.3	15.1	26.4	4.0	280	2.0	10,000	8
**CuZn-ZrO_2**	0.9	2.53	81	2.0	250	3.0	9000	9
**Cu/ZnO/SiO_2**	0.5	19.0	10.0	1.1	280	3.0	6000	10
**Cu/Zn/Al_2O_3**	0.3	24.6	67.1	8.8	260	5.0	5000	11
**Cu-ZnO-ZrO_2**	0.4	17.0	–	3.5	240	3.0	4500	12
**Cu/ZnO/CeO_2**	0.3	2.0	60	–	250	3.0	60,000	13
**Cu–Zn/SiO_2**	0.16	1.0	83	2.3	230	2.5	9000	14
**Cu-ZnO-$SrTiO_3$**	0.3	15.9	26.8	–	250	3.0	3000	15
**Cu/ZnO/ZrO_2**	0.5	21	47	4.3	250	3.0	3600	16
**This work**	**0.5**	**15**	**37.4**	**5.1**	**15**	**0.1**	**8000**	

[a]The ratio of Zn/(Cu+Zn) comes from the most effective catalysts in the literature and this work.

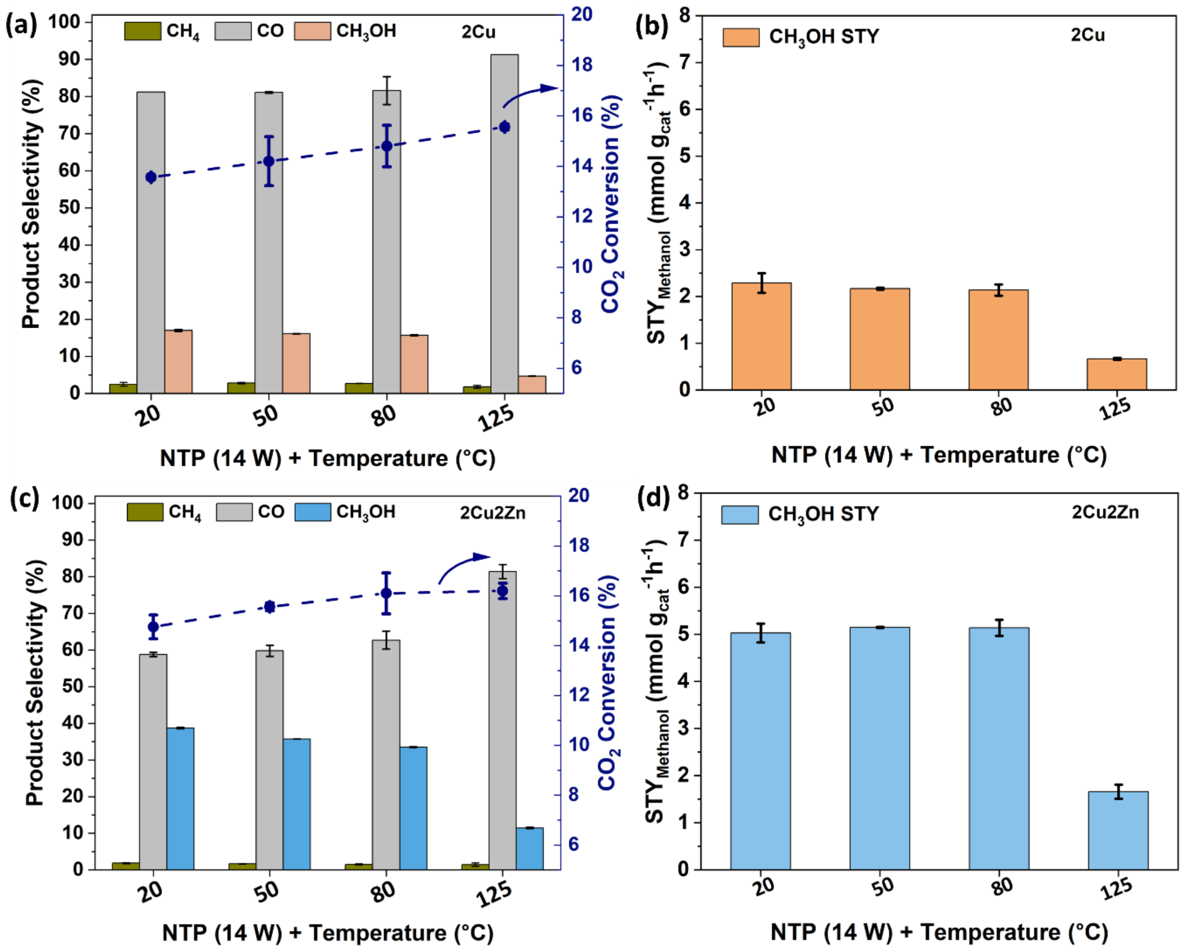

Supplementary Figure 11. CO_2 conversion, product selectivity and methanol space-time yield (STY) over (a–b) 2Cu and (c–d) 2Cu2Zn as a function of the bulk system temperature of the NTP catalytic system. (Experimental conditions: NTP power: 14 W; Heating temperature: 15–20 °C, 50 °C, 80 °C and 125 °C; gas feed = 25%CO_2/75%H_2, total flow rate = 40 ml min^{-1}). Average values in this Figure were calculated from measurements performed in technical triplicate; error bars represent the standard deviation.

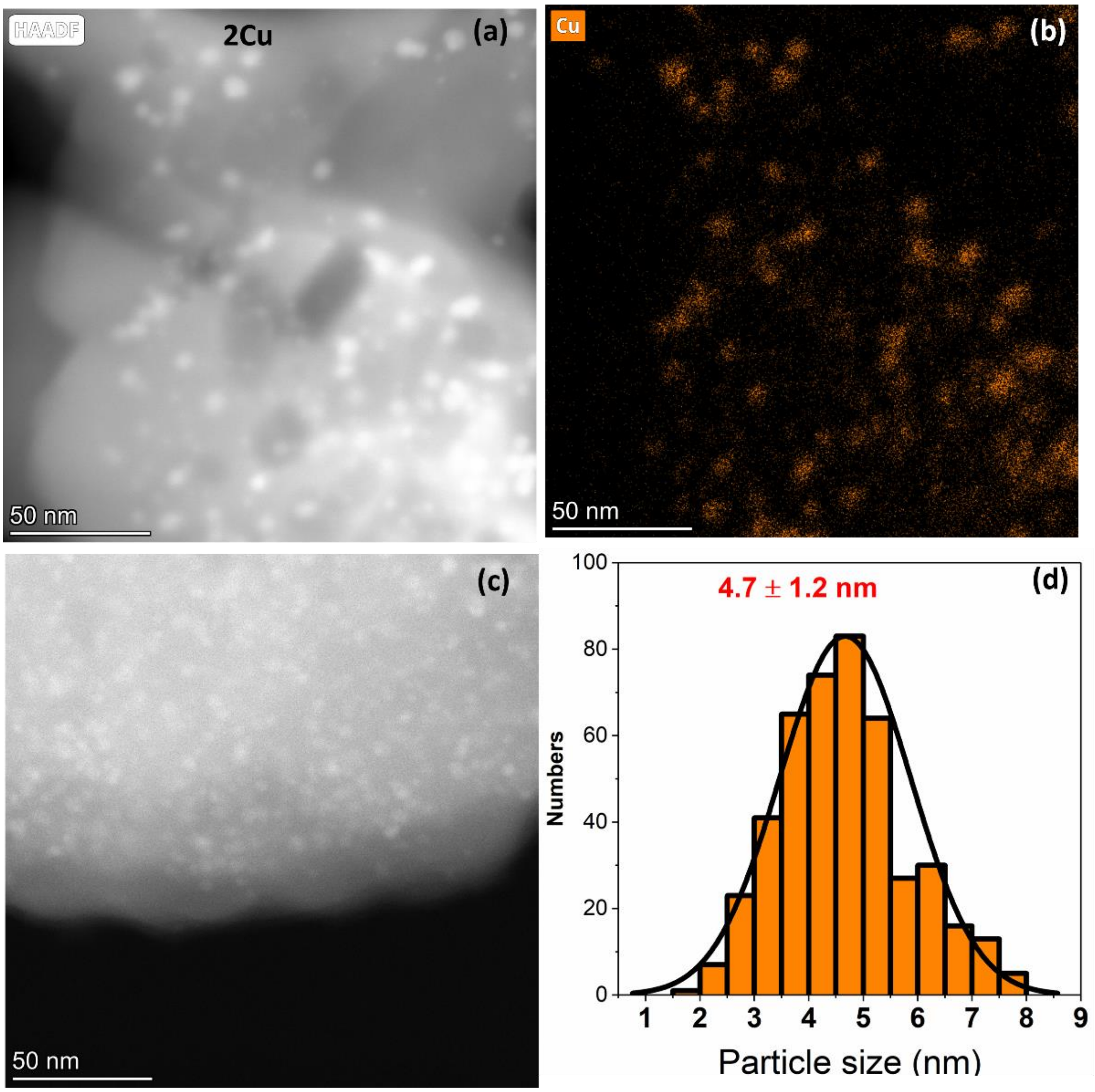

Supplementary Figure 12. (a, c) HAADF-STEM images, (b) corresponding EDS elemental maps and (d) Cu particle size distribution of the monometallic 2Cu catalyst reduced at 400 °C (mean and standard deviation values are given on the histogram, the particle size distribution was calculated by counting 450 nanoparticles).

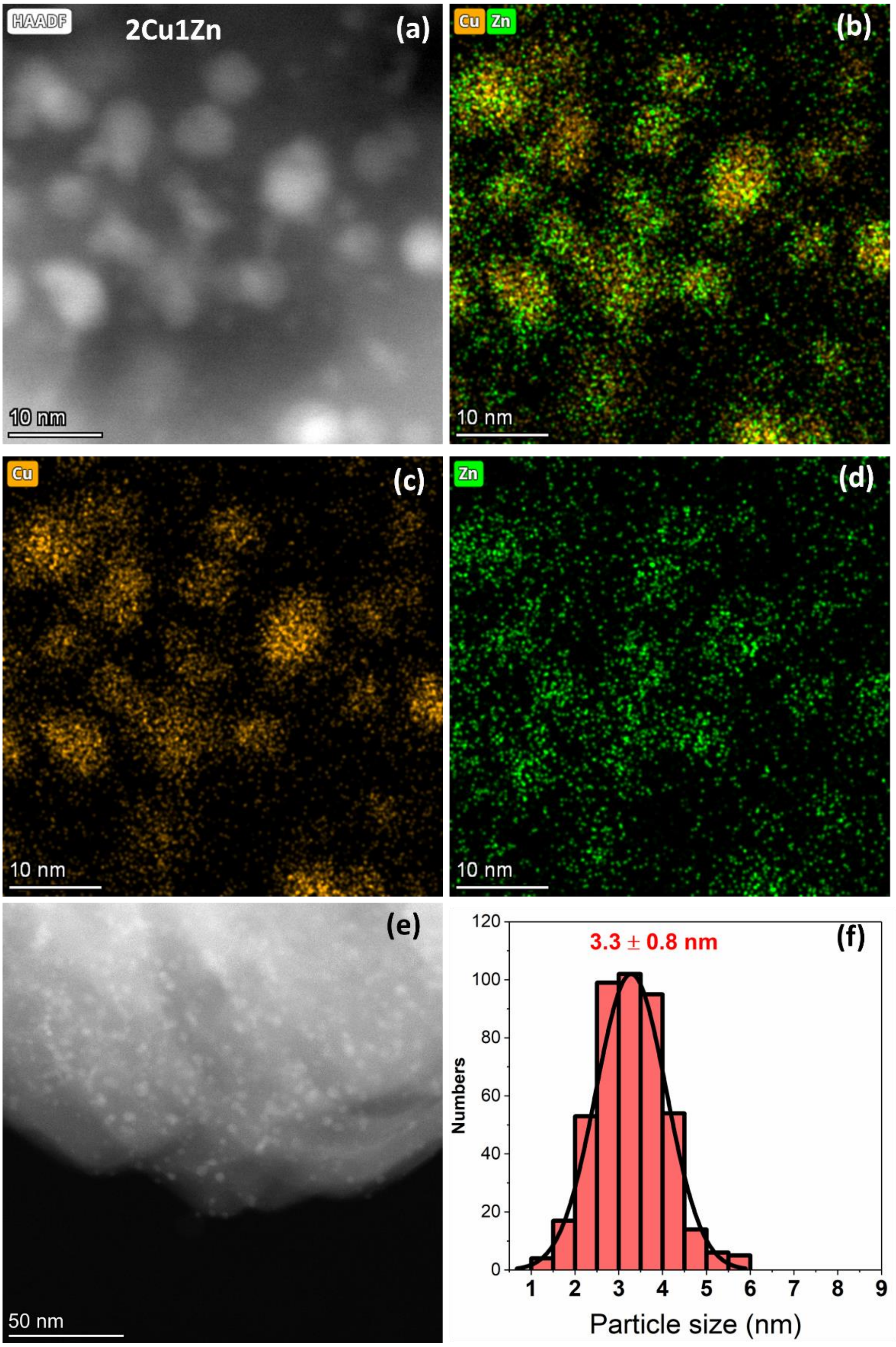

Supplementary Figure 13. (a, e) HAADF-STEM images, (b–d) corresponding EDS elemental maps and (f) Cu-ZnO particle size distribution of the 2Cu1Zn catalyst reduced at 400 °C (mean and standard deviation values are given on the histogram, the particle size distribution was calculated by counting 450 nanoparticles).

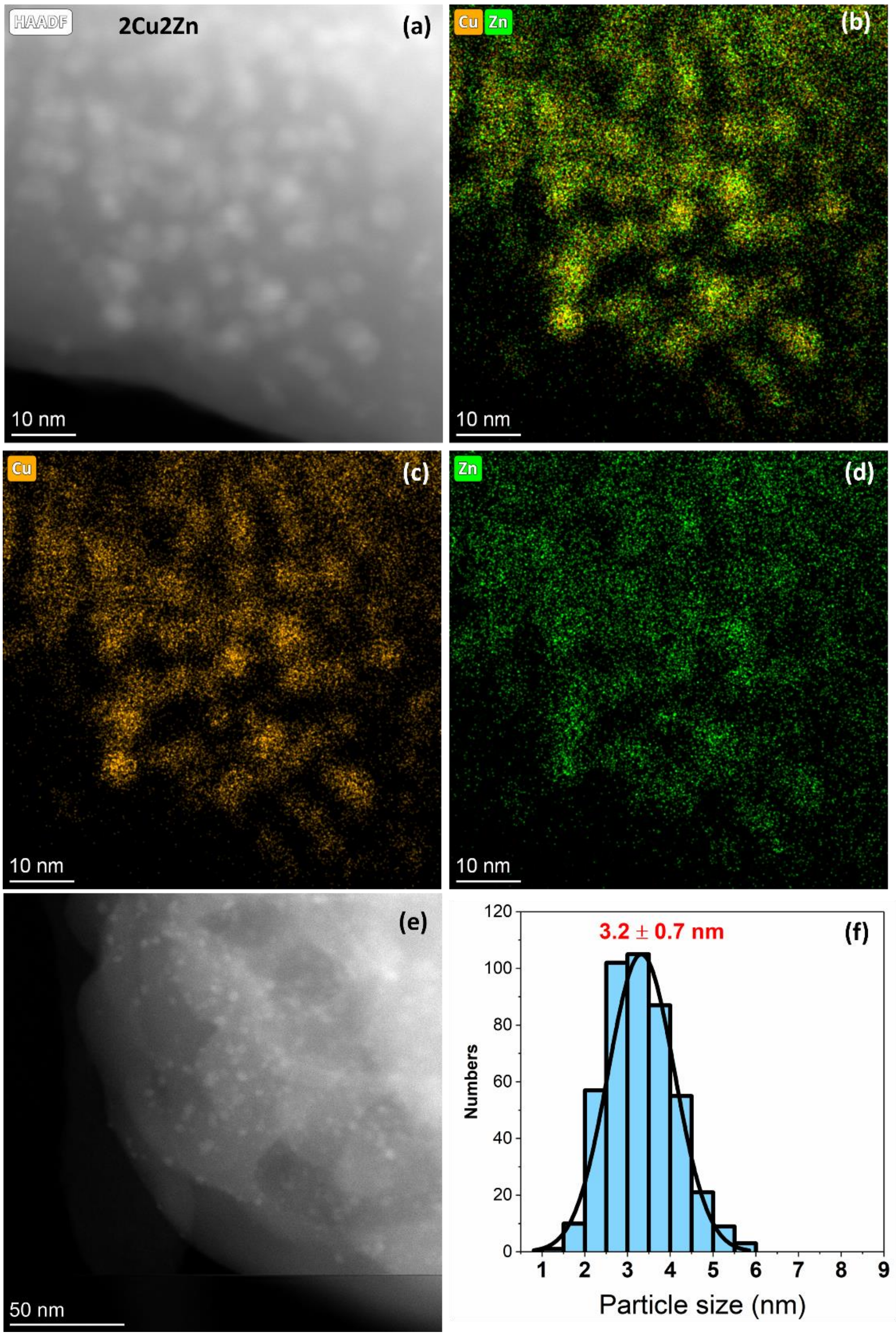

Supplementary Figure 14. (a, e) HAADF-STEM images, (b–d) corresponding EDS elemental maps and (f) Cu-ZnO particle size distribution of the 2Cu2Zn catalyst reduced at 400 °C (mean and standard deviation values are given on the histogram, the particle size distribution was calculated by counting 450 nanoparticles).

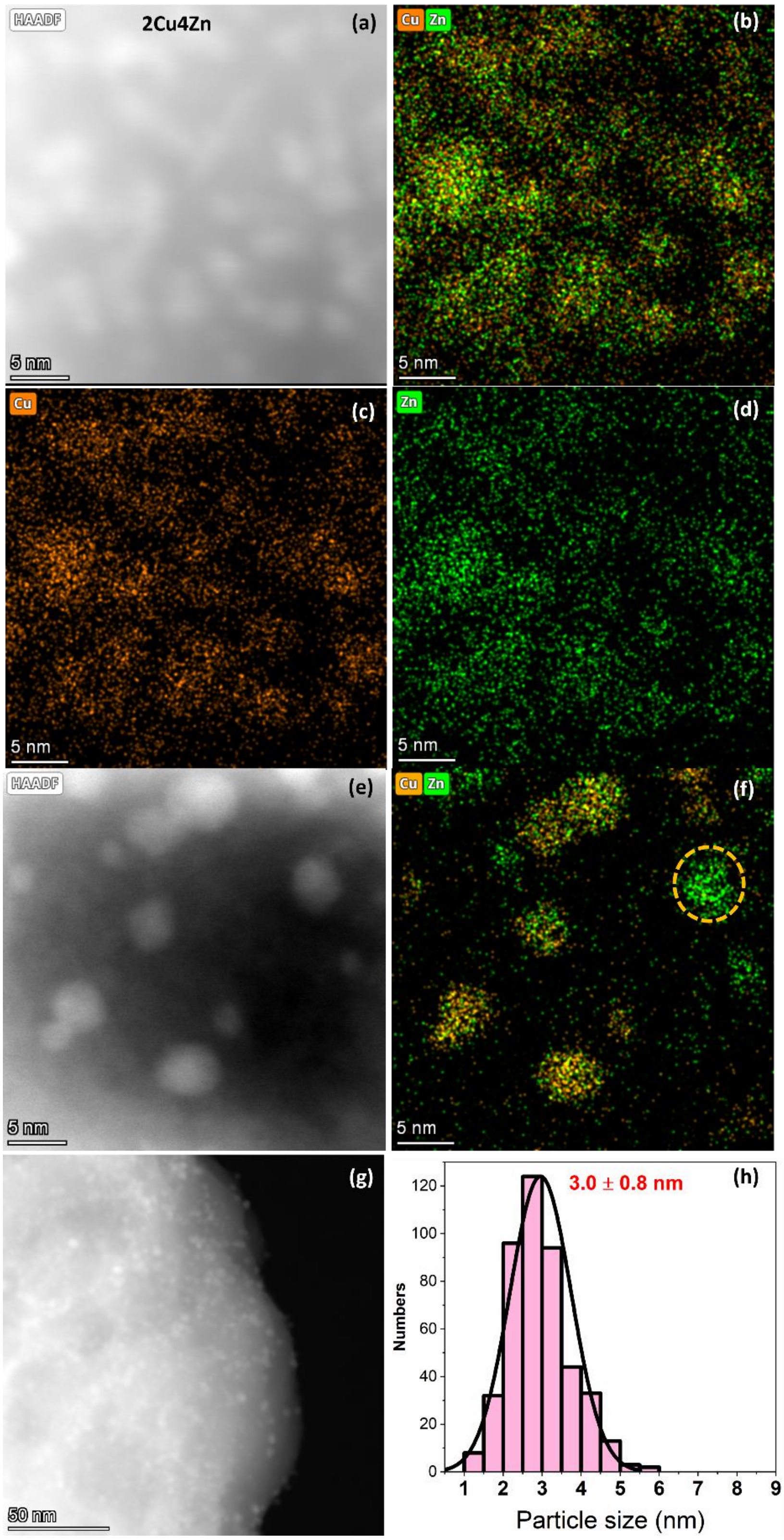

Supplementary Figure 15. (a, e, g) HAADF-STEM images, (b–d, f) corresponding EDS elemental maps and (h) Cu-ZnO particle size distribution of the 2Cu4Zn catalyst reduced at 400 °C (mean and standard deviation values are given on the histogram, the particle size distribution was calculated by counting 450 nanoparticles, orange dash circle highlights the aggregated ZnO nanoparticle).

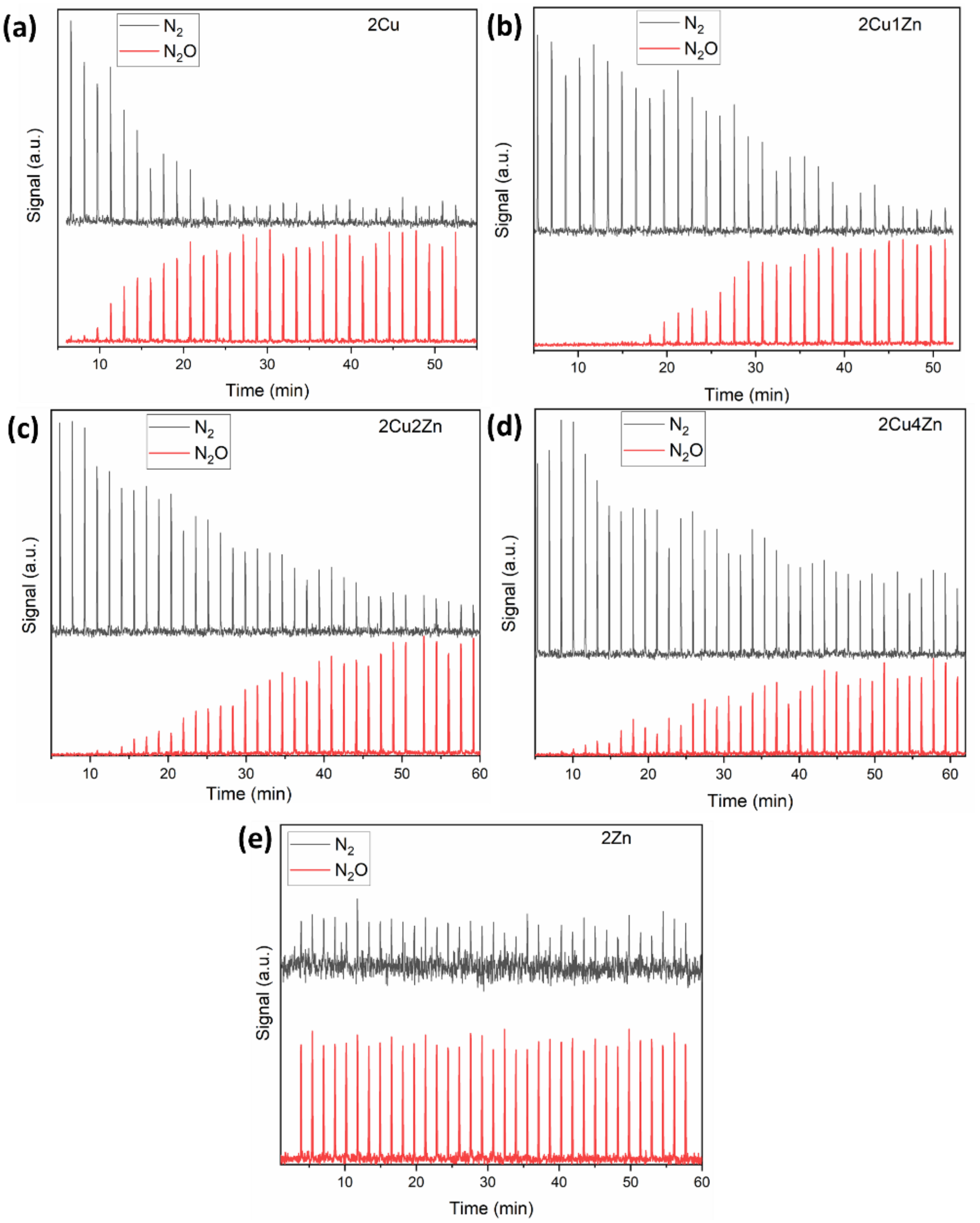

Supplementary Figure 16. Monitoring N_2O consumption and N_2 formation during N_2O pulse chemisorption by MS over (a) 2Cu, (b) 2Cu1Zn, (c) 2Cu2Zn, (d) 2Cu4Zn and (e) 2Zn.

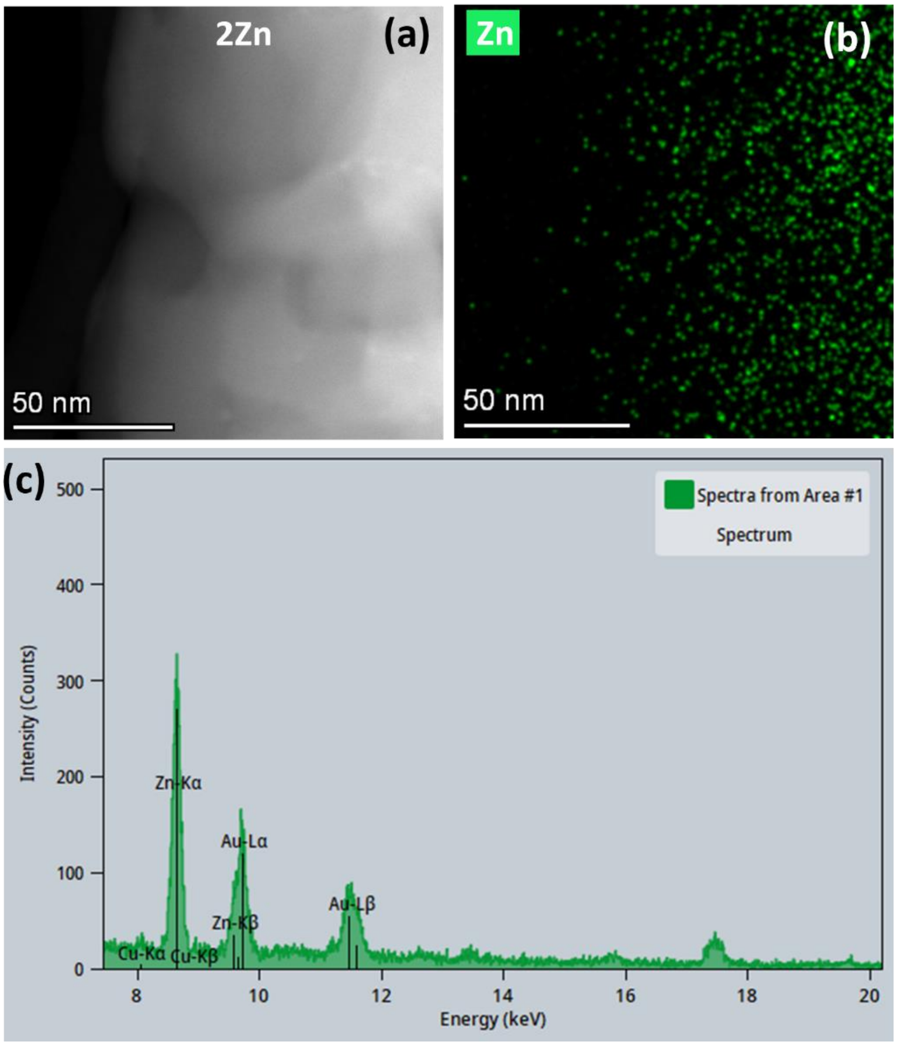

Supplementary Figure 17. (a) HAADF-STEM image, (b-c) corresponding EDX elemental maps of the monometallic 2Zn catalyst reduced at 400 °C.

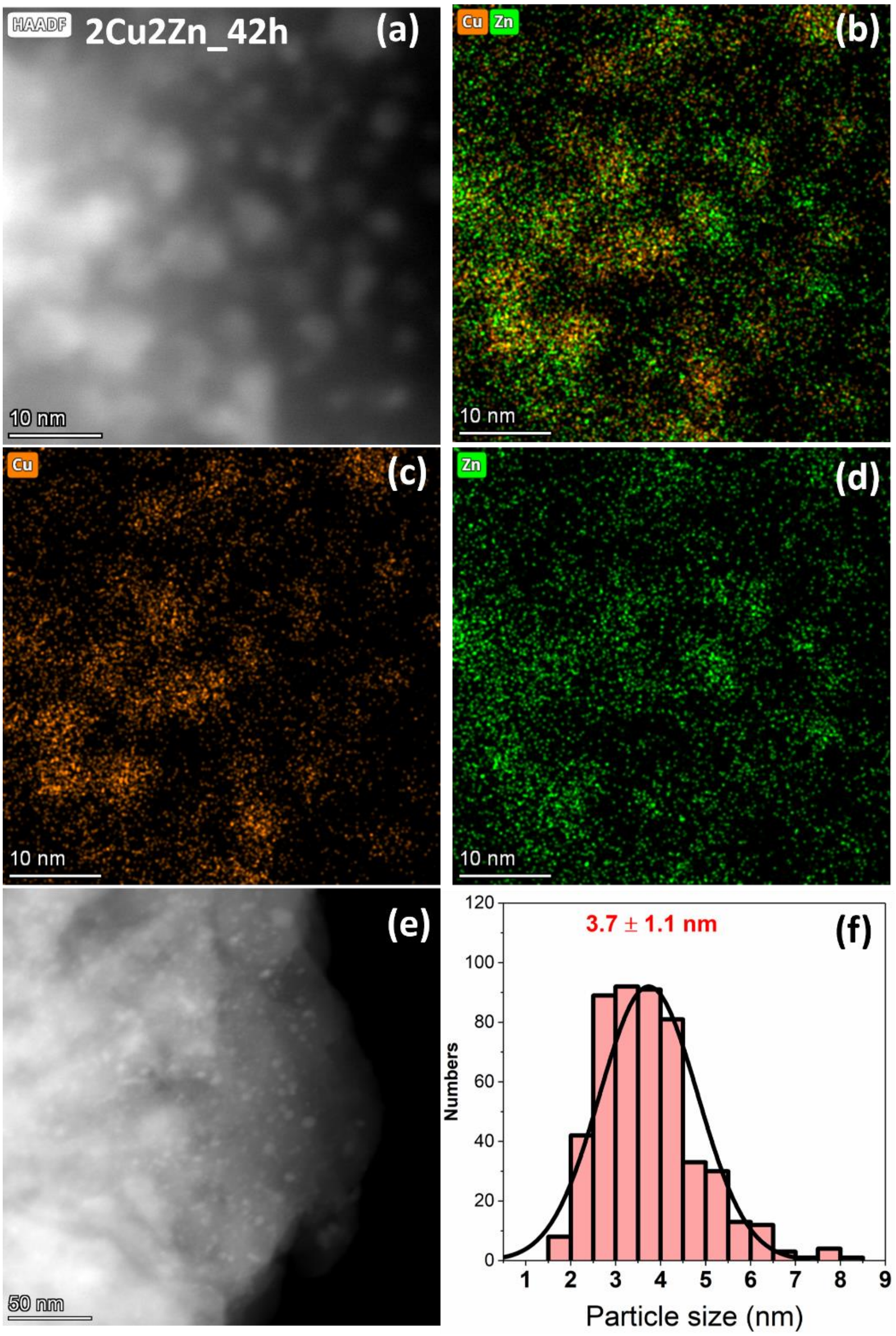

Supplementary Figure 18. (a, e) HAADF-STEM images, (b–d) corresponding EDS elemental maps and (f) Cu-ZnO particle size distribution of spent 2Cu2Zn catalyst after 42 h stability testing under plasma conditions at 14 W (mean and standard deviation values are given on the histogram, the particle size distribution was calculated by counting 500 nanoparticles).

Supplementary Table 6. The percentage of peak area from H_2-TPR for the 2Cu, 2Cu*x*Zn and 2Zn catalysts.

Catalyst	Peak 1 (%)[a] 300−309 °C	Peak 2 (%) 332−360 °C
2Cu	14.4	85.6
2Cu1Zn	31.4	68.6
2Cu2Zn	50.4	49.6
2Cu4Zn	38.8	61.2
2Zn	0	0

[a]Percentage of peak area of (300−309) = Peak area (300−309)/(Peak area (300−309)+ Peak area (332−360))

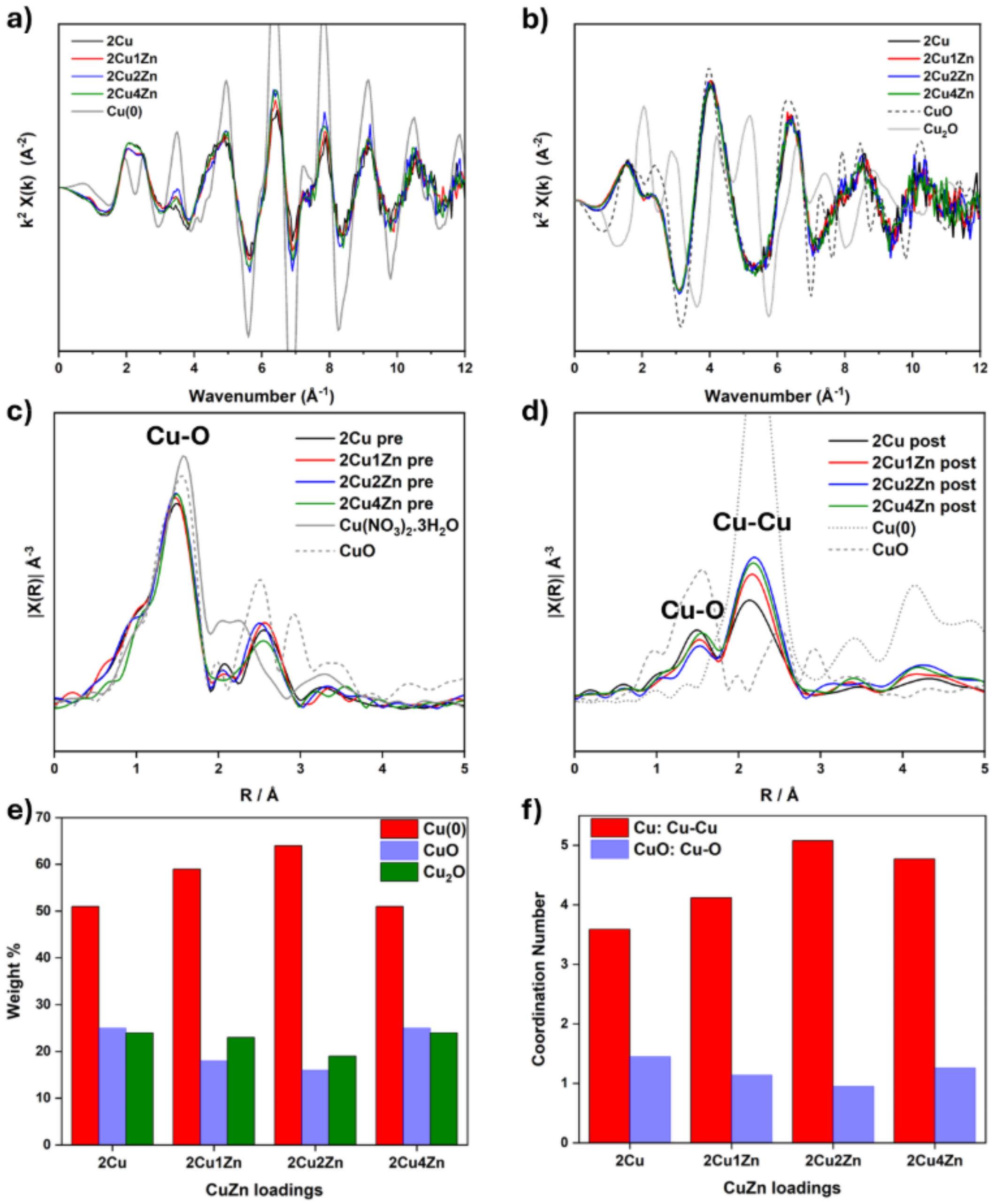

Supplementary Figure 19. Cu K-edge EXAFS, (a-b) k^2 weighted x(k) data and (c-d) Fourier Transform, comparing 2Cu and 2Cu*x*Zn, both pre- and post- hydrogen treatment. The contribution of the Cu-Cu path in Cu^0, and the Cu-O path in CuO, are identified in the fourier transform. (e) LCF fitting of the Cu XANES after hydrogen treatment, showing that the highest quantity of metallic Cu is found in 2Cu2Zn. The Cu(0)% after hydrogen treatment was also given in Table 1 in the main text. (f) Coordination numbers of Cu-O in CuO and Cu-Cu in Cu^0 from EXAFS fitting of the reduced samples.

Supplementary Note 1. Identifying the exact species of the residual oxidised Cu after hydrogen treatment by XAFS is challenging. Firstly, in the XANES, the position of the whiteline is similar for Cu(0) and Cu_2O. Therefore, the calculated fraction of reduced Cu may be artificially lowered by incorrect assignment as Cu_2O during the fit. Secondly, in the EXAFS, the Cu-O first shell distance in Cu_2O and CuO are similar. While they are more easily distinguished as bulk phases, in small nanoparticles such as these, where most of the intensity is in the Cu-Cu path of Cu(0), the error is best minimised by fitting only the Cu-O path in CuO and allowing the interatomic distance to refine, rather than fitting two separate paths. While this was considered the best approach for the XAS data, note that by XPDF analysis Cu_2O gave a better fit to account for the small portion of residual oxidised Cu species. Neither method is deemed to have expected to have produced an accurate fraction of CuO vs Cu_2O.

Supplementary Table 7: EXAFS fitting data for the Cu K-edges of 2Cu and 2Cu*x*Zn both before (pre-reduced) and after (reduced) reduction.

Sample	R-factor	ΔE (eV)	Path	Coordination Number	Bond distance (Å)	Debye-Waller Factor
**2Cu pre-reduced**	0.006	-1.0 ± 0.9	CuO: Cu-O1	3.2 ± 0.1	1.93 ± 0.01	0.004 ± 0.00
			CuO: Cu-O3	0.6 ± 0.2	2.73 ± 0.05	0.003*
			CuO: Cu-Cu	1.6 ± 03	2.94 ± 0.01	0.007 ± 0.00
**2Cu1Zn pre-reduced**	0.008	-1.9 ± 0.8	CuO: Cu-O1	3.3 ± 0.1	1.93 ± 0.01	0.004[a]
			CuO: Cu-O3	0.8 ± 0.2	2.73 ± 0.04	0.003[a]
			CuO: Cu-Cu	1.7 ± 0.2	2.94 ± 0.01	0.007[a]
**2Cu2Zn pre-reduced**	0.004	-1.4 ± 0.6	CuO: Cu-O1	3.4 ± 0.0	1.93 ± 0.05	0.004[a]
			CuO: Cu-O3	0.7 ± 0.2	2.76 ± 0.03	0.003[a]
			CuO: Cu-Cu	1.5 ± 0.2	2.93 ± 0.01	0.007[a]
**2Cu4Zn pre-reduced**	0.004	-0.8 ± 0.5	CuO: Cu-O1	3.2 ± 0.1	1.94 ± 0.00	0.004[a]
			CuO: Cu-O3	0.8 ± 0.2	2.77 ± 0.02	0.003[a]
			CuO: Cu-Cu	1.1 ± 0.1	2.95 ± 0.01	0.007[a]
**2Cu reduced**	0.022	4.2 ± 0.7	CuO: Cu-O[b]	1.5 ± 0.2	1.85 ± 0.01	0.004[a]
			Cu: Cu-Cu	3.6 ± 0.3	2.53 ± 0.01	0.008[a]
**2Cu1Zn reduced**	0.013	3.2 ± 0.6	CuO: Cu-O[b]	1.1 ± 0.2	1.84 ± 0.01	0.004 ± 0.00
			Cu: Cu-Cu	4.1 ± 0.2	2.52 ± 0.00	0.008 ± 0.00
**2Cu2Zn reduced**	0.008	3.5 ± 1.2	CuO: Cu-O[b]	1.0 ± 0.1	-1.86 ± 0.01	0.004[a]
			Cu: Cu-Cu	5.1 ± 0.2	2.53 ± 0.01	0.008[a]

**2Cu4Zn reduced**	0.017	3.4 ± 0.5	CuO: Cu-O[b]	1.3 ± 0.2	1.86 ± 0.01	0.004[a]
			Cu: Cu-Cu	4.8 ± 0.3	2.52 ± 0.00	0.008[a]

[a]The EXAFS of the fresh samples, and separately of the reduced samples, were deemed similar enough that σ^2 was determined for one sample and fixed for the other formulations, in order to reduce the number of components being fit.

[b]The Cu-O path was fit using a CuO FEFF file, however the calculated atomic distance is 0.11 Å smaller than in CuO and is therefore more similar to Cu_2O. However, the Cu-O path is a small contribution to the spectra, and as the XANES predicted a mixed CuO/Cu_2O contribution, the simpler symmetry of the CuO phase allowed better fitting.

** The coordination number of a Cu foil was fixed in line with the crystallographic structure in order to determine the amplitude, and this value of 0.89 applied for all data fitting. A k weighting of 2 was applied for all data.

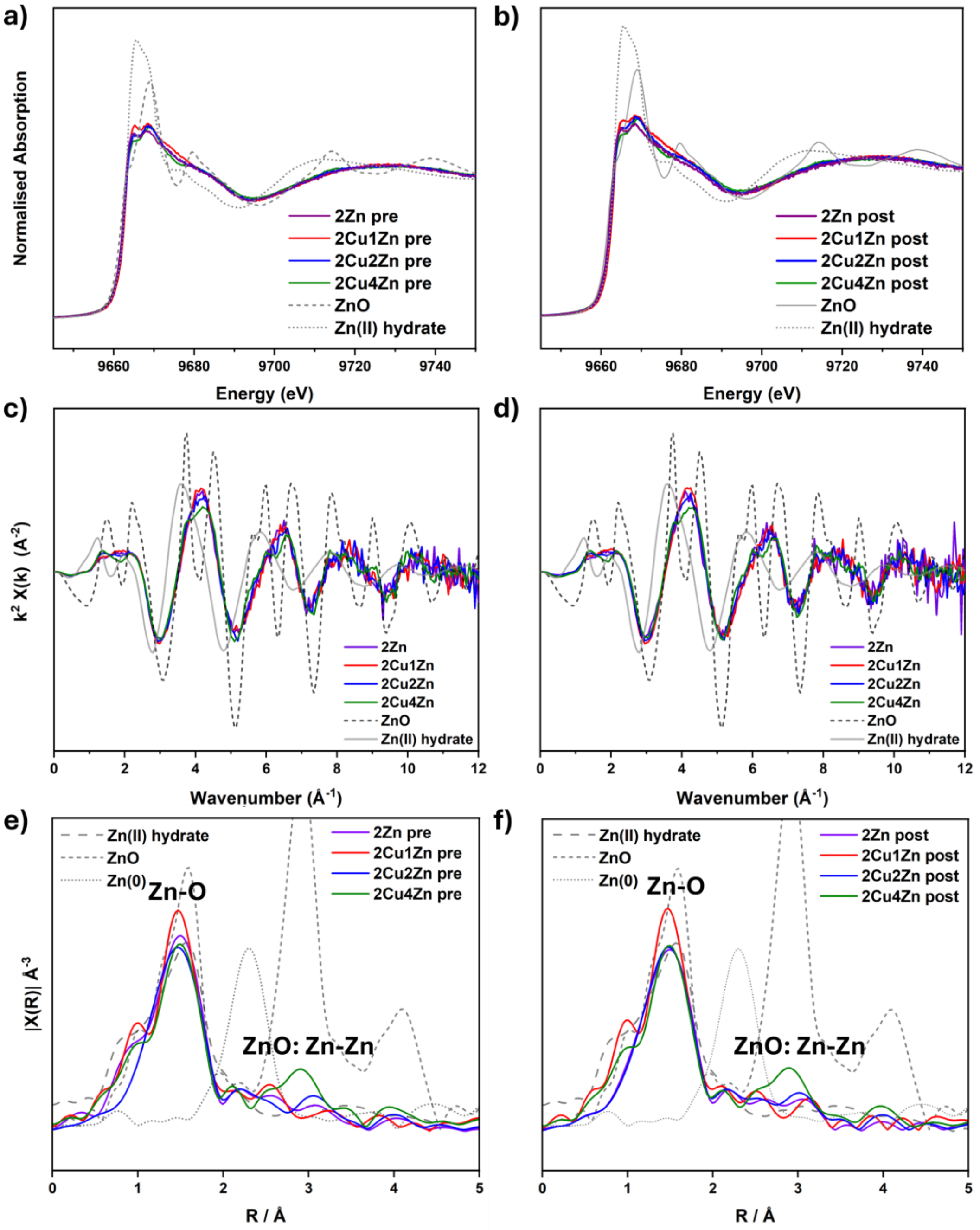

Supplementary Figure 20: (a-b) Zn K-edge EXAFS, (c-d) k^2 weighted x(k) data and (e-f) Fourier Transform, comparing 2Zn and 2Cu*x*Zn, both pre- and post- hydrogen treatment. The contribution of the Zn-O path and the Zn-Zn path in ZnO are identified in the fourier transform. Reference spectra of ZnO, metallic Zn and a hydrated Zn(II) ion in the form of $Zn(NO_3)_2.6H_2O$ are included.

Supplementary Table 8: EXAFS fitting data for the Zn K-edges of 2Zn and 2Cu*x*Zn before and after hydrogen treatment.

Sample	R-factor	ΔE (eV)	Path	Coordination Number	Bond distance (Å)	Debye-Waller Factor
**2Zn pre-reduced**	0.025	2.8 ± 0.7	ZnO: Zn-O	3.7 ± 0.2	1.95 ± 0.01	0.004 ± 0.001
**2Zn reduced**	0.039	2.5 ± 0.7	ZnO: Zn-O	3.6 ± 0.3	1.97 ± 0.01	0.004 ± 0.001
**2Cu1Zn pre-reduced**	0.025	2.2 ± 0.7	ZnO: Zn-O	3.9 ± 0.2	1.97 ± 0.01	0.004 ± 0.001
**2Cu1Zn reduced**	0.026	2.2 ± 0.7	ZnO: Zn-O	4.0 ± 0.2	1.93 ± 0.01	0.004 ± 0.001
**2Cu2Zn pre-reduced**	0.041	1.1 ± 0.8	ZnO: Zn-O	3.7 ± 0.3	1.93 ± 0.01	0.005 ± 0.001
**2Cu2Zn reduced**	0.046	1.3 ± 0.9	ZnO: Zn-O	3.7 ± 0.3	1.93 ± 0.01	0.005 ± 0.001
**2Cu4Zn pre-reduced**	0.012	1.1 ± 0.4	ZnO: Zn-O	3.7 ± 0.2	1.94 ± 0.00	0.005 ± 0.001
			ZnO: Zn-Zn	3.1 ± 0.5	3.25 ± 0.01	0.008 ± 0.001
**2Cu4Zn reduced**	0.034	1.3 ± 0.9	ZnO: Zn-O	3.7 ± 0.2	1.94 ± 0.01	0.005 ± 0.001
			ZnO: Zn-Zn	3.4 ± 0.5	3.24 ± 0.01	0.008 ± 0.001

** A Zn foil of with sufficient quality EXAFS for fitting could not be obtained, so an amp of 0.8 was used. A k weighting of 2 was applied for all data. Only the fit of 2Cu4Zn was improved by addition of a Zn-Zn path from ZnO.

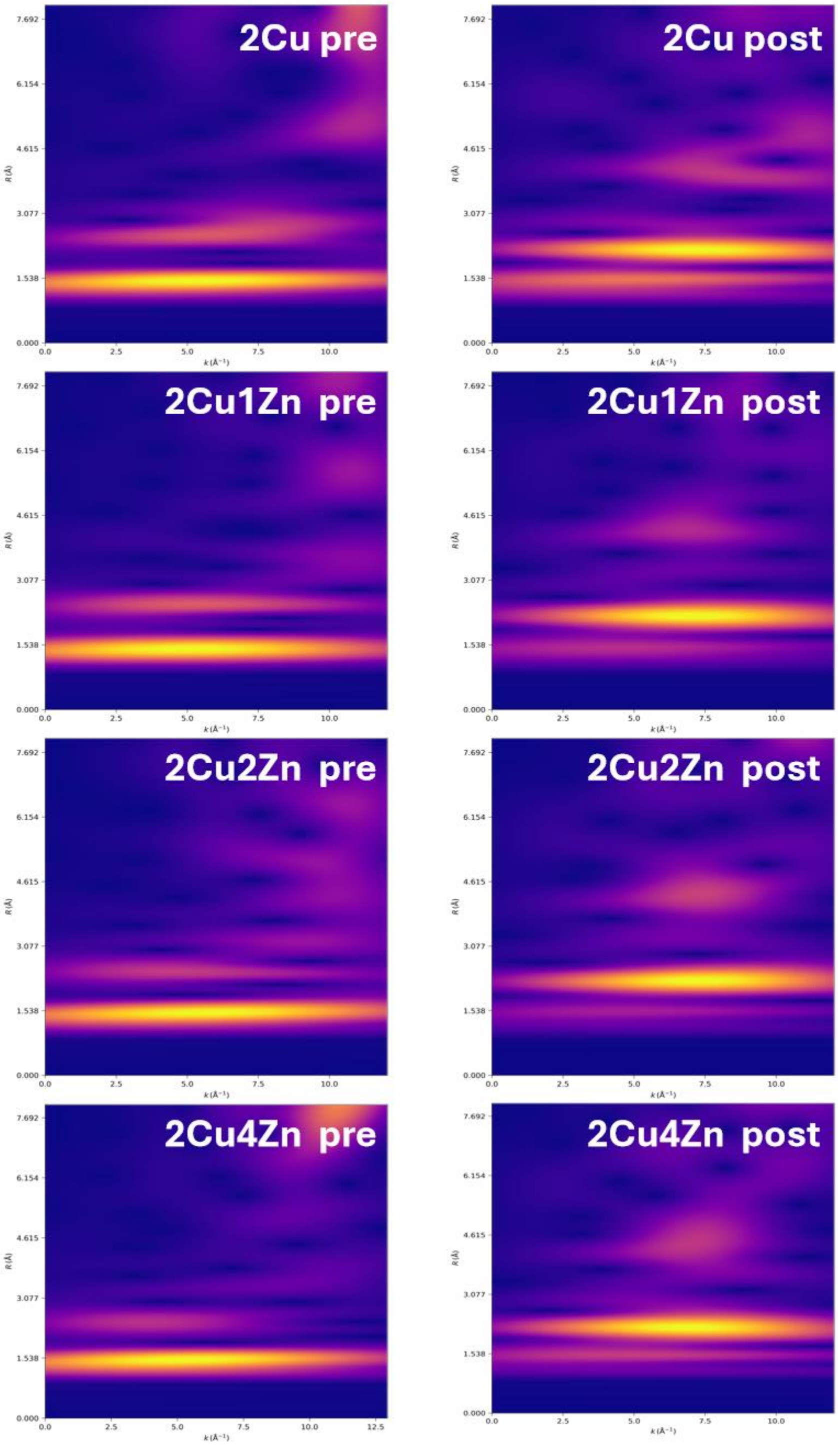

2Cu pre
2Cu post
2Cu1Zn pre
2Cu1Zn post
2Cu2Zn pre
2Cu2Zn post
2Cu4Zn pre
2Cu4Zn post
R (Å)
k (Å⁻¹)
7.692
6.154
4.615
3.077
1.538
0.000
0.0
2.5
5.0
7.5
10.0
12.5

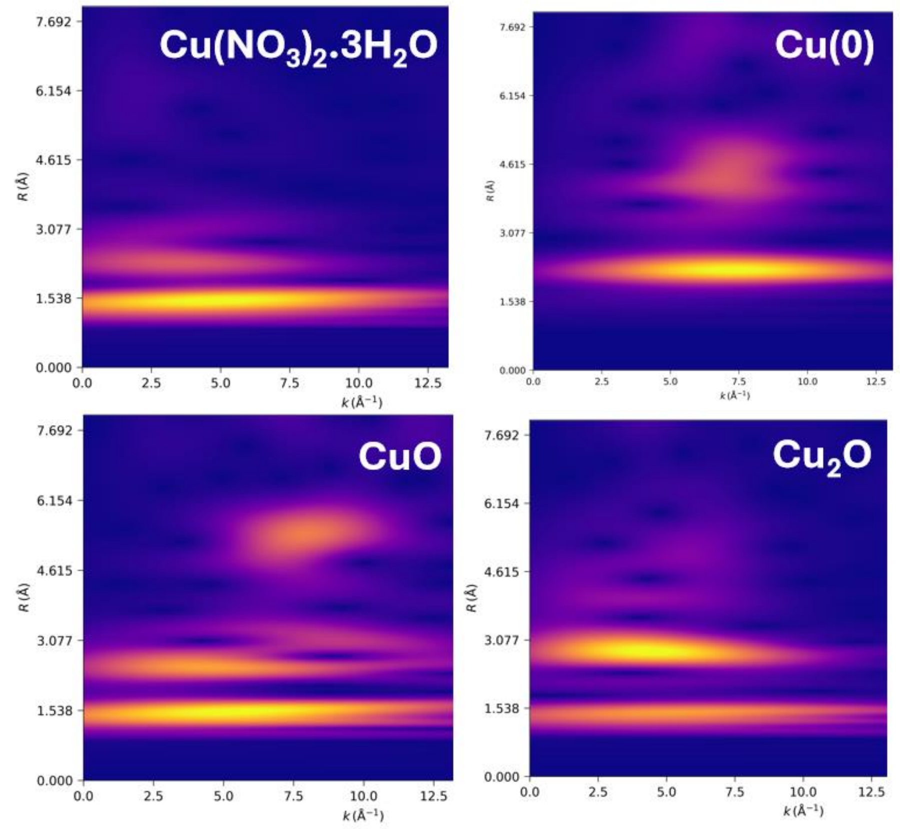

Supplementary Figure 21. The wavelet transforms analysis for the Cu K-edge EXAFS spectra over 2Cu and 2Cu*x*Zn pre- and post- reduction, along with references (analysis with the Larch package[17]).

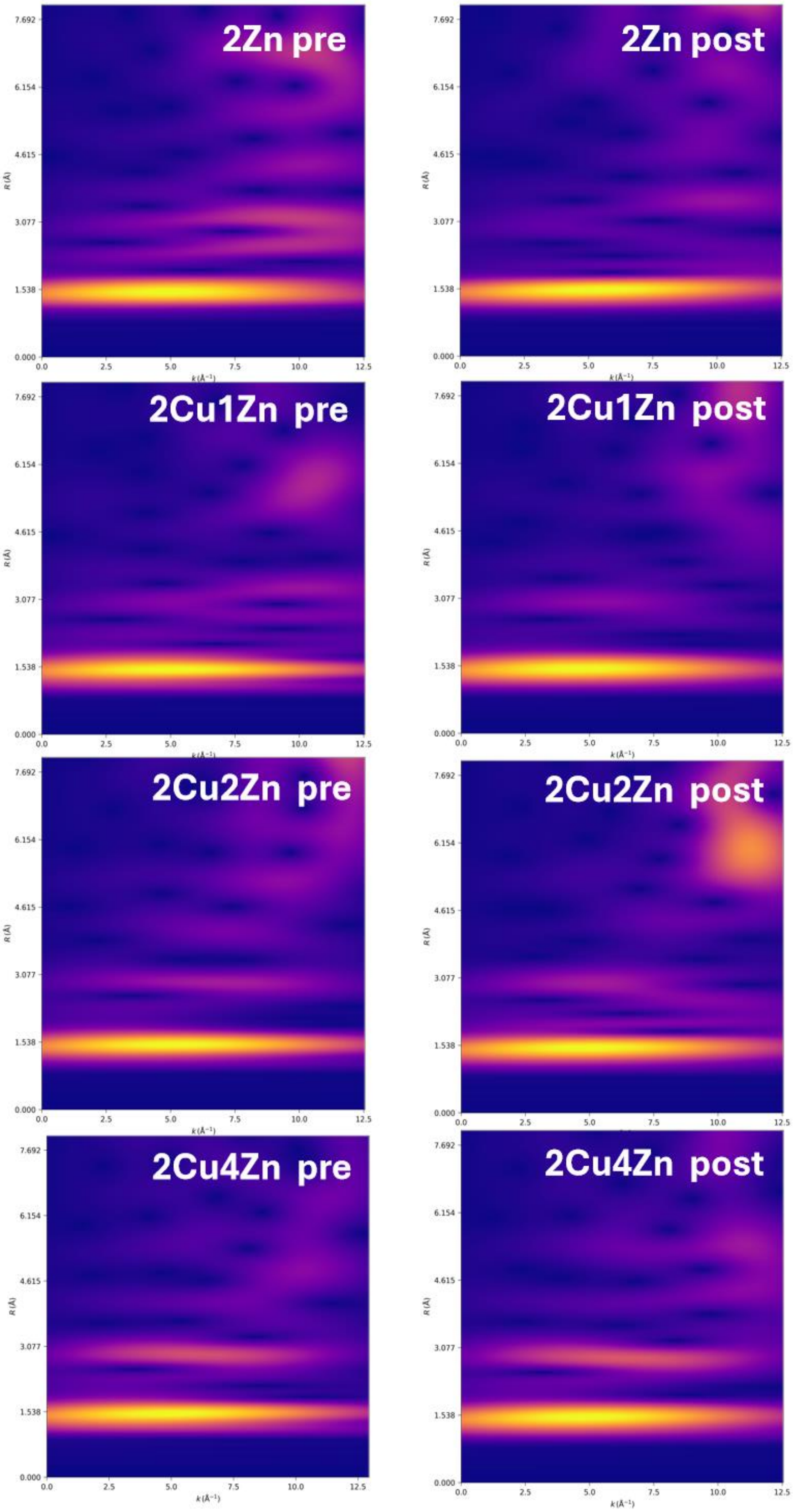
2Zn pre
2Zn post
2Cu1Zn pre
2Cu1Zn post
2Cu2Zn pre
2Cu2Zn post
2Cu4Zn pre
2Cu4Zn post

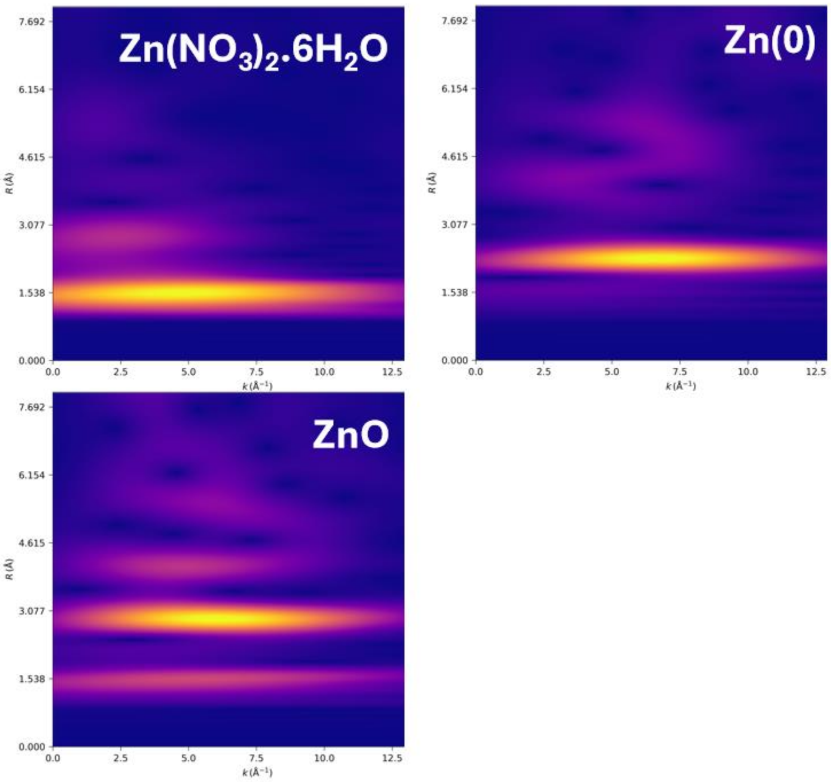

Supplementary Figure 22. The wavelet transforms for the Zn K-edge EXAFS spectra over 2Zn and 2Cu*x*Zn pre- and post- hydrogen treatment, along with references (analysis with the Larch package[17]). Note the intensity at approximately 6 Å in the 2Cu2Zn post hydrogen treatment sample is not expected to have any real chemical meaning, corresponding to a very large atomic distance.

Supplementary Note 2. *In situ* X-ray pair distribution function (XPDF) during thermal hydrogen treatment

In situ X-ray pair distribution function (XPDF) was performed to probe the phase structures of Cu and Zn before and post hydrogen treatment at 400 °C. Prior to treatment, both diffraction patterns of the cubic copper oxide phase were found in the calcined 2Cu, 2Cu2Zn and 2Cu4Zn catalysts (Supplementary Table 9). However, after hydrogen treatment at 400 °C for 1 h, the Cu oxide in 2Cu2Zn and 2Cu4Zn was completely reduced to metallic cubic Cu, whilst 2Cu catalyst exhibited the co-presence of metallic Cu (~76.4 mol.%) and Cu_2O (~23.6 mol.%) phases (Supplementary Table 9), confirming the findings by XAS (*i.e.*, 2Cu*x*Zn is more reduced than 2Cu). However, while both Cu_2O and CuO were identified by XANES as minor phases in the samples after hydrogen treatment, Cu(0) and Cu_2O were sufficient to fit the XPDF. Please see Supplementary Note 1 above for further explanation. Fitting the Zn phases in 2Cu2Zn, 2Cu4Zn and 2Zn show no changes after hydrogen treatment, and only the hexagonal (wurtzite) ZnO phase was observed, confirming the dominant presence of ZnO in the 2Cu2Zn and 2Zn after the thermal hydrogen treatment. The almost identical ZnO PDF data in 2Zn, 2Cu2Zn and 2Cu4Zn further demonstrated no alloy formation (Supplementary Table 9).

Supplementary Table 9. Structure parameters of Cu and Zn phases from XPDF refinements in 2Cu, 2Cu2Zn, 2Cu4Zn and 2Zn before (pre) and after (post) hydrogen treatment. The ZSM-5 phase was refined freely to fully account for its contribution to the PDF data. However, despite the low Rwp value (1.1%), the complete fit of the bare ZSM-5 is not reported, as such refinement methods do not yield meaningful structural information. Atomic positions for the Cu and Zn containing phases were not refined. For atomic displacement parameters, Uiso values were fixed to 0.01 for each Cu, Zn and O, in all phases.

Sample	R_{wp} (%)	Phase	Space-Group	Wt.%	Mol.%	Lattice Parameter (**Å** or **°**)
ZSM-5	1.1	ZSM-5	P121/n1	100.0	100.0	a = 20.33, b = 21.10, c = 13.83, α = 89.57, β = 89.37, γ = 89.57
2Cu pre	7.4	Cu_2O	Pn-3m	76.6	64.5	4.27
		CuO	Pm-3m	23.4	35.5	3.91
2Cu post	7.2	Cu	Fm-3m	59.0	76.4	3.62
		Cu_2O	Pn-3m	41.0	23.6	4.36
2Cu2Zn pre	6.7	Cu_2O	Pn-3m	34.4	22.9	4.25
		CuO	Pm-3m	8.7	10.4	3.90
		ZnO	P6_3mc	56.9	66.7	3.22, 5.02
	7.2	Cu	Fm-3m	49.2	55.4	3.62

2Cu2Zn post		ZnO	P6_3mc	50.8	44.6	3.10, 5.26
2Cu4Zn pre	11.0	Cu_2O	Pn-3m	25.3	16.1	4.34
		CuO	Pm-3m	4.3	4.9	3.92
		ZnO	P6_3mc	70.5	79.0	3.25, 5.20
2Cu4Zn post	8.6	Cu	Fm-3m	34.2	40.0	3.62
		ZnO	P6_3mc	65.8	60.0	3.18, 5.20
2Zn pre	5.4	ZnO	P6_3mc	100.0	100	3.21, 5.20
2Zn post	6.2	ZnO	P6_3mc	100.0	100	3.18, 5.12

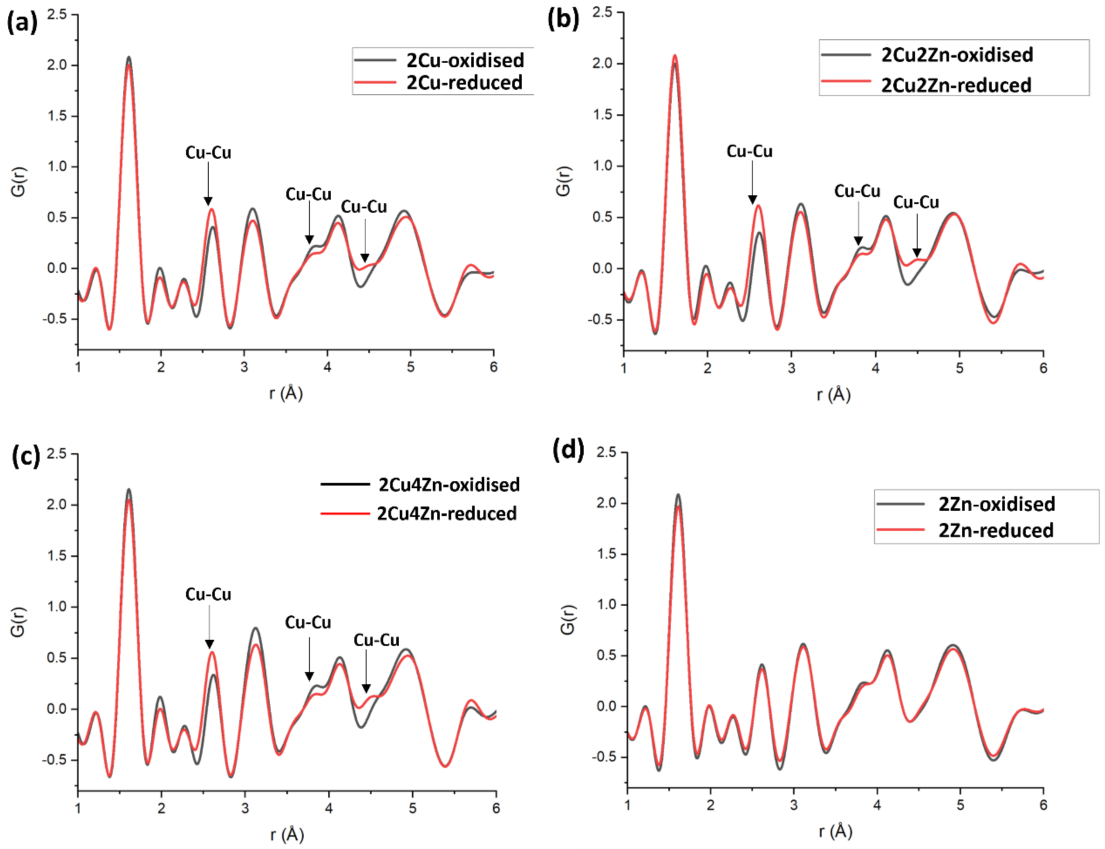

Supplementary Figure 23. Comparing Pair distribution function, G(r), before (obtained at 50 °C) and after reduction (obtained at 50 °C after hydrogen treatment at 400 °C for 1 hr) of (a) 2Cu, (b) 2Cu2Zn, (c) 2Cu4Zn and (d) 2Zn.

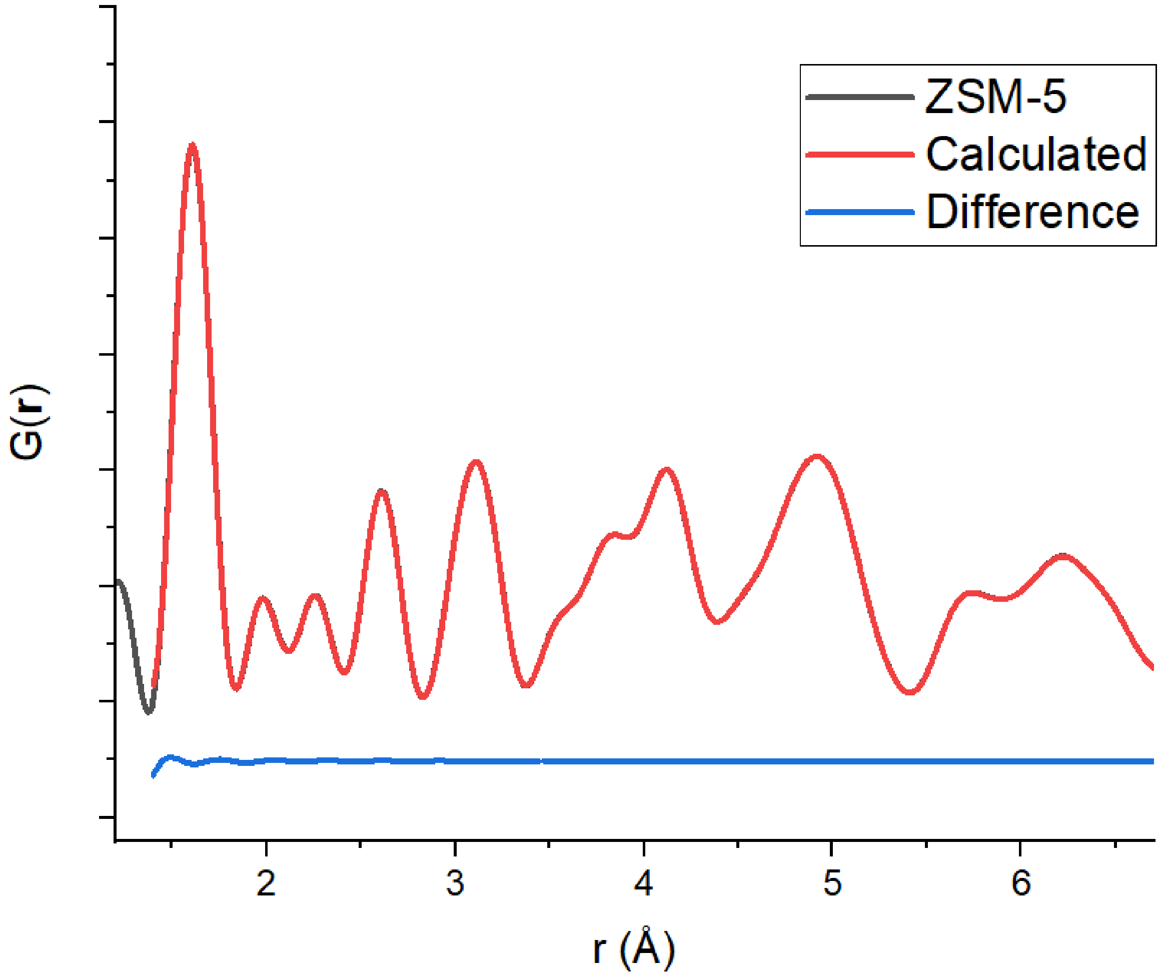

Supplementary Figure 24. PDF refinements of ZSM-5 only sample after hydrogen treatment at 400 °C. (Refinement with a monoclinic ZSM-5).

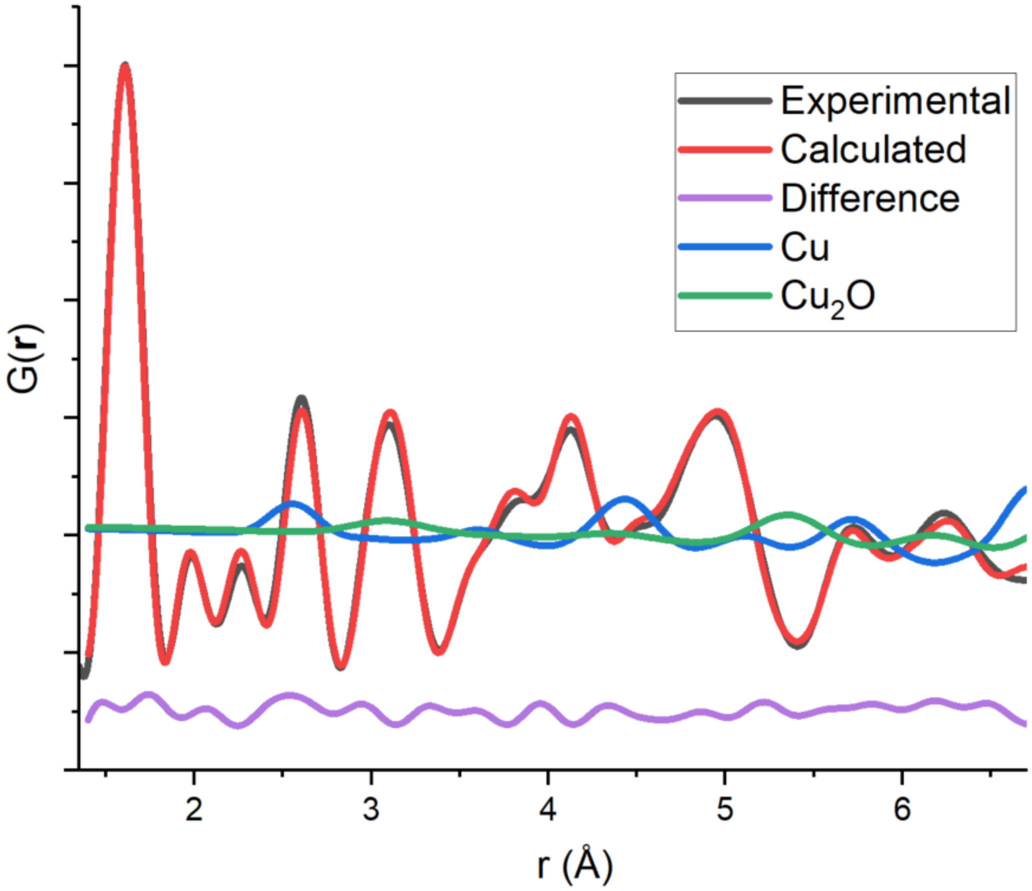

Supplementary Figure 25. PDF refinements of 2Cu catalyst after hydrogen treatment at 400 °C. (Refinement with cubic metallic Cu and cubic Cu_2O).

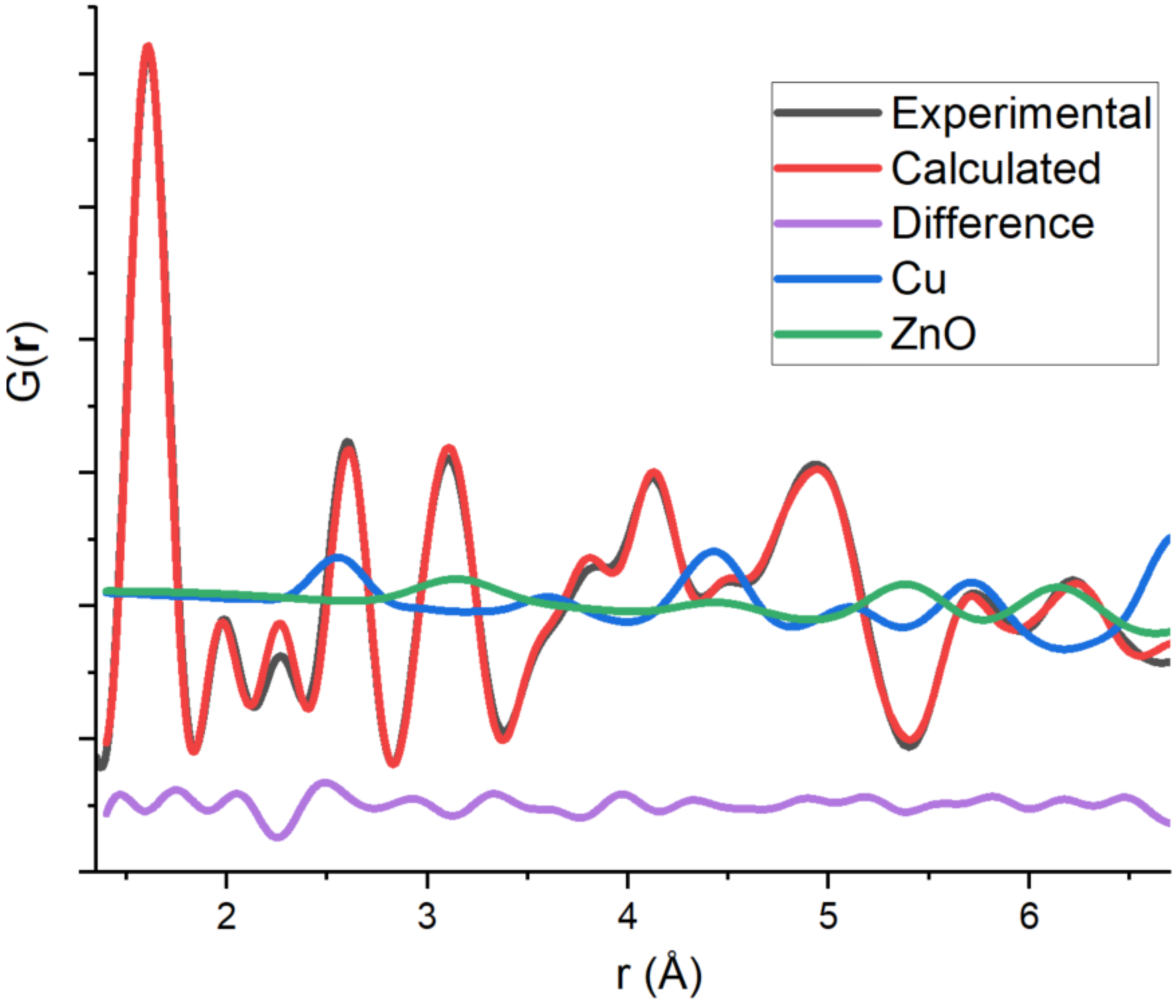

Supplementary Figure 26. PDF refinements of 2Cu2Zn catalyst after hydrogen treatment at 400 °C. (Refinement with cubic metallic Cu and hexagonal ZnO).

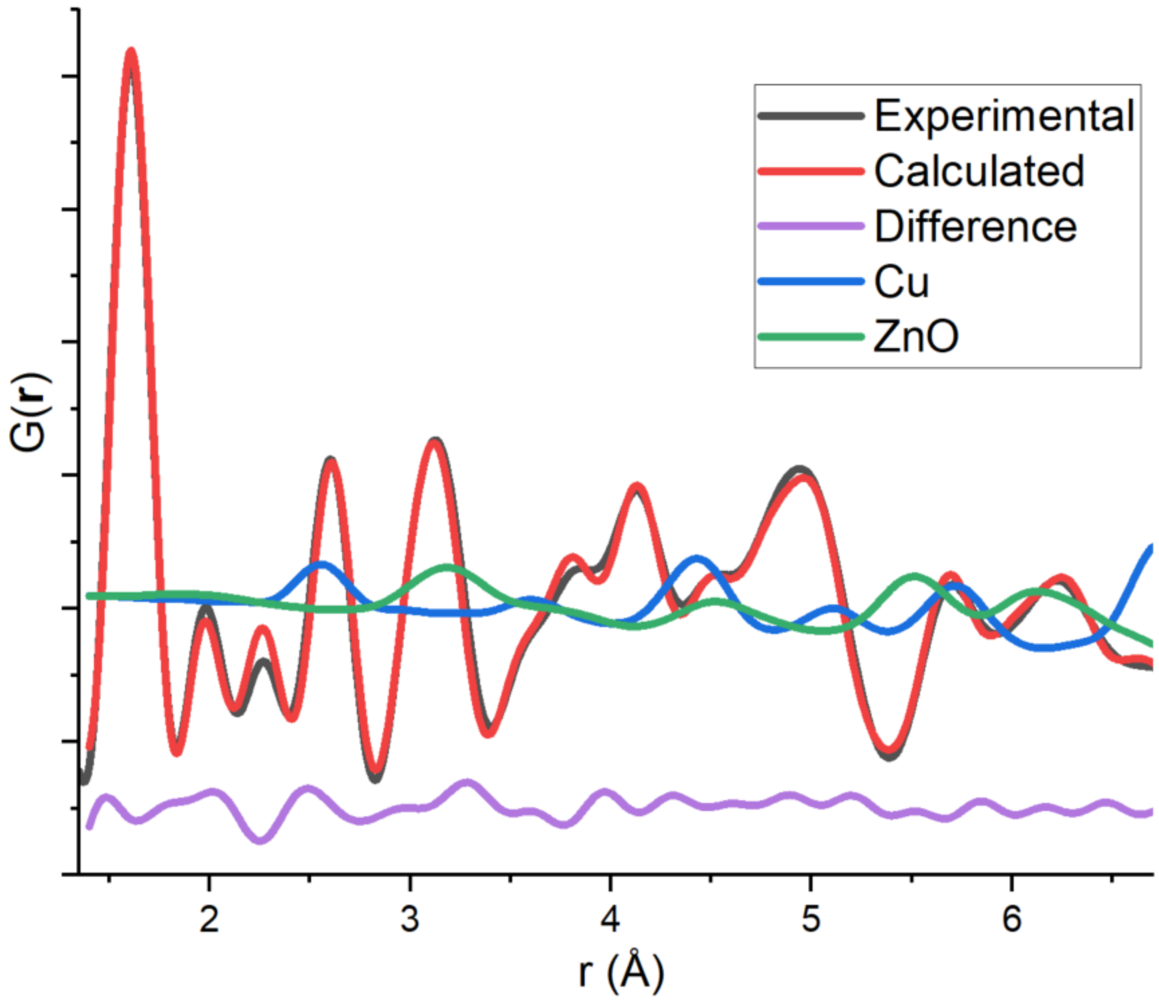

Supplementary Figure 27. PDF refinements of 2Cu4Zn catalyst after hydrogen treatment at 400 °C. (Refinement with cubic metallic Cu and hexagonal ZnO).

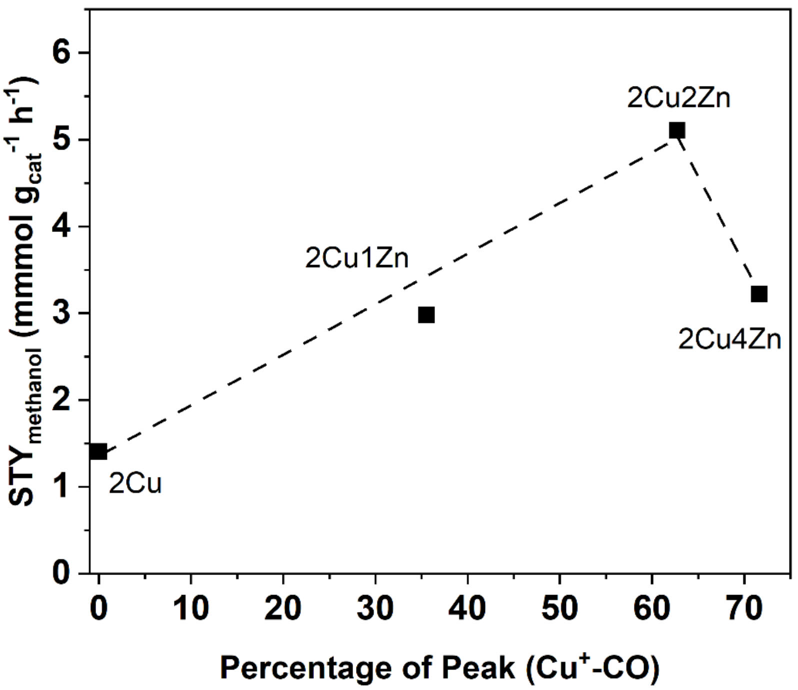

Supplementary Figure 28. The linear correlation between the percentage of Cu^{+}-CO (from CO-DRIFTS) and methanol STY.

Supplementary Table 10. The percentage of interaction between CO and Cu species from in situ CO-DRIFTS.

Catalyst	Cu^{0}-CO (%)<br>2110 cm^{-1}	Cu^{+}-CO (%)<br>2130 cm^{-1}
2Cu	100	0
2Cu1Zn	64.5	35.5
2Cu2Zn	37.3	62.7
2Cu4Zn	28.4	71.6

Supplementary Note 3. *Operando* XAS for NTP catalytic CO_2 hydrogenation to methanol.

Steady-State XAS experiments. The MS profile (Supplementary Figs. 29 and 30) shows that 2Cu and 2Cu2Zn were inactive for CO_2 hydrogenation without NTP. However, upon plasma ignition, the immediate increase of CO, methanol and CH_4 signals in the MS profiles, along with a decrease in CO_2 signal, confirmed the activity of the NTP catalytic systems. The conversions over 2Cu and 2Cu2Zn catalysts were ~5.7% and ~8.0% at NTP power of 3.0 and 4.5 W, respectively. However, the yield of methanol over 2Cu2Zn were ~2.5 times of that over 2Cu, confirming the CuZn catalyst promote the methanol formation.

Comparing the wavelet analysis on the Cu edge (Supplementary Fig. 35), 2Cu and 2Cu2Zn, under 11 kV reaction conditions and post-reaction spectra, showed no noticeable differences, and certainly there is no evidence of new signals that could be attributed to Cu-Zn bonding. Similarly, the Zn edge was also considered (Supplementary Fig. 36) and again showed no notable differences between the 2Zn and 2Cu2Zn species. Thus, we can conclude that NTP does not affect the Zn environment, remaining ZnO throughout the system.

Cycled XAS experiments. The MS profile (Supplementary Figs. 37 and 38) shows that with 20%CO_2/Ar flowing, once plasma was ignited, the immediate increase of CO and O_2 signals in the MS profiles, along with a decrease in CO_2 signal, confirming CO_2 dissociation to produce oxidative species (e.g., O_2 and O*) in the system. When the gas feed changed to 20%CO_2/60%H_2/Ar, the methanol was produced, confirming the activity of the NTP catalytic systems.

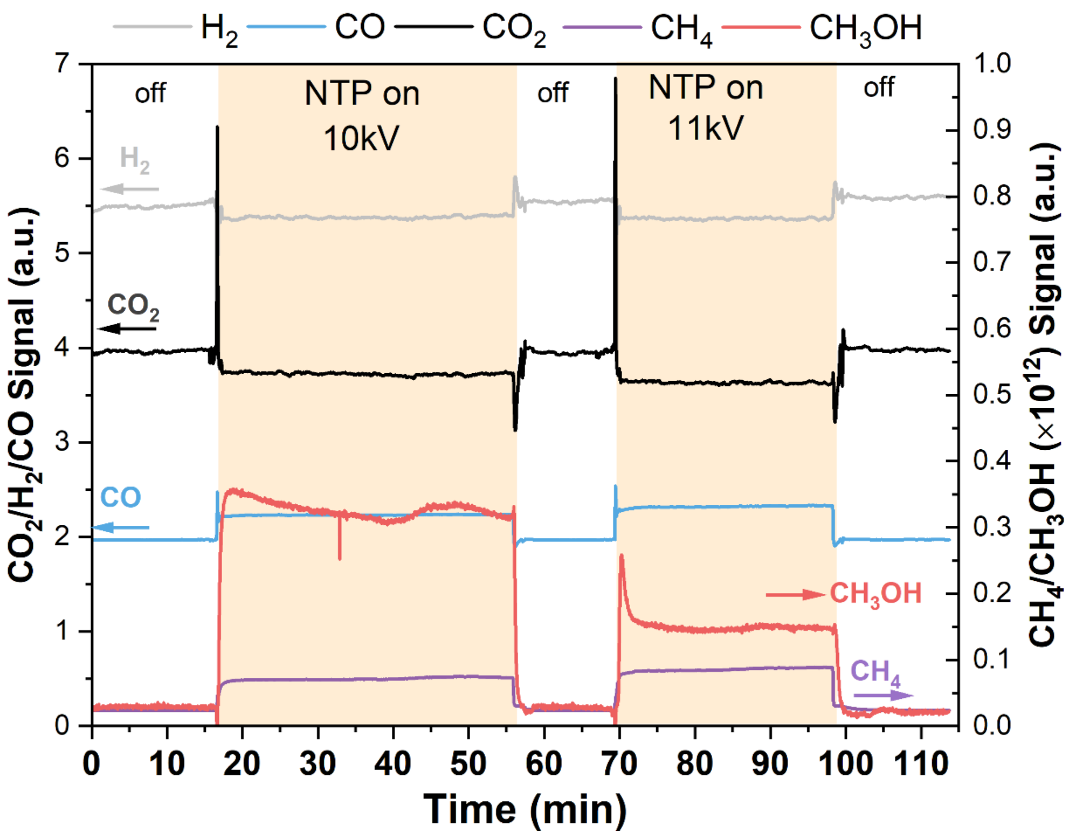

Supplementary Figure 29. MS signals collected from steady state *operando* XAS experiments as a function of time over the 2Cu/ZSM-5 catalyst during the NTP catalysis (gas feed = 20%CO_2/60%H_2/Ar; total flow rate = 20 ml min^{-1}, NTP system: 10 and 11kV, corresponding to 3.0 W and 4.5 W).

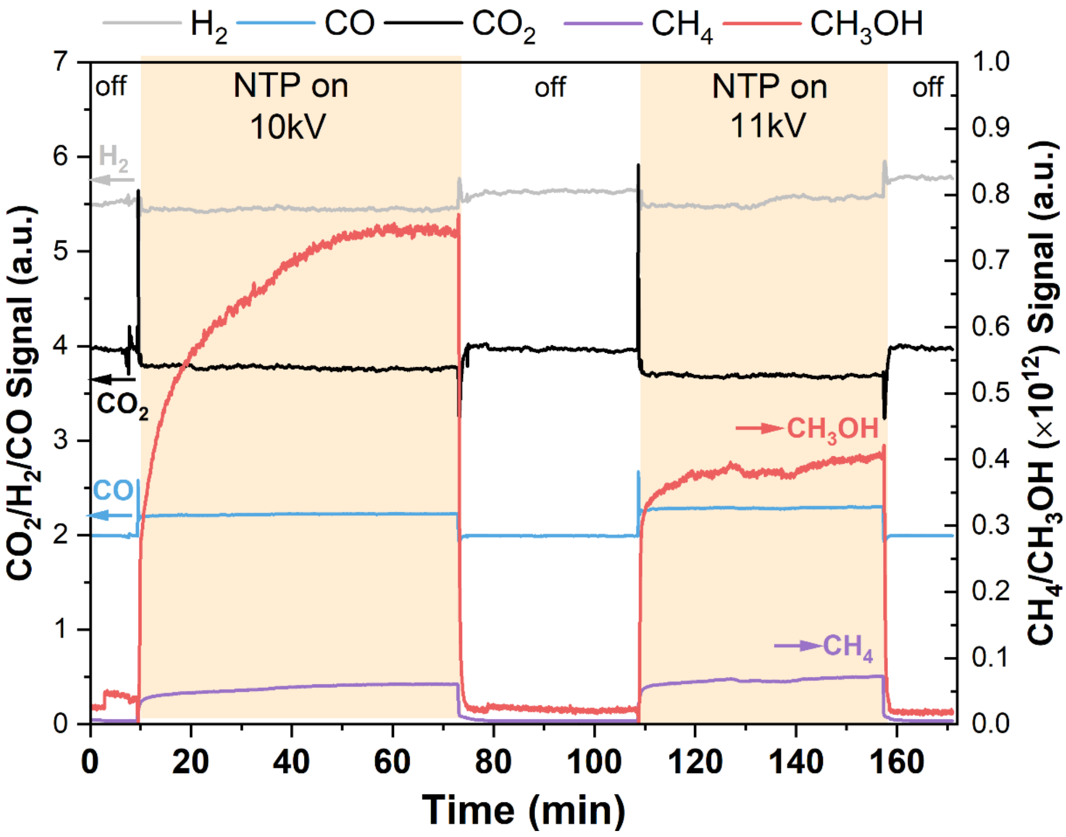

Supplementary Figure 30. MS signals collected from steady state *operando* XAS experiments as a function of time over the 2Cu2Zn/ZSM-5 catalyst during the NTP catalysis (gas feed = 20%CO_2/60%H_2/Ar; total flow rate = 20 ml min^{-1}, NTP system: 10 and 11kV, corresponding to 3.0 W and 4.5 W).

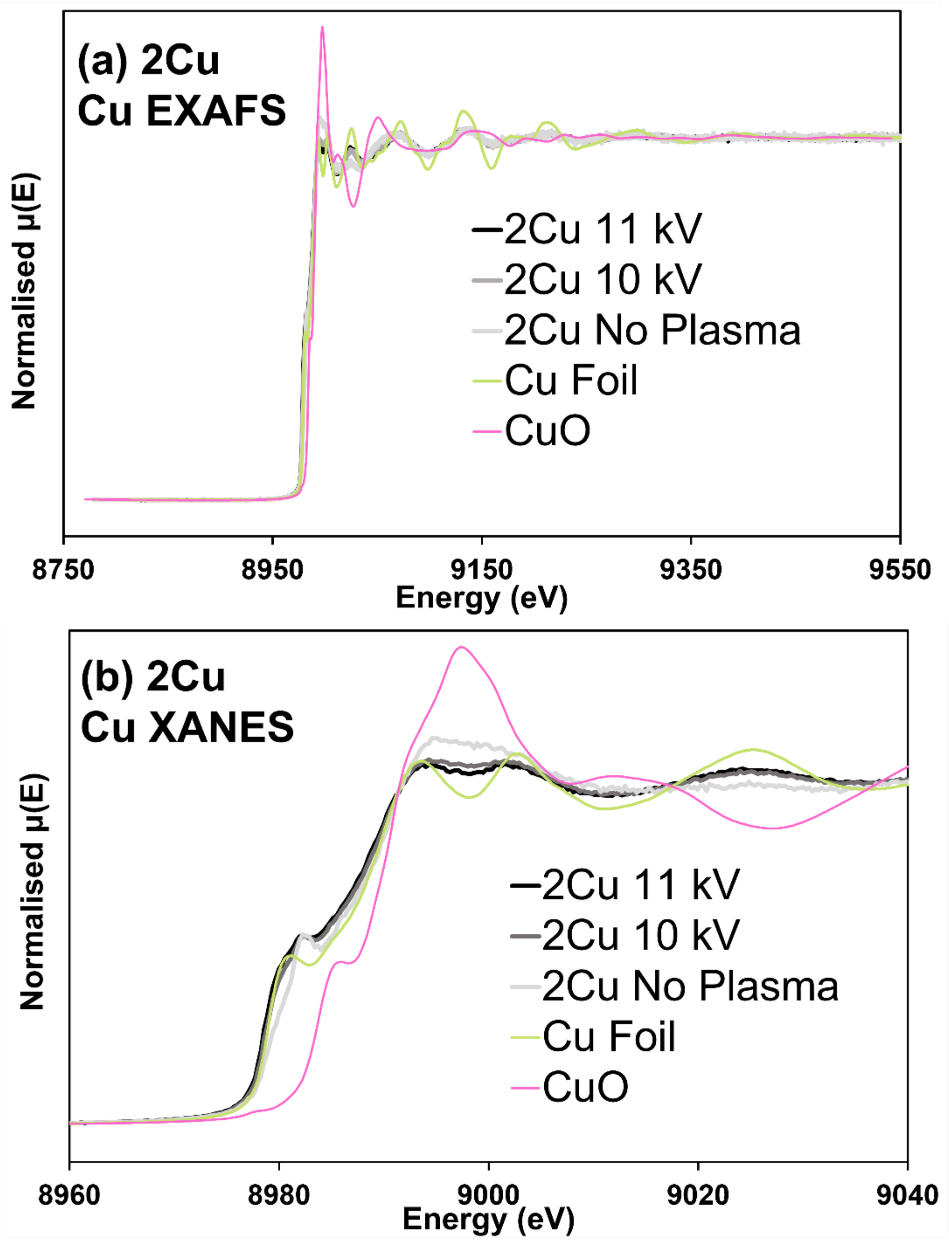

Supplementary Figure 31. The influence of NTP voltage/power in CO_2 hydrogenation conditions under plasma conditions for 2Cu, focussing on the Cu K-edge (a) EXAFS and (b) XANES regions.

Supplementary Table 11: Cu K-edge EXAFS fitting parameters for 2Cu, at different applied voltages.

System	R-factor	ΔE (eV)	Path	Coordination Number	Bond Distance (Å)	Debye-Waller Factor
**2Cu 11 kV**	0.005	1.0 ± 1.1	Cu-O	0.46 ± 0.21	1.82 ± 0.02	0.002 ± 0.006
			Cu-Cu	7.10 ± 0.80	2.50 ± 0.01	0.0014 ± 0.001
**2Cu 10 kV**	0.007	0.7 ± 1.4	Cu-O	0.74 ± 0.32	1.82 ± 0.02	0.005 ± 0.007
			Cu-Cu	6.49 ± 0.88	2.50 ± 0.01	0.012 ± 0.001
**2Cu No Plasma**	0.032	-0.3 ± 4.0	Cu-O	1.98 ± 0.91	1.84 ± 0.03	0.006 ± 0.007
			Cu-Cu	4.02 ± 1.50	2.52 ± 0.03	0.009 ± 0.003

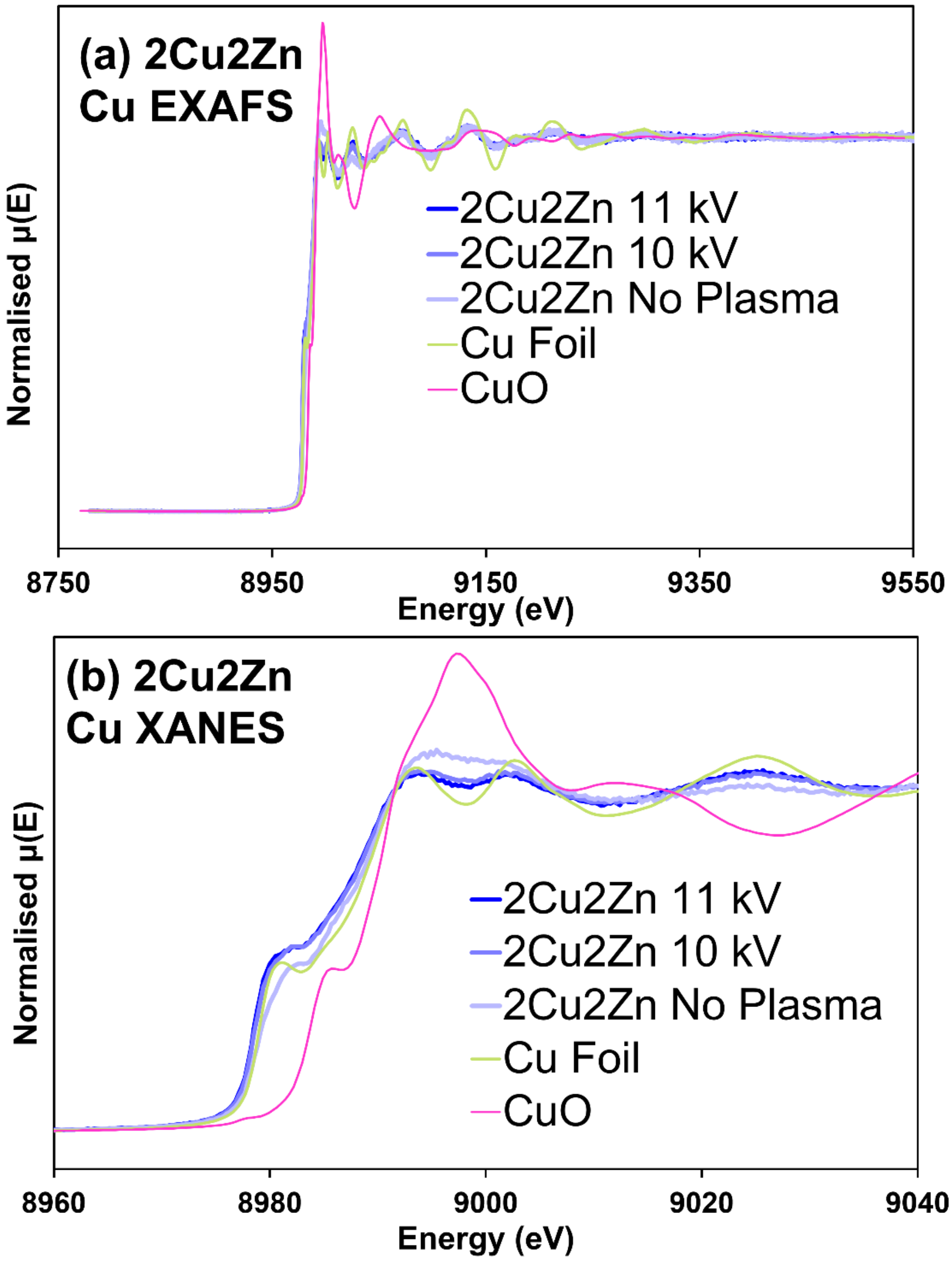

Supplementary Figure 32. The influence of NTP voltage/power under CO_2 hydrogenation conditions for 2Cu2Zn, focussing on the Cu K-edge (a) EXAFS and (b) XANES regions.

Supplementary Table 12: Cu K-edge EXAFS fitting parameters for 2Cu2Zn, at different applied voltages.

System	R-factor	ΔE (eV)	Path	Coordination Number	Bond Distance (Å)	Debye-Waller Factor
2Cu2Zn 11 kV	0.013	1.4 ± 1.2	Cu-Cu	7.38 ± 0.95	2.51 ± 0.01	0.0012 ± 0.001
2Cu2Zn 10 kV	0.006	1.9 ± 1.2	Cu-O	0.46 ± 0.31	1.84 ± 0.03	0.005 ± 0.010
			Cu-Cu	6.98 ± 0.83	2.51 ± 0.01	0.012 ± 0.001
2Cu2Zn No Plasma	0.012	1.7 ± 2.0	Cu-O	1.13 ± 0.56	1.84 ± 0.02	0.006 ± 0.008
			Cu-Cu	5.48 ± 1.01	2.52 ± 0.01	0.009 ± 0.002

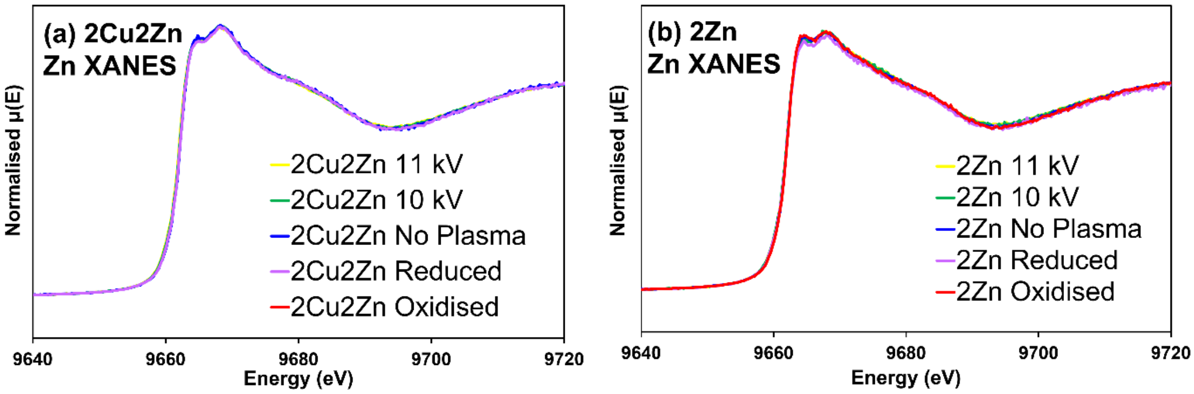

Supplementary Figure 33. Showing the similarity of the Zn K-edge XAS data for (a) 2Cu2Zn and (b) 2Zn. No Plasma, 10 kV and 11 kV were collected under reactive conditions (CO_2+H_2), whereas Pre-reduced and Reduced were collected under argon.

Supplementary Table 13: Zn K-edge EXAFS fitting parameters for 2Zn, under different conditions, highlighting the insignificant change.

System	R-factor	ΔE (eV)	Path	Coordination Number	Bond Distance (Å)	Debye-Waller Factor
2Zn 11 kV	0.029	2.7 ± 1.9	Zn-O	3.22 ± 0.47	1.94 ± 0.02	0.006 ± 0.002
2Zn 10 kV	0.032	3.4 ± 1.9	Zn-O	3.20 ± 0.47	1.94 ± 0.02	0.006 ± 0.002
2Zn No Plasma	0.023	3.2 ± 1.6	Zn-O	3.31 ± 0.40	1.94 ± 0.01	0.005 ± 0.002
2Zn Reduced	0.037	3.9 ± 1.9	Zn-O	3.23 ± 0.48	1.94 ± 0.02	0.004 ± 0.002
2Zn Pre-reduced	0.028	3.4 ± 1.7	Zn-O	3.44 ± 0.47	1.94 ± 0.01	0.005 ± 0.002

Supplementary Table 14: Zn K-edge EXAFS fitting parameters for 2Cu2Zn, under different conditions, highlighting the insignificant change.

System	R-factor	ΔE (eV)	Path	Coordination Number	Bond Distance (Å)	Debye-Waller Factor
2Cu2Zn 11 kV	0.030	2.6 ± 1.8	Zn-O	3.34 ± 0.47	1.93 ± 0.01	0.006 ± 0.002
2Cu2Zn 10 kV	0.032	2.5 ± 2.0	Zn-O	3.51 ± 0.53	1.93 ± 0.02	0.007 ± 0.002
2Cu2Zn No Plasma	0.044	3.7 ± 2.1	Zn-O	3.41 ± 0.55	1.94 ± 0.02	0.005 ± 0.002
2Cu2Zn Reduced	0.041	2.9 ± 2.0	Zn-O	3.23 ± 0.50	1.94 ± 0.02	0.004 ± 0.002
2Cu2Zn Pre-reduced	0.036	2.5 ± 1.9	Zn-O	3.46 ± 0.52	1.94 ± 0.02	0.005 ± 0.002

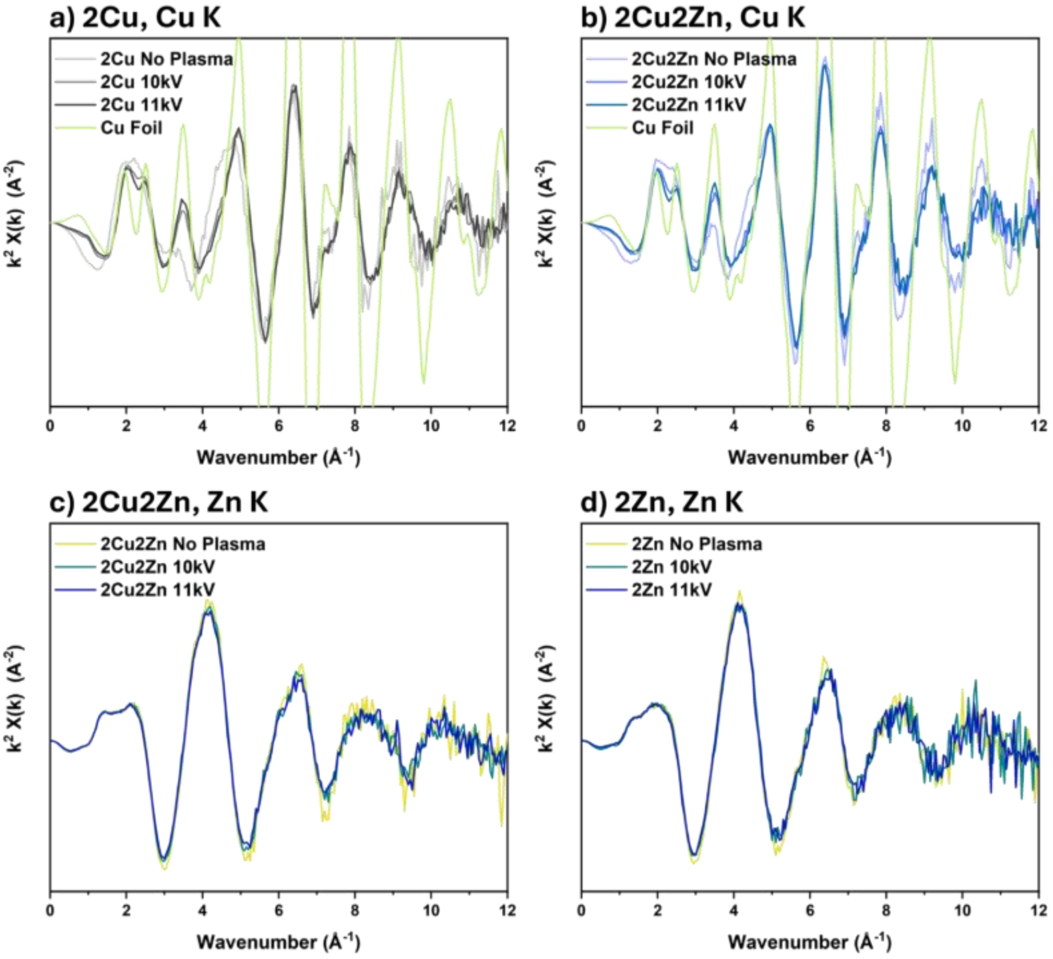

Supplementary Figure 34. The effect of applied voltage/power on the Cu or Zn environment in CO_2 hydrogenation under steady-state NTP conditions: k^2 weighted x(k) data of (a) 2Cu, (b-c) 2Cu2Zn and (d) 2Zn. (Experimental conditions: NTP system: at 10 and 11 kV, corresponding to 3.0 and 4.4 W; gas feed = 20%CO_2/60%H_2/Ar; total flow rate = 20 ml min^{-1}).

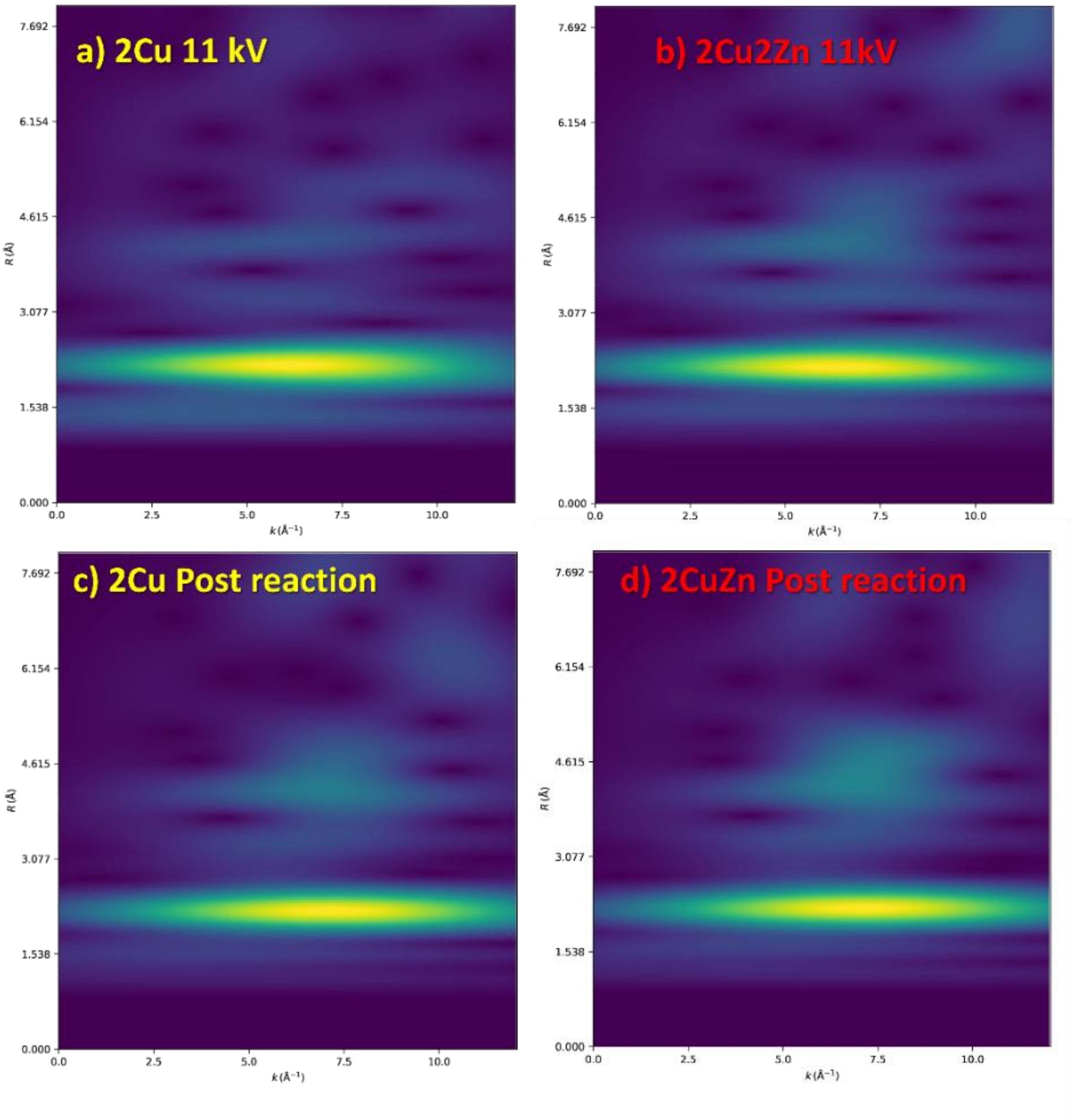

Supplementary Figure 35. The wavelet transforms for the Cu K-edge EXAFS spectra over (a, c) 2Cu under (a) CO_2 hydrogenation conditions (NTP: 11 kV, 4.5 W; gas feed = 20%CO_2/60%H_2/Ar; total flow rate = 20 ml min^{-1}), (b) post reaction and over (b, d) 2Cu2Zn under (b) CO_2 hydrogenation conditions and (d) post reaction.

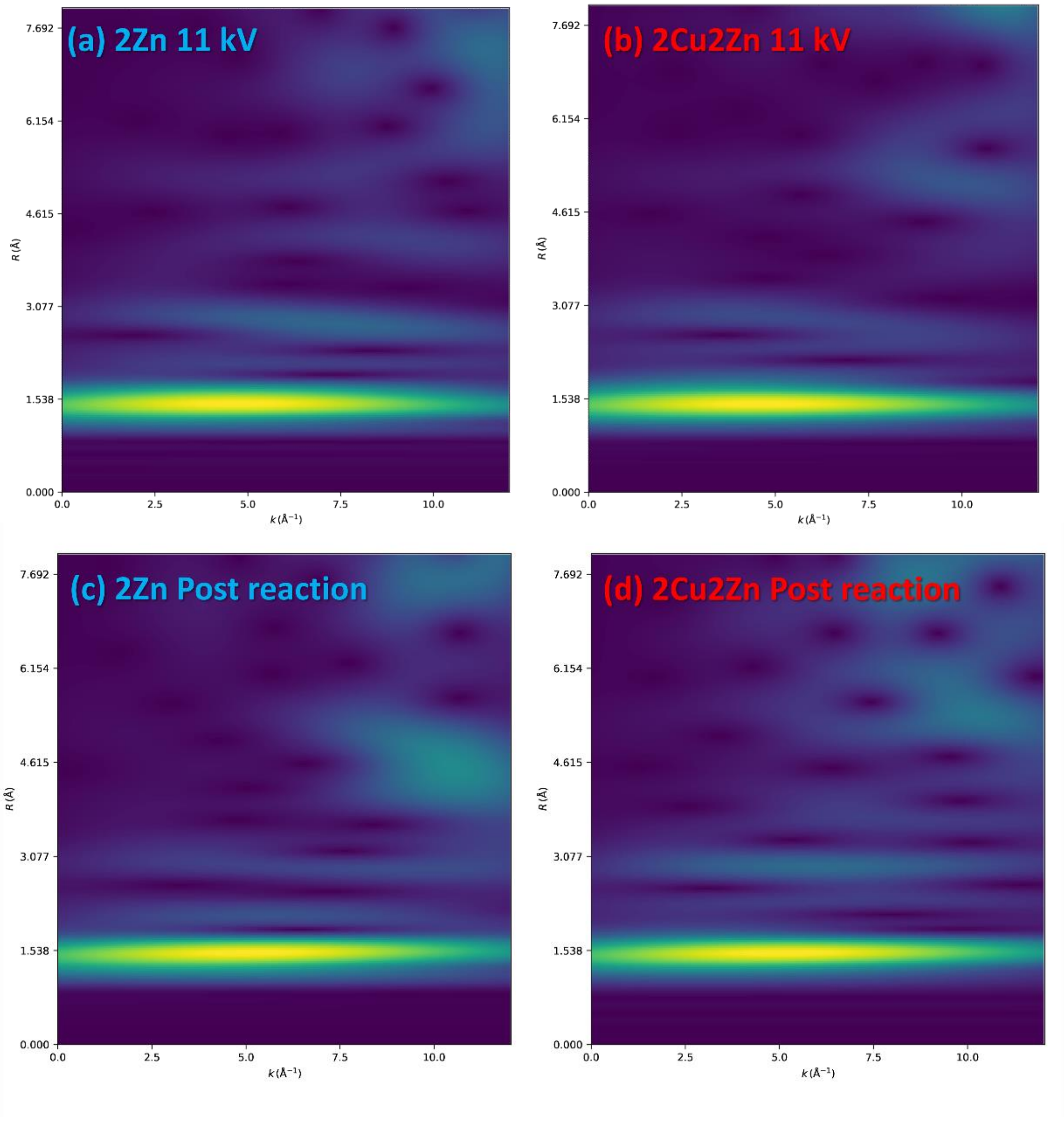

Supplementary Figure 36. The wavelet transforms for the Zn K-edge EXAFS spectra over (a, c) 2Zn under (a) CO_2 hydrogenation conditions (NTP: 11 kV, 4.5 W; gas feed = 20%CO_2/60%H_2/Ar; total flow rate = 20 ml min^{-1}), (b) post reaction and over (b, d) 2Cu2Zn under (b) CO_2 hydrogenation conditions and (d) post reaction.

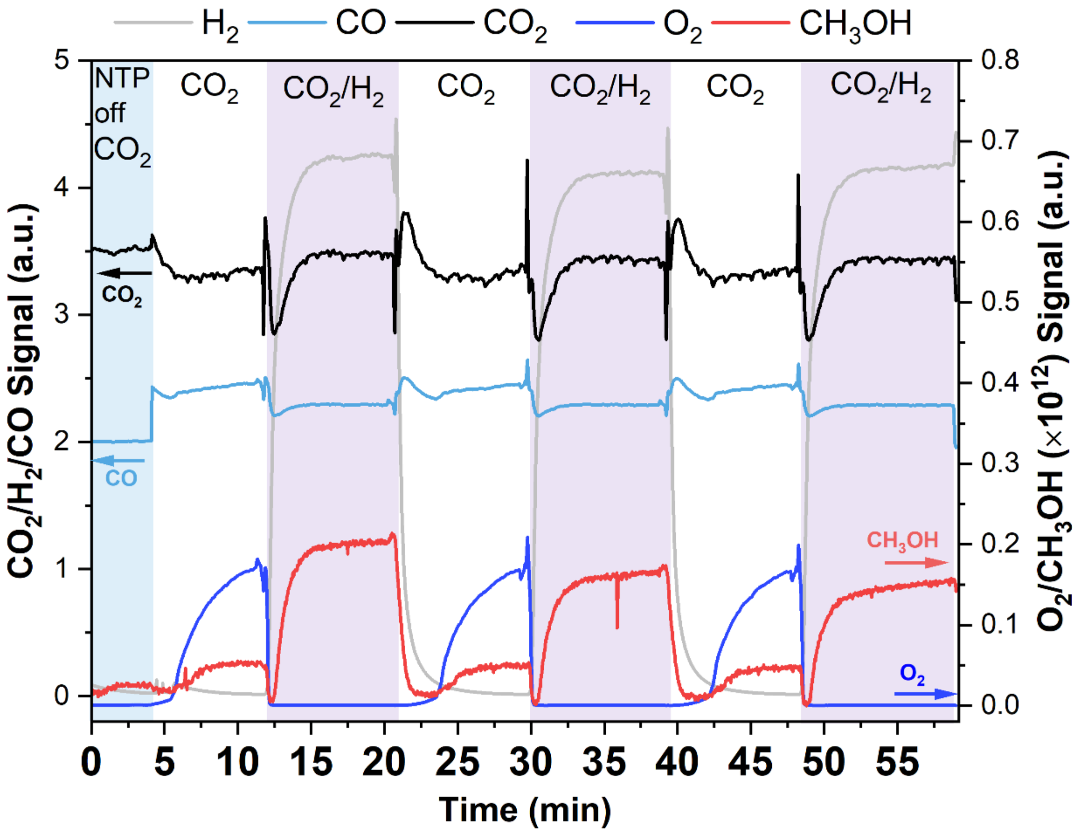

Supplementary Figure 37. Three cycles of MS signals collected from the transient *operando* XAS experiments as a function of time over the 2Cu/ZSM-5 catalyst during the NTP catalysis of switching the gas feed between CO_2/Ar and CO_2/H_2/Ar (gas feed = 20%CO_2/Ar and 20%CO_2/60%H_2/Ar; total flow rate = 20 ml min^{-1}) at 10 kV (corresponding to 3.0 W).

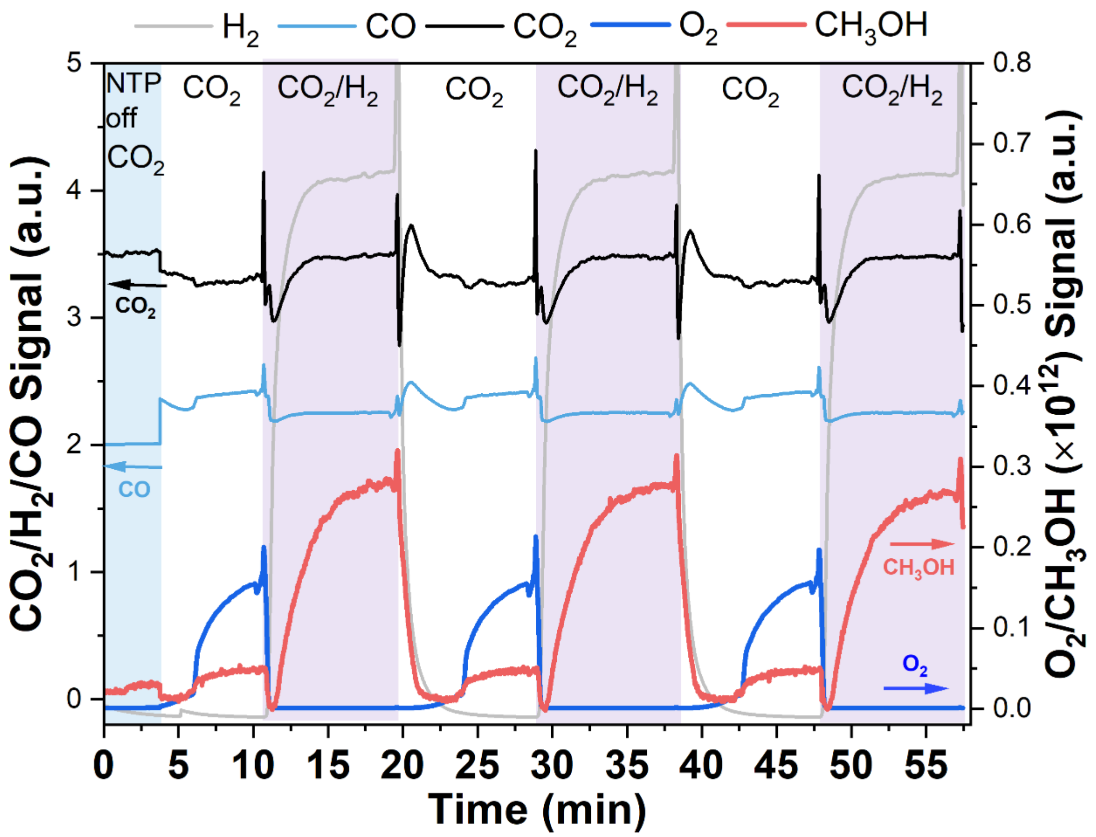

Supplementary Figure 38. Three cycles of MS signals collected from the transient *operando* XAS experiments as a function of time over the 2Cu2Zn/ZSM-5 catalyst during the NTP catalysis of switching the gas feed between CO_2/Ar and CO_2/H_2/Ar (gas feed = 20%CO_2/Ar and 20%CO_2/60%H_2/Ar; total flow rate = 20 ml min^{-1}) at 10 kV (corresponding to 3.0 W).

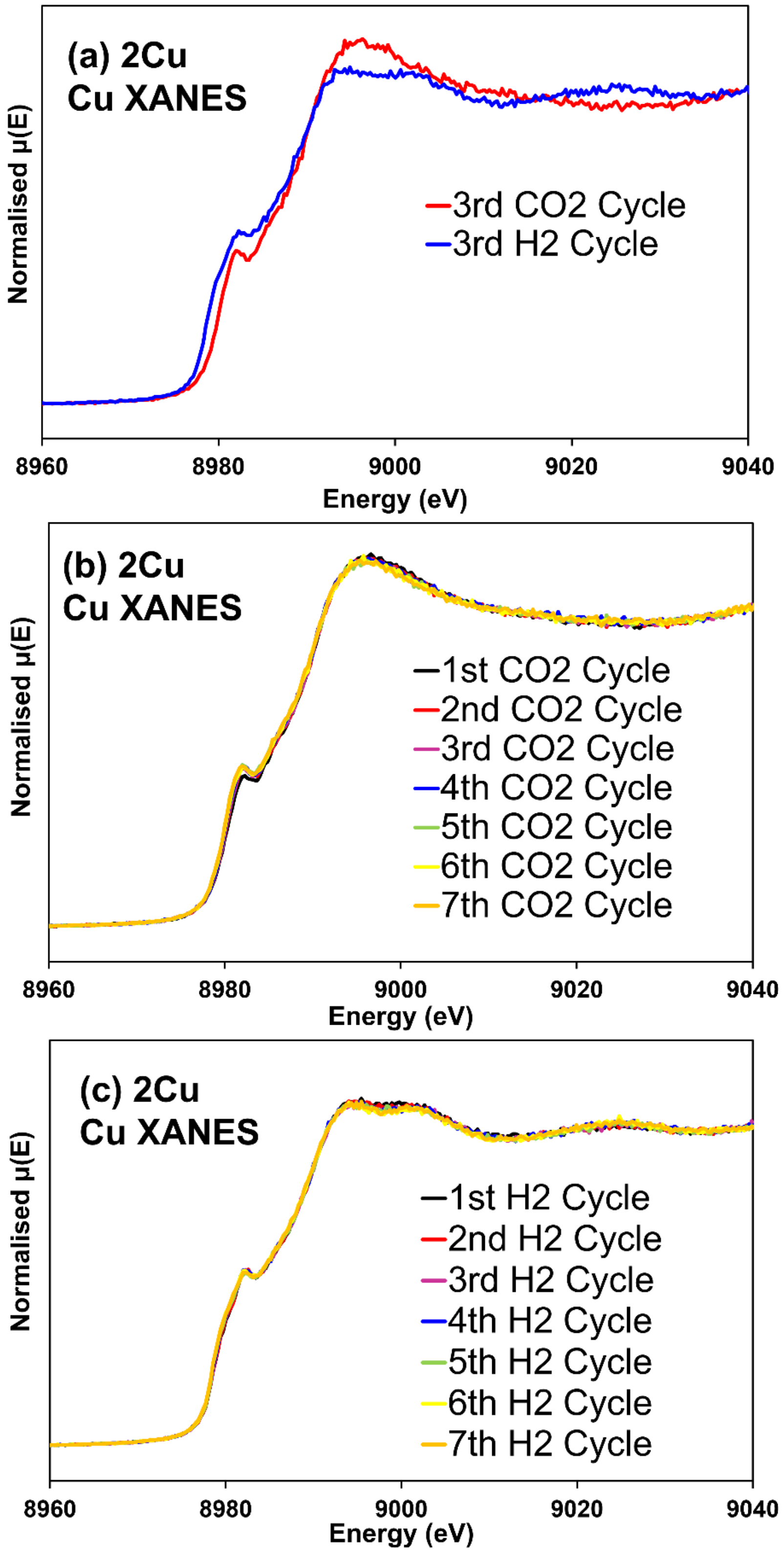

Supplementary Figure 39. Showing Cu K-edge XAS data of 2Cu highlighting (a) the oxidation-reduction behaviour, and the repeatability and resilience over repeated cycles of being exposed to (b) CO_2 only environment and (c) CO_2/H_2 environment, under 10 kV of NTP conditions.

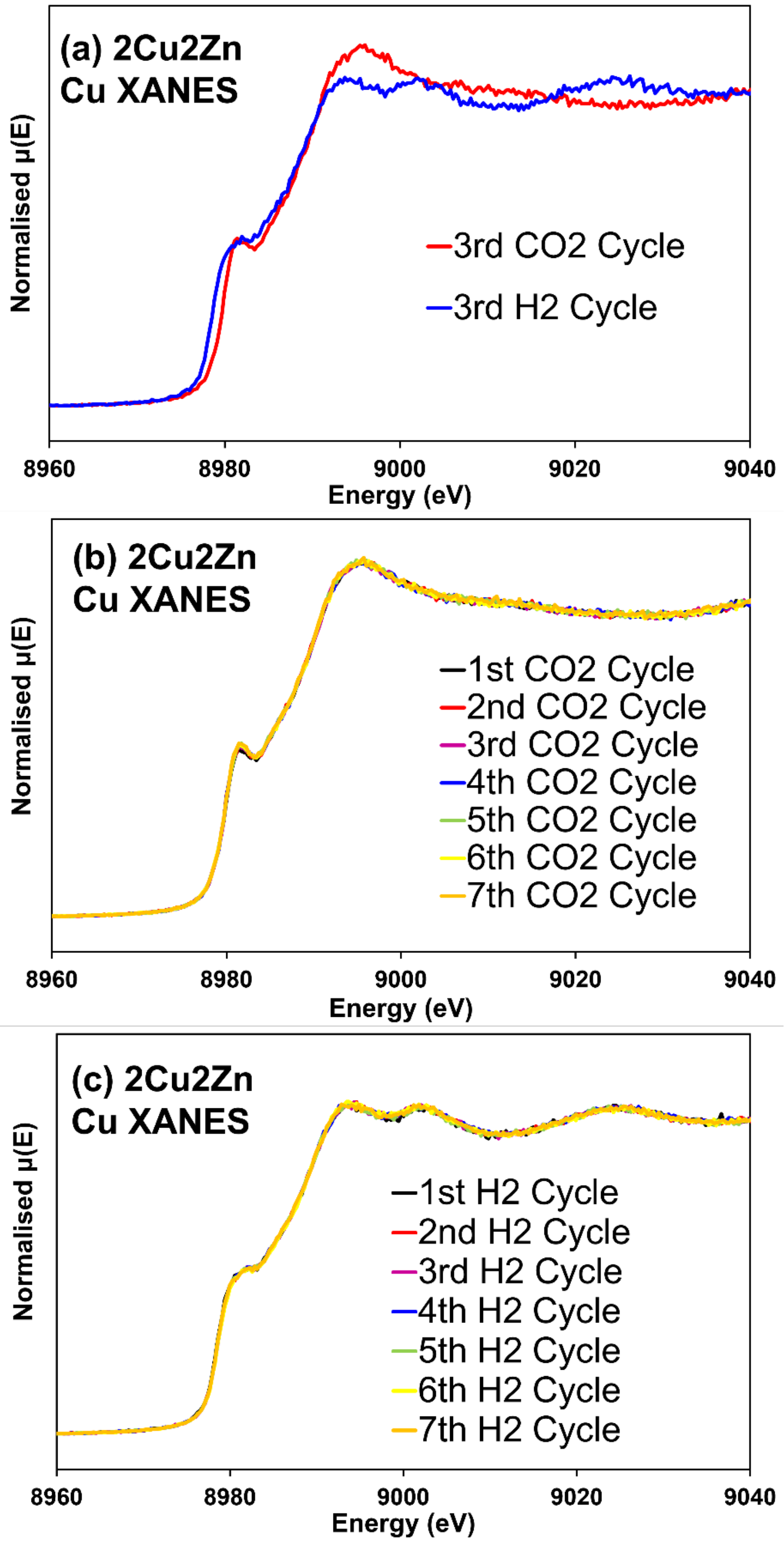

Supplementary Figure 40. Showing Cu K-edge XAS data of 2Cu2Zn highlighting (a) the oxidation-reduction behaviour, and the repeatability and resilience over repeated cycles of being exposed to (b) CO_2 only environment and (c) CO_2/H_2 environment, under 10 kV of NTP conditions.

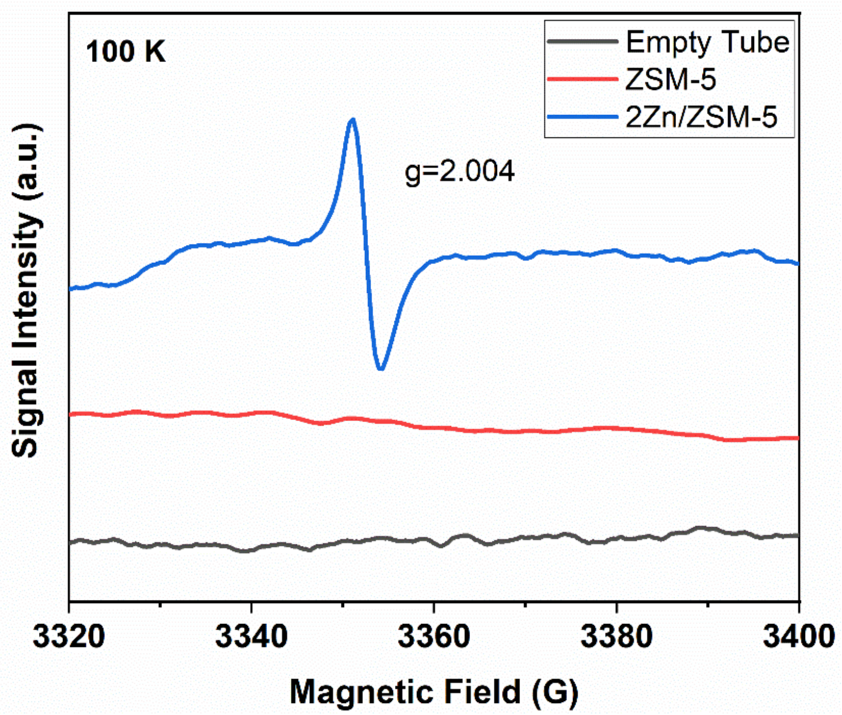

Supplementary Figure 41. X-band (9.4 GHz) EPR spectra at 100 K of the empty tube, ZSM-5 support and 2Zn catalyst.

Supplementary Note 4. Operando DRIFTS-MS for NTP-catalytic CO_2 hydrogenation to methanol.

Steady-state DRIFTS. On exposure to the reaction gas mixture (2% CO_2/6% H_2 in Ar), before plasma ignition, both ZSM-5, 2Cu and 2CuZn show bands at 1690−1694, 1620 and 1324−1372 cm^{-1} (Supplementary Fig. 44), corresponding to the bidentate carbonate.[18] Here, the observed bidentate carbonate may be located on the ZSM-5 support or the interface between Cu and the ZSM-5 support.[19, 20]

For ZSM-5 support (Supplementary Fig. 45), once the NTP is ignited, the bands at 1197 cm^{-1}, corresponding to bicarbonate, and the bands at 1265 and 1305 cm^{-1}, attributed to bridged carbonate, gradually increases as the reaction proceeds, implying that plasma-induced vibrationally excited CO_2 species adsorbed on the surface to form carbonate.[21, 22] It is note that the bidentate carbonate at 1616 and 1375 cm^{-1} was consumed, suggesting its reaction with H* species. In addition, the bands at 1559−1600 cm^{-1}, which are assigned to the formate species, were increasing, suggesting that surface carbonate species can be reacted with plasma generated H* species (in the gas phase, through Eley-Rideal mechanism) to form the formate species for methanol formation. Small broad bands at 1943 and 1850 cm^{-1}, corresponding to bridged adsorbed CO species, were observed. This explained the ZSM-5 support activity with high CO selectivity and 7.1% methanol selectivity in Fig. 1a. In the OH region, silanol hydroxyls at 3701 cm^{-1} and isolated hydroxyls at 3600 cm^{-1},[23] decreased with time elapsing, which can be attributed to the reaction with plasma activated H species to form water. Once NTP is off, formate and CO species will disappear within 3 min, while some carbonate species was accumulated on the catalyst support after 15 min purging.

For 2Cu, upon plasma ignition (Supplementary Figs. 46a–46b), small peaks at 2111 and 1856 cm^{-1}, assigned to linearly and bridged adsorbed CO on Cu^0, respectively, were observed, suggesting weak interactions between Cu and CO species.[24] The bands at 1204 and 1276 cm^{-1}, which can be attributed to the bicarbonate or bridged carbonate from vibrationally excited CO_2 adsorption[23], gradually increases as the reaction proceeds, similar with the ZSM-5 support. The bands at 1578 and 1405−1440 cm^{-1}, corresponding to υ_{as} (OCO) and υ_s (OCO) from formate species (HCOO*), appeared and increased with reaction time.[25, 26] Once plasma is off (Supplementary Fig. 46d), linearly adsorbed CO species disappeared immediately within 30s, while the formate species slowly decreased. The carbonate species decreased gradually and remained constant, indicating some accumulated carbonate species on the catalyst surface. In the OH region, the bands at 3699 and 3599 cm^{-1} (corresponding to silanol and isolated hydroxyls on the support) decreased with time, which is due to the reactions with H species to form water. It is note that the intensity of bands at 1690 and 1340 cm^{-1} (from bidentate carbonate, Supplementary Fig. 46b, 1340 cm^{-1} shifted to 1329 cm^{-1}) shows minor changes regardless of NTP state, suggesting that they are spectator species for the reaction. Accordingly, we speculate the formate

pathway for methanol synthesis via the NTP catalysis over the 2Cu catalyst, agreeing with previous results from thermal catalytic CO_2 hydrogenation to methanol.[20, 25]

Transient cycling DRIFTS. Cycling experiments in which the feed was switched between $H_2/CO_2/Ar$ and H_2/Ar (Supplementary Fig. 49) under NTP conditions were conducted over 2Cu2Zn catalyst to identify the role of CO species in the reaction mechanism. As shown in Supplementary Fig. 49a and 49c, when NTP is on with $H_2/CO_2/Ar$ gas flowing (cycles 1 and 2), the bands of adsorbed CO species and formyl species at 1691 cm^{-1} showed the similar increasing trend. After switching to H_2/Ar (Supplementary Fig. 49b and 49d), CO species and formyl species disappeared quickly with similar rate (within 60 s here). This confirmed that formyl species originated from hydrogenation of surface CO species. Regarding the formate species (at 1565−1605 cm^{-1}), after switching to H_2/Ar (cycles 1 and 2), the intensity of bands decreased gradually (with a slower rate than formyl), while the feed gas switched back to $H_2/CO_2/Ar$ gas, the formate species increased again, suggesting that they are the active species for CO_2 hydrogenation.

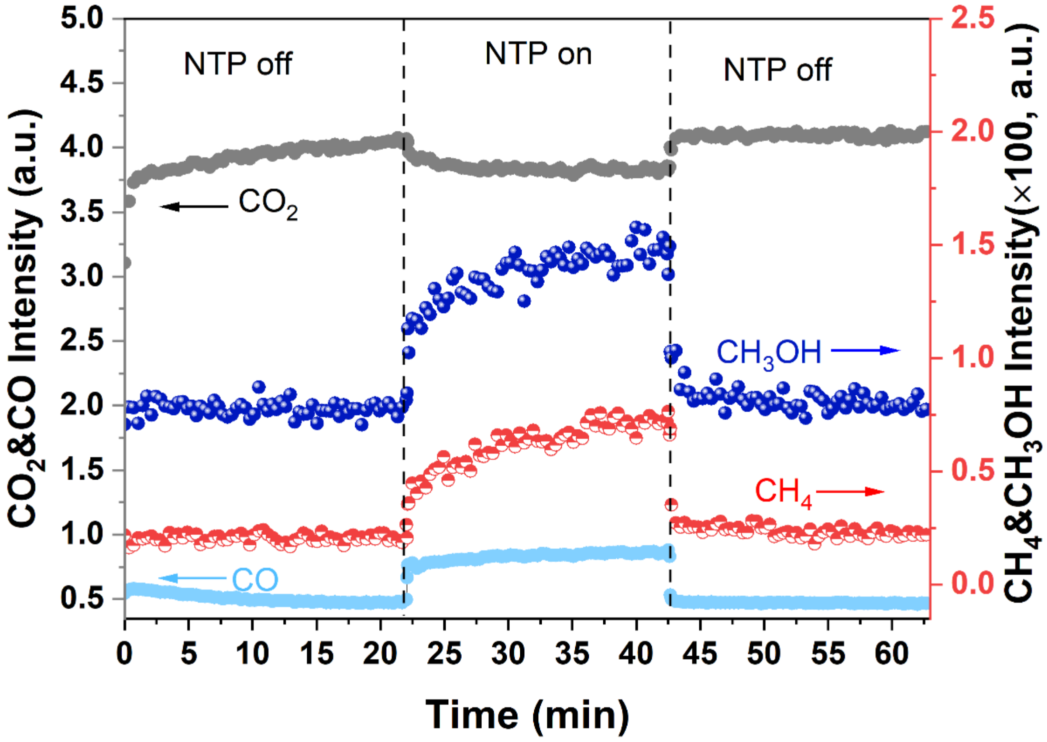

Supplementary Figure 42. (a) MS signals collected from the *operando* DRIFTS experiments as a function of time during NTP-assisted CO_2 hydrogenation over the 2Cu/ZSM-5 catalyst.

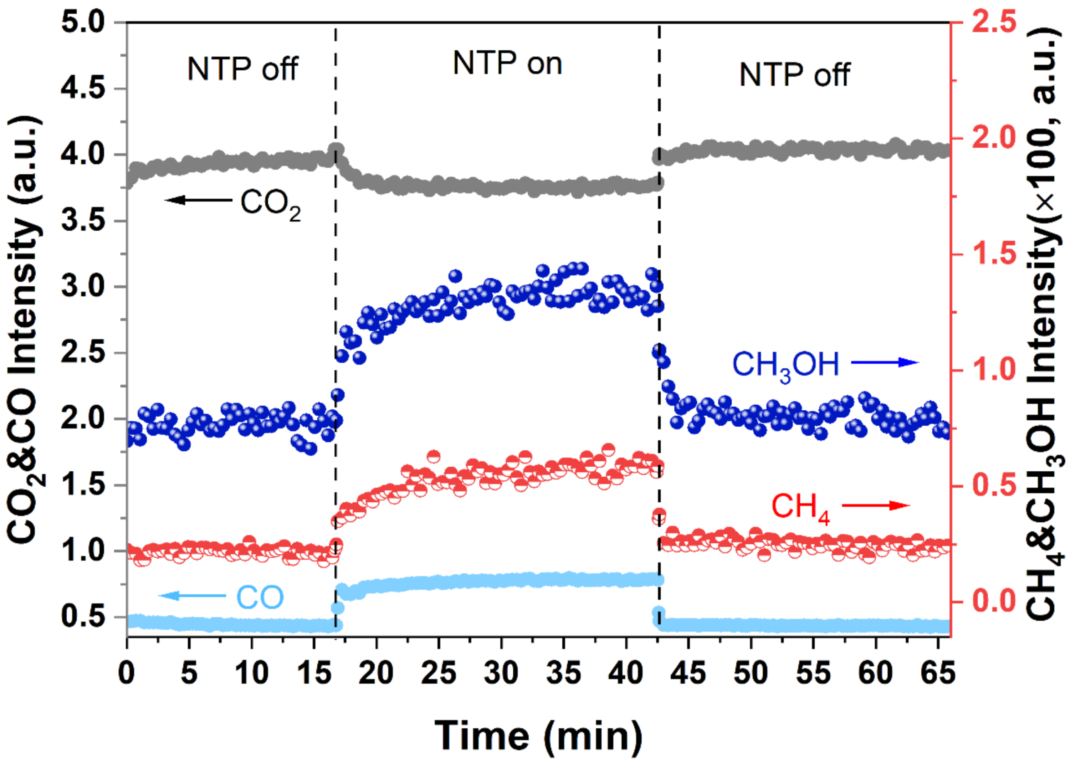

Supplementary Figure 43. MS signals collected from the *operando* DRIFTS experiments as a function of time during NTP-assisted CO_2 hydrogenation over the 2Cu2Zn/ZSM-5 catalyst.

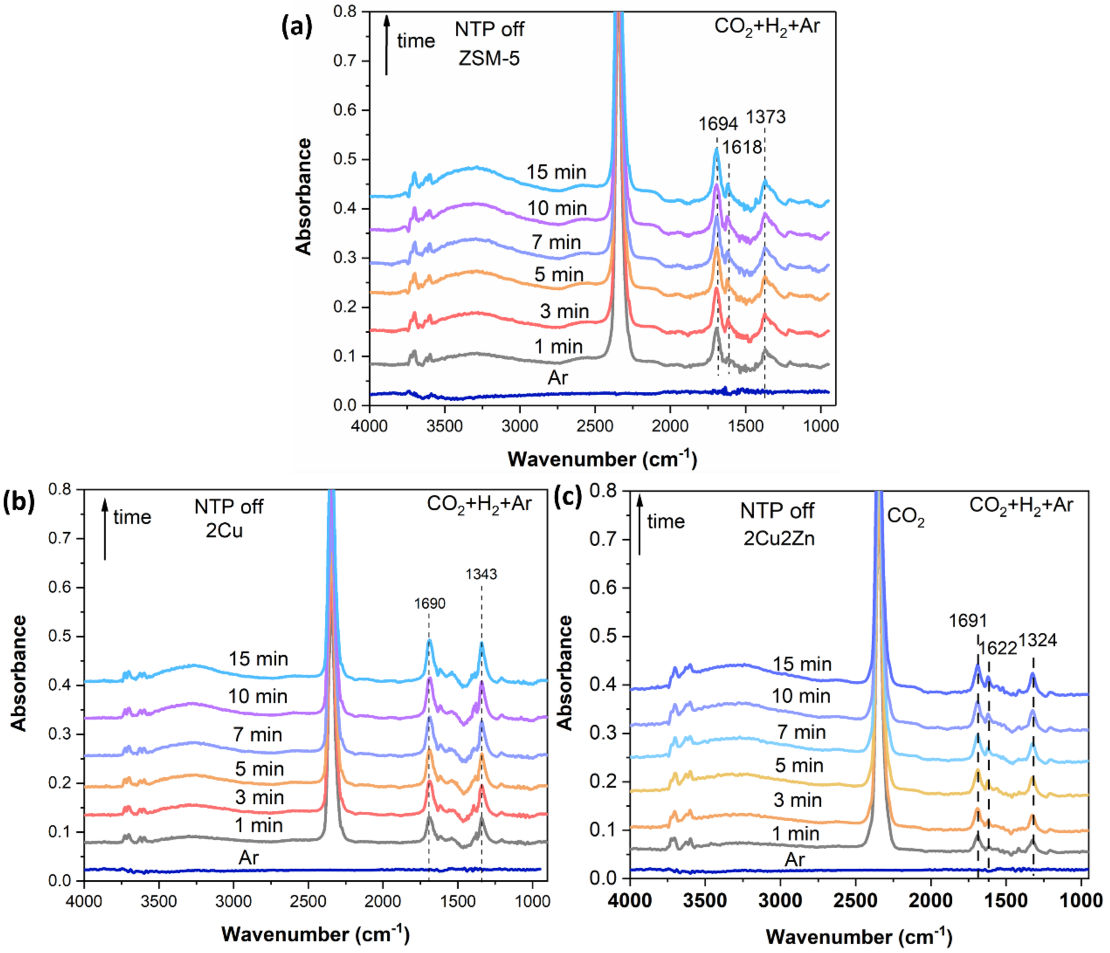

Supplementary Figure 44. Steady-state DRIFTS spectra of CO_2 hydrogenation over (a) ZSM-5, (b) 2Cu and (c) 2Cu2Zn catalysts under the reaction gas of 2% CO_2 + 6% H_2 + Ar with NTP off.

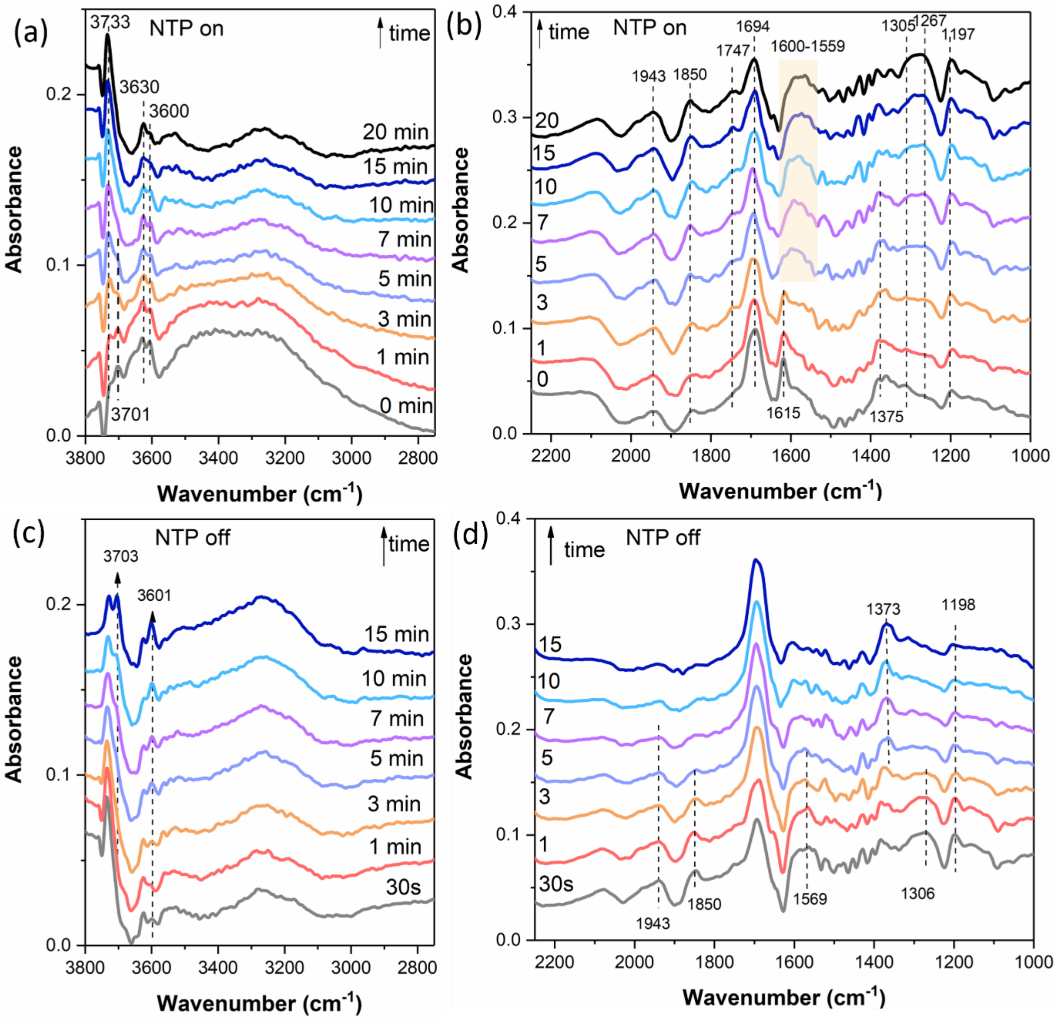

Supplementary Figure 45. Steady-state DRIFTS spectra for CO_2 hydrogenation over the ZSM-5 support under the reaction gas of 2% CO_2 + 6% H_2 + Ar: (a-b) NTP-on condition at 5.0 kV and 27.0 kHz, and (c-d) NTP-off condition after reaction.

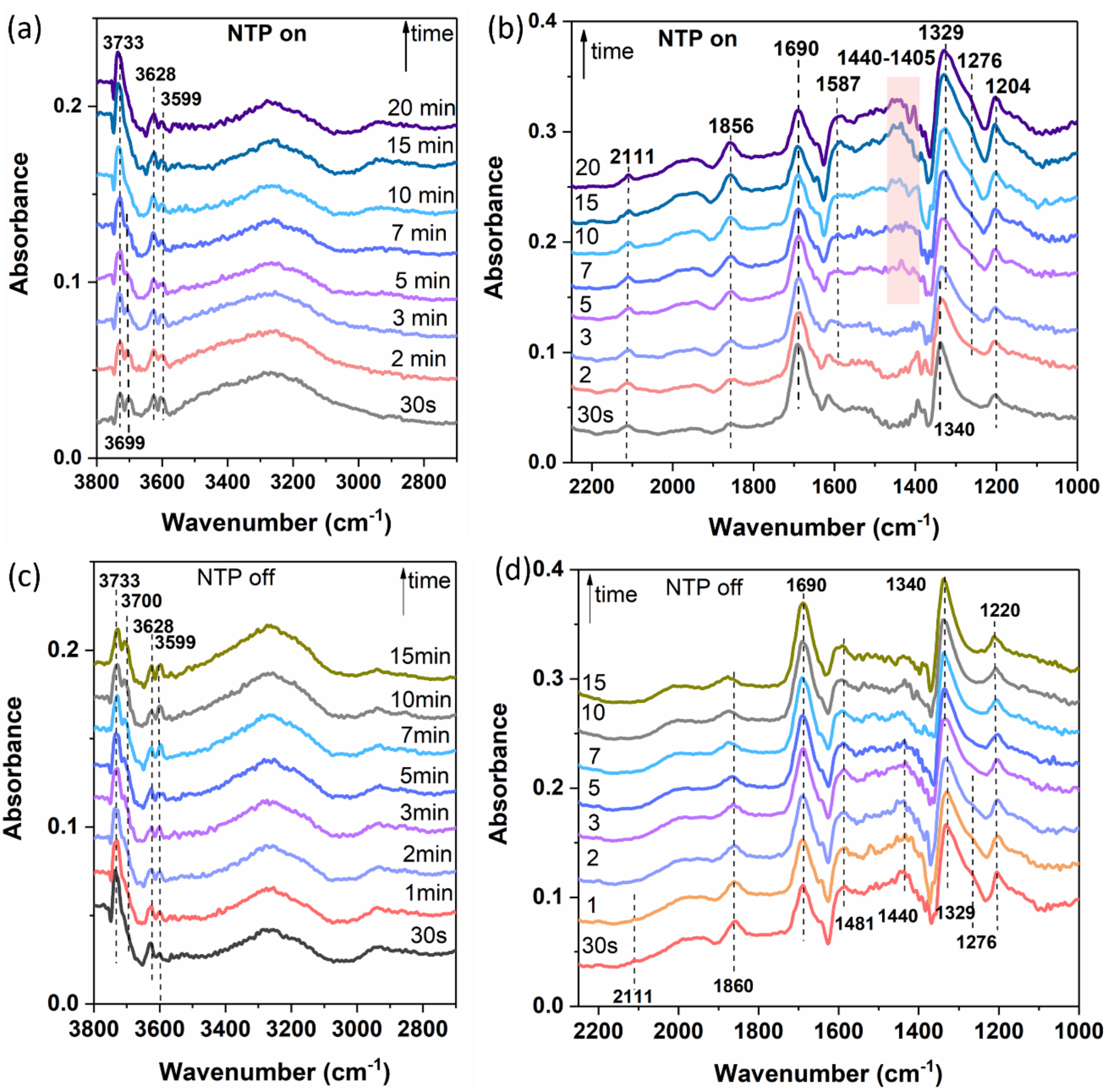

Supplementary Figure 46. *Operando* steady-state DRIFTS spectra for CO_2 hydrogenation over the 2Cu catalyst under the reaction gas of 2% CO_2 + 6% H_2 + Ar: (a-b) NTP-on condition at 5.0 kV and 27.0 kHz, and (c-d) NTP-off condition after reaction.

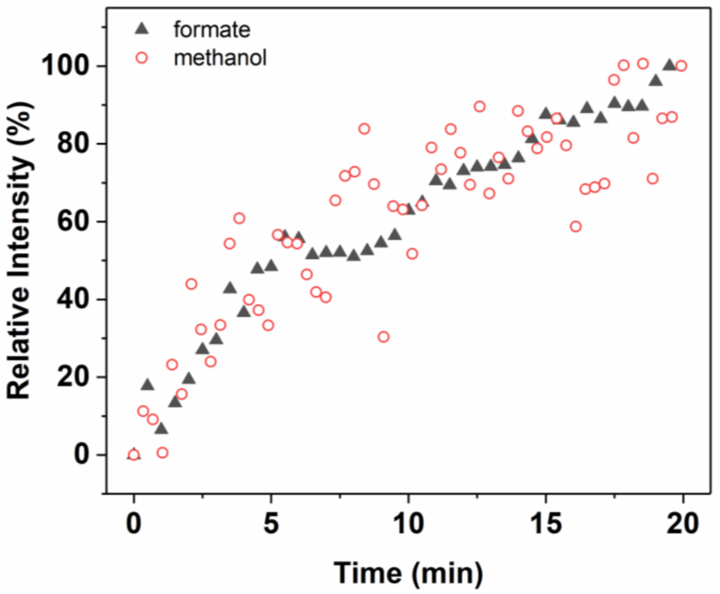

Supplementary Figure 47. The relative intensity of formate species and methanol as a function of time over 2Cu catalyst with NTP on under steady state from Supplementary Fig. 38 (peak area was normalised to 100% observed at the end of experiments).

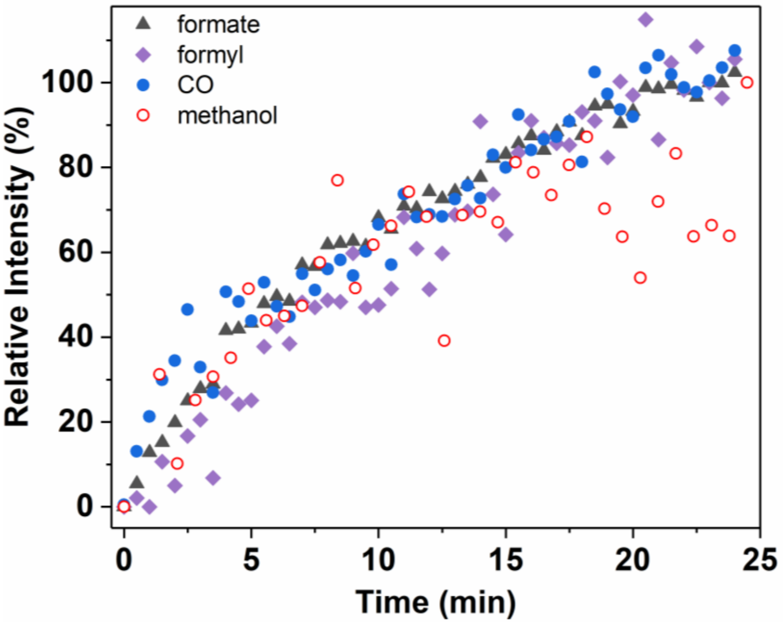

Supplementary Figure 48. The relative intensity of surface CO, formyl, formate species and methanol as a function of time over 2Cu2Zn catalyst with NTP on from Fig. 5 (peak area was normalised to 100% observed at the end of experiments).

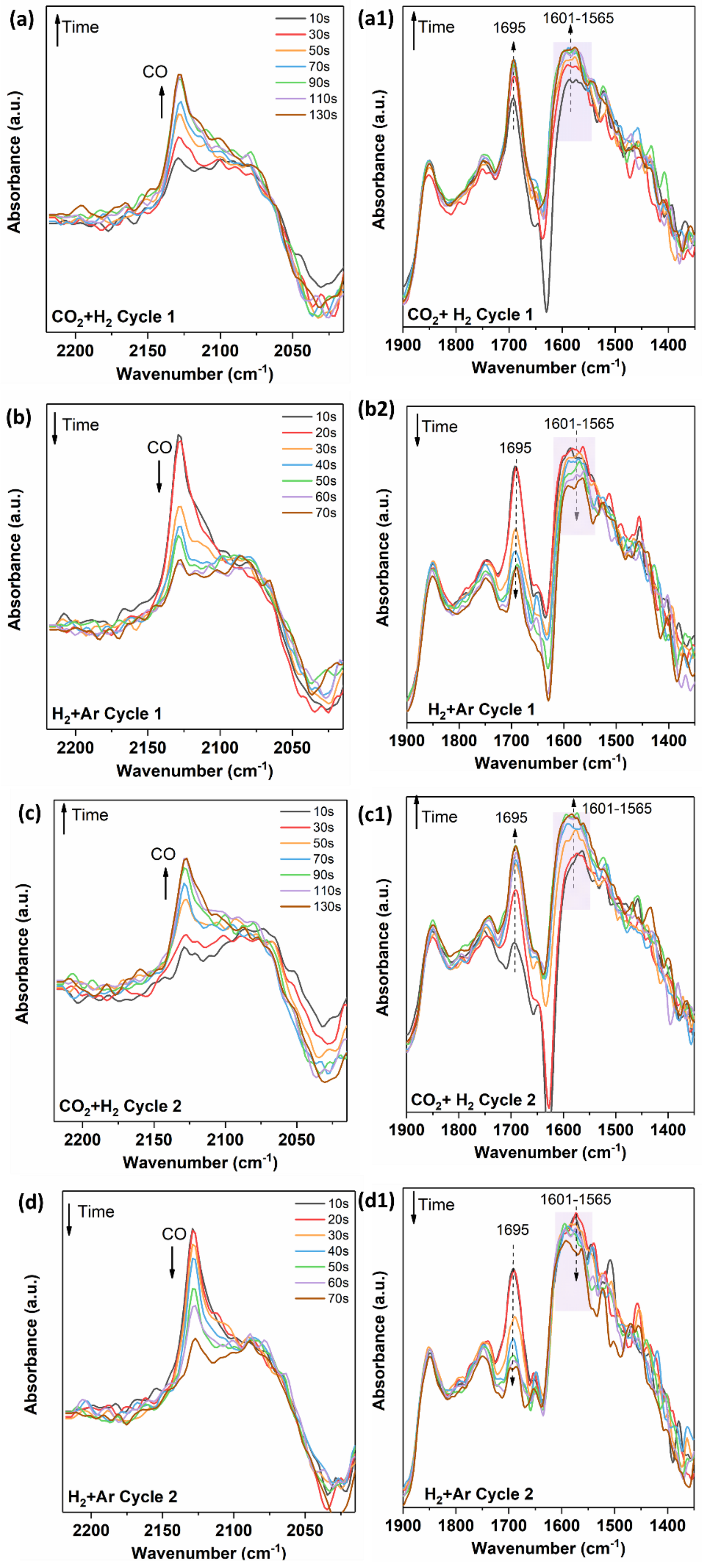

Supplementary Figure 49. (a) *In situ* transient cycling DRIFTS spectra of surface species for CO_2 hydrogenation over the 2Cu2Zn/ZSM-5 catalyst under plasma conditions switching between (a,c) 2% CO_2 + 6% H_2 + Ar and (b, d) 6% H_2 + Ar (Condition: 10 kV, 27.2 kHz).

Supplementary Table 15: The assignment of the DRIFTS wavelengths of the surface species.[18, 20, 22-25, 27-31]

Surface species	Wavenumbers (cm^{-1})	Assignment
CO*	2127, 2117	Linearly adsorbed
	1946−1988, 1860	Bridge adsorbed
Bidentate carbonate	1690, 1622	υ_{as} (OCO)
	1324−1340	υ_{s} (OCO)
bicarbonate	1200−1230	δ (OH)
	1441−1460	υ_{s} (OCO)
	1603	υ_{as} (OCO)
	3625−3628	υ_{s} (OH)
Bridged carbonate	1742−1752	υ_{as} (OCO)
	1267−1300	υ_{s} (OCO)
Formate	1554, 1540−1605, 1587, 1576-1603	υ_{as} (OCO)
	1400−1440	υ_{s} (OCO)
Formyl	1691	υ_{s} (C=O)
Methoxy	1153	ν(O-C) of b-OCH_3
Silanol hydroxyls	3733	-OH
Isolated hydroxyls	3600−3630	-OH

Supplementary Note 5. Calculation of plasma discharge power.

All plasma discharge power ($P_{\text{discharge}}$) and specific input energy (SIE) calculations in this work used the Q-V Lissajous graph (Supplementary Fig.50) using Eqs. S1–S2 according to the methods published elsewhere.[32] A capacitor (10 nF) as a monitor was connected in the circuit to quantify the electric charge (Q(t)) by measuring the voltage at both ends of the capacitor (V_m (t)). As shown in Supplementary Fig. 51, a Q-V Lissajous plot could be obtained by collecting the voltage between the high voltage electrode and ground electrode as X axis data (V(t)).

$$P(\text{discharge}) = \int_{t_0}^{t_1} V(t)I(t)dt \tag{S1}$$

$$I(t)=\frac{dQ(t)}{dt}=C_m\frac{dV_m(t)}{dt} \tag{S2}$$

$$SIE = \frac{P_{\text{discharge}}}{F_{total}} \tag{S3}$$

where V(t) is the applied voltage, C_m is the value of capacitor, V_m (t) is the voltage between two ends of capacitor, and Q (t) is the quantity of transported charges which changes with the applied voltage. F_{Total} is the total gas flow of feed.

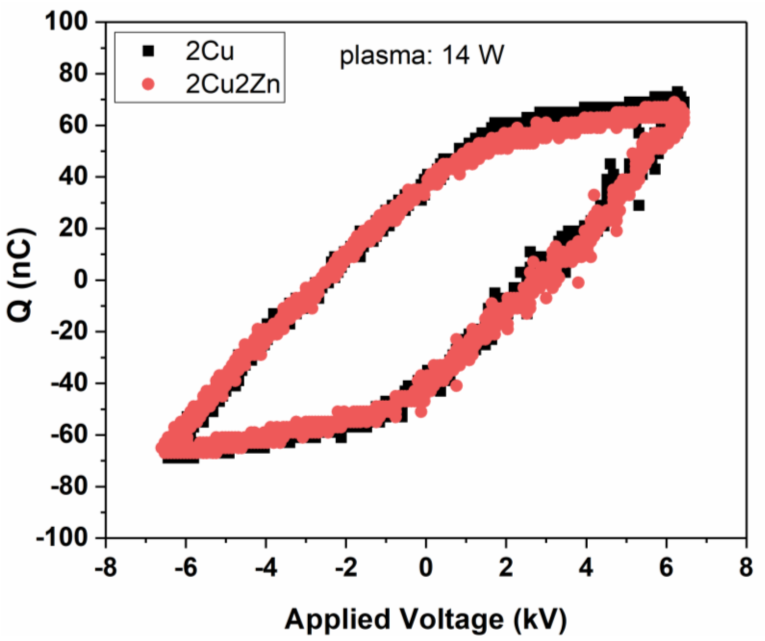

Supplementary Figure 50. Q-V Lissajous graphs under NTP at 14 W over the 2Cu and 2Cu2Zn catalysts.

Supplementary References

1. Ding, H. et al. Precise Confinement and Position Distribution of Atomic Cu and Zn in ZSM-5 for CO_2 Hydrogenation to Methanol. *Nanomaterials (Basel)* **13** (2023).
2. Pan, T. et al. Synthesis and consequence of Zn modified ZSM-5 zeolite supported Ni catalyst for catalytic aromatization of olefin/paraffin. *Fuel* **311** (2022).
3. Koike, N. et al. Increasing the ion-exchange capacity of MFI zeolites by introducing Zn to aluminosilicate frameworks. *Dalton Trans* **47**, 9546-9553 (2018).
4. Bae, Y.-S., Yazaydın, A.O.z.r. & Snurr, R.Q. Evaluation of the BET method for determining surface areas of MOFs and zeolites that contain ultra-micropores. *Langmuir* **26**, 5475-5483 (2010).
5. Schott, V. et al. Chemical activity of thin oxide layers: strong interactions with the support yield a new thin-film phase of ZnO. *Angew Chem Int Ed Engl* **52**, 11925-11929 (2013).
6. Kuld, S., Conradsen, C., Moses, P.G., Chorkendorff, I. & Sehested, J. Quantification of zinc atoms in a surface alloy on copper in an industrial-type methanol synthesis catalyst. *Angewandte Chemie International Edition* **53**, 5941-5945 (2014).
7. Großmann, D., Klementiev, K., Sinev, I. & Grünert, W. Surface Alloy or Metal–Cation Interaction-The State of Zn Promoting the Active Cu Sites in Methanol Synthesis Catalysts. *ChemCatChem* **9**, 365-372 (2016).
8. Ye, R. et al. A Ce-CuZn catalyst with abundant Cu/Zn-O_V-Ce active sites for CO_2 hydrogenation to methanol. *Nature Communications* **15**, 2159 (2024).
9. Wang, J. et al. Boosting CO_2 hydrogenation to methanol via Cu-Zn synergy over highly dispersed Cu, Zn-codoped ZrO_2 catalysts. *Catalysis Today* **410**, 205-214 (2023).
10. Shao, Y., Kosari, M., Xi, S. & Zeng, H.C. Single solid precursor-derived three-dimensional nanowire networks of CuZn-silicate for CO_2 hydrogenation to methanol. *ACS Catalysis* **12**, 5750-5765 (2022).
11. Wang, X. et al. Catalytic activity for direct CO_2 hydrogenation to dimethyl ether with different proximity of bifunctional Cu-ZnO-Al_2O_3 and ferrierite. *Applied Catalysis B: Environmental* **327**, 122456 (2023).
12. Luo, P. et al. Ternary synergistic interaction of Cu-ZnO-ZrO_2 promoting CO_2 hydrogenation to methanol. *Applied Catalysis A: General* **689**, 120006 (2025).
13. Zhu, J. et al. Flame synthesis of Cu/ZnO–CeO_2 catalysts: synergistic metal–support interactions promote CH_3OH selectivity in CO_2 hydrogenation. *ACS catalysis* **11**, 4880-4892 (2021).
14. Zhou, H. et al. Combining atomic layer deposition with surface organometallic chemistry to enhance atomic-scale interactions and improve the activity and selectivity of Cu–Zn/SiO_2 catalysts for the hydrogenation of CO_2 to methanol. *JACS Au* **3**, 2536-2549 (2023).
15. Liu, Y. et al. Hydrogenation of CO_2 to CH_3OH on the Cu–ZnO–$SrTiO_3$ Catalysts: The Electronic Metal–Support Interaction Induces Oxygen Vacancy Generation. *ACS Catalysis* **14**, 12610-12622 (2024).
16. Chen, H. et al. CO2 hydrogenation to methanol over Cu/ZnO/ZrO_2 catalysts: Effects of ZnO morphology and oxygen vacancy. *Fuel* **314**, 123035 (2022).
17. Newville, M. in Journal of Physics: Conference Series, Vol. 430 012007 (IOP Publishing, 2013).
18. Fukuda, Y. & Tanabe, K. Infrared study of carbon dioxide adsorbed on magnesium and calcium oxides. *Bulletin of the Chemical Society of Japan* **46**, 1616-1619 (1973).
19. Chen, H. et al. Coupling non-thermal plasma with Ni catalysts supported on BETA zeolite for catalytic CO_2 methanation. *Catalysis Science & Technology* **9**, 4135-4145 (2019).
20. Wu, C. et al. Inverse ZrO_2/Cu as a highly efficient methanol synthesis catalyst from CO_2 hydrogenation. *Nat Commun* **11**, 5767 (2020).
21. Kim, D.Y. et al. Cooperative Catalysis of Vibrationally Excited CO_2 and Alloy Catalyst Breaks the Thermodynamic Equilibrium Limitation. *J Am Chem Soc* **144**, 14140-14149 (2022).
22. Coenen, K., Gallucci, F., Mezari, B., Hensen, E. & van Sint Annaland, M. An in-situ IR study on the adsorption of CO_2 and H_2O on hydrotalcites. *Journal of CO_2 Utilization* **24**, 228-239 (2018).

23. Eckle, S., Anfang, H.-G. & Behm, R.J.r. Reaction intermediates and side products in the methanation of CO and CO_2 over supported Ru catalysts in H_2-rich reformate gases. *The Journal of Physical Chemistry C* **115**, 1361-1367 (2011).
24. Schumann, J., Kröhnert, J., Frei, E., Schlögl, R. & Trunschke, A. IR-Spectroscopic Study on the Interface of Cu-Based Methanol Synthesis Catalysts: Evidence for the Formation of a ZnO Overlayer. *Topics in Catalysis* **60**, 1735-1743 (2017).
25. Clarke, D.B. & Bell, A.T. An infrared study of methanol synthesis from CO2 on clean and potassium-promoted Cu/SiO2. *Journal of Catalysis* **154**, 314-328 (1995).
26. Di Cosimo, J., Dıez, V., Xu, M., Iglesia, E. & Apesteguıa, C. Structure and surface and catalytic properties of Mg-Al basic oxides. *Journal of Catalysis* **178**, 499-510 (1998).
27. Kattel, S., Ramírez, P.J., Chen, J.G., Rodriguez, J.A. & Liu, P. Active sites for CO2 hydrogenation to methanol on Cu/ZnO catalysts. *Science* **355**, 1296-1299 (2017).
28. Zhang, S. et al. Revealing and Regulating the Complex Reaction Mechanism of CO_2 Hydrogenation to Higher Alcohols on Multifunctional Tandem Catalysts. *ACS Catalysis* **13**, 3055-3065 (2023).
29. Fehr, S.M., Nguyen, K. & Krossing, I. Realistic Operando-DRIFTS Studies on Cu/ZnO Catalysts for CO_2 Hydrogenation to Methanol – Direct Observation of Mono-ionized Defect Sites and Implications for Reaction Intermediates. *ChemCatChem* **14** (2021).
30. Ren, P. et al. Mechanism and sites requirement for CO hydrogenation to CH_3OH over Cu/CeO_2 catalysts. *Applied Catalysis B: Environmental* **305** (2022).
31. Szanyi, J. & Kwak, J.H. Dissecting the steps of CO_2 reduction: 1. The interaction of CO and CO_2 with gamma-Al_2O_3: an in situ FTIR study. *Phys Chem Chem Phys* **16**, 15117-15125 (2014).
32. Manley, T. The electric characteristics of the ozonator discharge. *Transactions of the electrochemical society* **84**, 83 (1943).
